# Database-aided UHPLC-Q-orbitrap MS/MS strategy putatively identifies 52 compounds from *Wushicha* Granule to propose *anti*-counterfeiting quality-markers for pharmacopoeia

**DOI:** 10.1186/s13020-023-00829-2

**Published:** 2023-09-09

**Authors:** Xican Li, Shaoman Chen, Jingyuan Zeng, Rongxin Cai, Yilan Liang, Chuanbin Chen, Ban Chen, Chunhou Li

**Affiliations:** 1 School of Chinese Herbal Medicines, Guangzhou University of Chinese Medicine, Guangzhou, 510006 China; 2 College of Pharmacy, Chengdu University of Traditional Chinese Medicine, Chengdu, 611137 China; 3 Key Laboratory of Fermentation Engineering (Ministry of Education), Cooperative Innovation Center of Industrial Fermentation (Ministry of Education and Hubei Province), Hubei University of Technology, Wuhan, 430068 China

**Keywords:** Adulteration, Glycyrrhetinic acid, Database UPLC-Q-orbitrap MS, *Wushi* Tea, Quality-marker, Quality-control

## Abstract

*Wushicha* Granule, an over-the-counter-drug (OTC) prescription, consists of 19 traditional Chinese herbals medicines (CHMs), such as Chaihu, Hongcha, Chuanxiong, Houpo, and Gancao. The five however have not been effectively characterized by the quality-markers (Q-markers) system in current Pharmacopoeia. The study therefore established a novel database-aided ultra-high performance liquid chromatography-quadrupole-orbitrap mass spectrometry (UHPLC-Q-orbitrap MS/MS) strategy. The strategy has putatively identified 52 compounds from *Wushicha* Granule, mainly including flavonoids, saponins, alkaloid, lignins, and lactones. Especially, saponin “glycyrrhetinic acid” in the Granule was specifically identified as 18β-configuration (rather than 18α-configuration). Meanwhile, two pairs of isomers were fully discriminated, including vitexin *vs* isovitexin and daidzein *vs* 7,4'-dihydroxyflavone. 8β-Glycyrrhetinic acid, together with saponin saikosaponin A, alkaloid caffeine, lactone *S*-senkyunolide A, and lignin magnolol, were further studied using quantum chemical calculation, UV–vis spectra, and *anti*-counterfeiting validation experiment. In the validation experiment, they have successfully recognized 6 counterfeit *Wushicha* Granules, by means of a LC–MS equipped extraction software. Based on these results, 8β-glycyrrhetinic acid is recommended to replace the old Q-marker “glycyrrhetinic acid”; while saikosaponin A, caffeine, *S-*senkyunolide A, and magnolol are recommended as new Q-markers. These recommendations can not only recognize the counterfeits regarding Chaihu, Hongcha, Chuanxiong, Houpo, and Gancao, but also prevent the possible safety-incident. All these will greatly improve the efficiency and specificity of current Pharmacopoeia.

**Supplementary Information:**

The online version contains supplementary material available at 10.1186/s13020-023-00829-2.

## Introduction

**Fig. 1:**
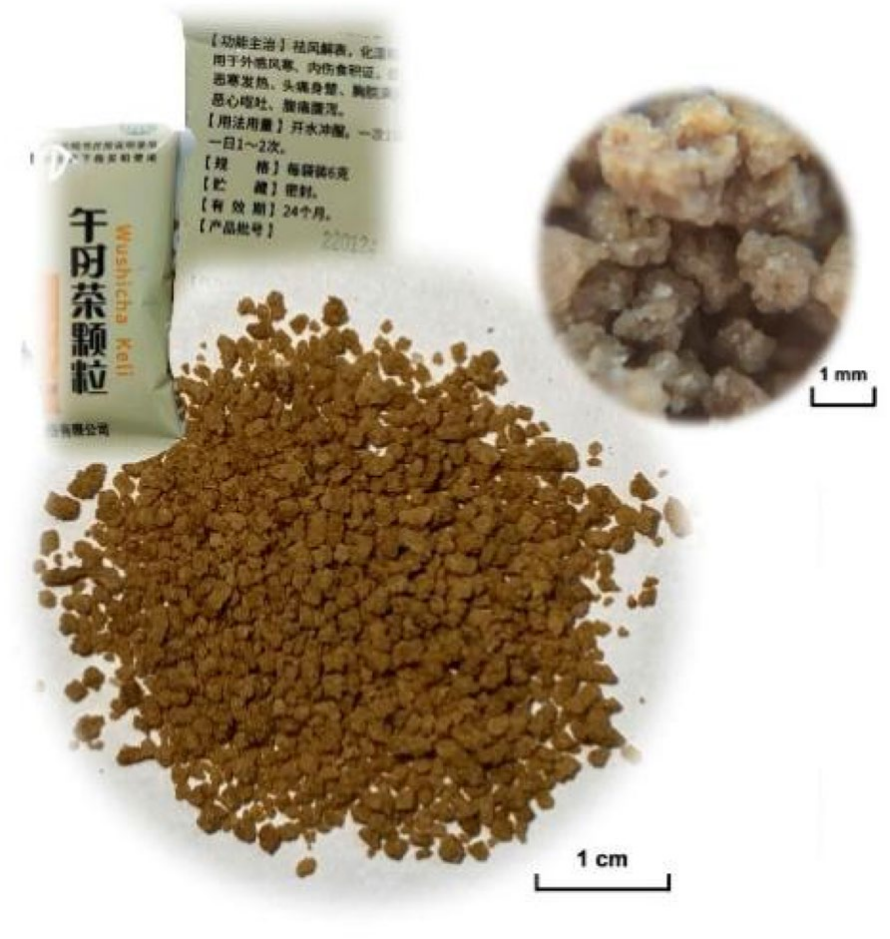
The photo of *Wushicha* Granule appearance (the left insert suggests packing information; the right insert is the enlarged view of granule)

*Wushicha (Wushi Tea,* 午時茶*,* Fig. 1) Granule is a Chinese traditional health-care prescription medicine with an over 200-year history. It is documented to be related to *Dragon Boat Festival* (端午節) in China [1, 2]. From the angle of traditional Chinese medicine (TCM), W*ushicha* Granule however has multiple functions of dispelling *wind* and relieving exterior syndrome, as well as resolving *damp* and regulating *stomach*. Thus, it is usually used to treat several *wind-cold*-induced gastroenterology disorders, such as ulcerative colitis, nausea, vomiting, abdominal pain, and diarrhea [2, 3].

These functions have facilitated it wide consumption in China. Nowadays, *Wushicha* Granule, as an over-the-counter-drug (OTC) prescription, can be accessed via online and pharmacy sales. According to the data from National Medical Products Administration of China [4], at least 52 pharmaceutical manufacturers, including Guangzhou *Wanglaoji* Pharmaceutical Co., Ltd, have been approved to manufacture the Granule.

The manufacture of *Wushicha* Granule is fulfilled by mixing 19 Chinese herbal medicines (CHMs) (Table 1). The manufacturing techniques are expected to comply with the Pharmacopoeia (Chinese Pharmacopoeia, 2020 version). However, in 2021, two batches of *Wushicha* Granule have been identified as unqualified products [5, 6]. This has attracted public attention regarding its quality-markers (Q-markers) in Pharmacopoeia.

**Table 1:** The formula of *Wushicha* Granule prescription (2600 g in total)

Chinese herbal medicine	Chinese name	Weight	Chinese herbal medicine	Chinese name	Weight
*Atractylodis rhizome*	Cangzhu, 蒼术	50 g	*Crataegi fructus*	Shanzha, 山楂	50 g
*Bupleurum chinense*	Chaihu, 柴胡	50 g	*Aurantii fructus immaturus*	Zhishi, 枳實	50 g
*Notopterygii rhizoma et radix*	Qianghuo, 羌活	50 g	*Hordei fructus germinatus*	Maiya, 麥芽	75 g
*Saposhnikoviae radix*	Fangfeng, 防風	50 g	*Glycyrrhizae radix et rhizoma*	Gancao, 甘草	50 g
*Angelicae dahuricae radix*	Baizhi, 白芷	50 g	*Platycodonis radix*	Jiegeng, 桔梗	75 g
*Chuanxiong rhizoma*	Chuanxiong, 川芎	50 g	*Perillae folium*	Zisuye, 紫蘇葉	75 g
*Pogostemonis herba*	Guanghuoxiang, 廣藿香	50 g	*Magnoliae officinalis cortex*	Houpo, 厚樸	75 g
*Peucedani radix*	Qianhu, 前胡	50 g	*Massa Medicata Fermentata*	Liushenqu, 六神曲	75 g
*Forsythiae fructus*	Lianqiao, 連翹	50 g	Black tea	Hongcha 紅茶	1600 g
*Citri reticulatae pericarpium*	Chenpi, 陳皮	50 g			

The current Pharmacopoeia only defines three Q-markers. One Q-marker hesperidin is for HPLC analysis; while another Q-markers phillyrin and “glycyrrhetinic acid” Q-markers however are for TLC (thinner layer chromatography) analysis [3]. In line with Table 1 and the accumulated literatures [3, 7–9], the current Q-markers system only involves four CHMs (i.e., Lianqiao, Chenpi, Zhishi, and Gancao). Some important CHMs, such as Hongcha, Chaihu, Chuanxiong, and Houpo, have not been involved [3]. As a result, the current system could not recognize the counterfeits concerning Hongcha, Chaihu, Chuanxiong, and Houpo. This is considered as the first limitation of current Pharmacopoeia Q-markers system.

In addition, the current Q-markers system has not yet discriminated two configurations of glycyrrhetinic acid, i.e., 18α- and 18β-. The former presents *18S-;* while the latter shows *18R-,* according to the updated nomenclature guideline. Thus, the two actually are a pair of stereo-isomers. Two stereo-isomers have been reported to possess different pharmacological effects. 18β-Glycyrrhetinic acid had hepatocyte protection effect; while 18α-glycyrrhetinic acid did not. On the other hand, 18α-glycyrrhetinic acid could selectively inhibit 11-hydroxysteroid dehydrogenase I, whilst 18β-glycyrrhetinic acid could not [10]. This situation is similar to two thalidomide stereo-isomers (i.e.,* R*- and *S*-thalidomides). Therefore, the confusion of 18α-, and 18β-glycyrrhetinic acids, may cause a tragedy similar to “Thalidomide Disaster” in 1960s. This can be regarded as the second limitation of current Pharmacopoeia Q-markers system.

Two limitations urge pharmacists to update the current Q-markers system, by means of an appropriate method, such as addition of new Q-marker. The update of course includes a fundamental work to discriminate 18α-glycyrrhetinic acid and 18β-glycyrrhetinic acid. All these obviously require a systematical and reliable identification for the main compounds in *Wushicha* Granule. Thereby, the study attempted to use a novel database-aided cutting-edge ultra-high performance liquid chromatography-quadrupole-orbitrap mass spectrometry (UHPLC-Q-orbitrap MS/MS) strategy, to fulfill the identification. Some identified compounds would further be recommended as Q-marker candidates for consideration by the Pharmacopoeia Commission.

## Materials and methods

### Wushicha Granule and its counterfeits

*Wushicha* Granule was purchased from Hubei Wushi Pharmaceutical Co., LTD (Anlu, Hubei, China). Its Lot No. was Z42020134, and production date was Jan. 13, 2022.

Six counterfeit *Wushicha* Granules were prepared by our team through replacement method. Both Zhishi and Chenpi were replaced by wood powder, to prepared the first counterfeit *Wushicha* Granule, i.e., CWG 1. Similarly, Chaihu was replaced by wood powder, to obtain CWG 2. In addition, Hongcha, Chuanxiong, Houpo, and Gancao were by wood powder, to produce CWG 3, CWG 4, CWG 5, and CWG 6, respectively (Table 2).

**Table 2:** The premixing of 6 counterfeit *Wushicha* Granules (CWG 1–CWG 6)

CWG 1	Cangzhu 5 g, Shanzha 5 g, Chaihu 5 g, ***Wood powder 10 g***, Qianghuo 5 g, Maiya 7.5 g, Fangfeng 5 g, Gancao 5 g, Baizhi 5 g, Jiegeng 7.5 g, Chuanxiong 5 g, Zisuye 7.5 g, Guanghuoxiang 5 g, Houpo 7.5 g, Qianhu 5 g, Liushenqu 7.5 g, Lianqiao 5 g, Hongcha 160 g
CWG 2	Cangzhu 5 g, Shanzha 5 g, ***Wood powder 5 g***, Zhishi 5 g, Qianghuo 5 g, Maiya 7.5 g, Fangfeng 5 g, Gancao 5 g, Baizhi 5 g, Jiegeng 7.5 g, Chuanxiong 5 g, Zisuye 7.5 g, Guanghuoxiang 5 g, Houpo 7.5 g, Qianhu 5 g, Liushenqu 7.5 g, Lianqiao 5 g, Chenpi 5 g, Hongcha 160 g
CWG 3	Cangzhu 5 g, Shanzha 5 g, Chaihu 5 g, Zhishi 5 g, Qianghuo 5 g, Maiya 7.5 g, Fangfeng 5 g, Gancao 5 g, Baizhi 5 g, Jiegeng 7.5 g, Chuanxiong 5 g, Zisuye 7.5 g, Guanghuoxiang 5 g, Houpo 7.5 g, Qianhu 5 g, Liushenqu 7.5 g, Lianqiao 5 g, Chenpi 5 g, ***Wood powder 160 g***
CWG 4	Cangzhu 5 g, Shanzha 5 g, Chaihu 5 g, Zhishi 5 g, Qianghuo 5 g, Maiya 7.5 g, Fangfeng 5 g, Gancao 5 g, Baizhi 5 g, Jiegeng 7.5 g, Chuanxiong 5 g, Zisuye 7.5 g, ***Wood powder 5 g***, Houpo 7.5 g, Qianhu 5 g, Liushenqu 7.5 g, Lianqiao 5 g, Chenpi 5 g, Hongcha 160 g
CWG 5	Cangzhu 5 g, Shanzha 5 g, Chaihu 5 g, Zhishi 5 g, Qianghuo 5 g, Maiya 7.5 g, Fangfeng 5 g, Gancao 5 g, Baizhi 5 g, Jiegeng 7.5 g, Chuanxiong 5 g, Zisuye 7.5 g, Guanghuoxiang 5 g, ***Wood powder 7.5 g***, Qianhu 5 g, Liushenqu 7.5 g, Lianqiao 5 g, Chenpi 5 g, Hongcha 160 g
CWG 6	Cangzhu 5 g, Shanzha 5 g, Chaihu 5 g, Zhishi 5 g, Qianghuo 5 g, Maiya 7.5 g, Fangfeng 5 g, ***Wood powder 5 g***, Baizhi 5 g, Jiegeng 7.5 g, Chuanxiong 5 g, Zisuye 7.5 g, Guanghuoxiang 5 g, Houpo 7.5 g, Qianhu 5 g, Liushenqu 7.5 g, Lianqiao 5 g, Chenpi 5 g, Hongcha 160 g

### Chemicals

Methyl gallate (C_8_H_8_O_5_, M.W. 192.16, Cas. 99–24-1, 98%), *S-*senkyunolide A (C_12_H_16_O_2_, M.W. 192.25, Cas. 63038-10-8, 98%), saikosaponin A (C_42_H_68_O_13_, M.W. 780.98, Cas. 20736-09-8, 98%), licoricesaponin H2 (C_42_H_62_O_16_, M.W. 822.9, Cas. 118441-85-3, 98%), quinic acid (C_7_H_12_O_6_, M.W. 192.16, Cas. 77–95-2, 98%), myricetin (Cas. 529–44-2, C_15_H_10_O_8_, M.W. 318.24, 97%), liquiritin (C_21_H_22_O_9_, M.W. 418.39, Cas. 551–15-5, 98%), scoparone (C_11_H_10_O_4_, M.W. 206.19, Cas. 120–08-1, 98%), platycodin D (C_57_H_92_O_28_, M.W. 1225.32, Cas. 58479-68-8, 98%), 18α-glycyrrhetinic acid (C_30_H_46_O_4_, M.W. 470.69, Cas. 1449–05-4, 98%), and 18β-glycyrrhetinic acid (C_30_H_46_O_4_, M.W. 470.69, Cas. 471–53-4, 98%) were obtained from Herbest Biotech Co., Ltd (Baoji, China). ( +)-4-Cholesten-3-one (Cas. 601–57-0, C_27_H_44_O, M.W. 384.64, 98%), ethyl stearate (Cas. 111–61-5, C_20_H_40_O_2_, M.W. 312.53, 98%), and 5-hydroxyflavone (Cas. 491–78-1, C_15_H_10_O_3_, M.W. 238.24, 97%) were from TCI Chemical Co. (Shanghai, China). D-gluconic acid (Cas. 526–95-4, C_6_H_12_O_7_, M.W. 196.155, 98%) was from Sigma-Aldrich Co., Ltd. (Shanghai, China). Randaiol (Cas. 87562-14-9, C_15_H_14_O_3_, M.W. 242.27, 97%), luteolin (Cas. 491–70-3, C_15_H_10_O_6_, M.W. 286.24, 97%), (-)-pinoresinol (Cas. 81446-29-9, C_20_H_22_O_6_, 358.39, 97%), isorhamnetin-3-*O*-β-D-glucoside (Cas. 5041–82-7, C_22_H_22_O_12_, M.W. 478.4, 97%), and acteoside(Cas. 61276-17-3, C_29_H_36_O_15_, M.W. 624.59, 97%) were from BioBioPha Co., Ltd. (Kunming, China). Hypericin (Cas. 548–04-9, C_30_H_16_O_8_, M.W. 504.45, 97%), *S-*hesperetin (Cas. 520–33-2, C_16_H_14_O_6_, M.W. 302.28, 97%), naringenin chalcone (Cas. 73692-50-9, M.W. C_15_H_12_O_5,_ 272.25, 97%), *S*-naringenin (Cas. 480–41-1, C_15_H_12_O_5_, M.W. 272.253, 98%), 7,4’-dihydroxyflavone (Cas. 2196–14-7, C_15_H_10_O_4_, M.W. 254.238, 98%), astragalin (Cas. 480-10-4, C_21_H_20_O_11_, M.W. 448.38, 97%), isochlorogenic acid A (Cas. 2450–53-5, C_25_H_24_O_12_, M.W. 516.45, 97%), rosmarinic acid (Cas. 20283-92-5, C_18_H_16_O_8_, M.W. 360.31, 97%), naringin (Cas. 10236-47-2, C_27_H_32_O_14_, M.W. 580.53, 97%), rutin (Cas. 153–18-4, C_27_H_30_O_16_, M.W. 610.518, 98%), isoquercitrin (Cas. 21637-25-2, C_21_H_20_O_12_, 464.38, 97%), isovitexin (Cas. 38953-85-4, C_21_H_20_O_10_, M.W. 432.37, 98%), isoliquiritin (Cas. 5041–81-6, C_21_H_22_O_9_, M.W. 418.39, 97%), and puerarin (Cas. 3681-99-0, C_21_H_20_O_10_, M.W. 416.38, 97%) were from Chengdu Alfa Biostrategy Co., Ltd. (Chengdu, China). Formononetin (Cas. 485–72-3, C_16_H_12_O_4_, M.W. 268.264, 98%), isoliquiritigenin (Cas. 961-29-5, C_15_H_12_O_4_, M.W. 256.253, 98%), daidzein (Cas. 486-66-8, C_15_H_10_O_4_, M.W. 254.24, 97%), naringenin-7-*O*-β-D-glucoside (Cas. 529-55-5, C _21_H_22_O_10_, M.W. 434.393, 98%), 5-caffeoylquinic acid (Cas. 906-33-2, C_16_H_18_O_9_, M.W. 354.31, 98%), and gallic acid (Cas. 149–91-7, C_7_H_6_O_5_, M.W. 170.1, 99%) were from Chengdu Biopurify Phytochemicals Ltd. (Chengdu, China).Magnolol (Cas. 528–43-8, C_18_H_18_O_2_, M.W. 266.32, 97%), 3,3',4',5,6,7,8-heptamethoxyflavone (Cas. 1178-24-1, C_16_H_12_O_3_, M.W. 252.26, 97%), quercetin(Cas. 117–39-5, C_15_H_10_O_7_, M.W. 302.23, 97%), vitexin (Cas. 3681-93-4, C_21_H_10_O_10_, M.W. 432.11, 97%), schaftoside (Cas. 51938-32-0, C_26_H_28_O_14_, M.W. 564.49, 98%), vicenin-2 (Cas. 23666-13-9, C_27_H_30_O_15_, M.W. 594.518, 98%), and protocatechuic acid (Cas. 99-50-3, C_7_H_6_O_4_, M.W. 154.12, 97%) were from Sichuan Weikeqi Biological TechnologyCo., Ltd. (Chengdu, China). Caffeine (Cas. 58-08-2, C_8_H_10_N_4_O_2_, M.W. 194.19, 98%) was prepared by our laboratory. Methanol, and water were of mass spectra purity grade. All other reagents used in this study were purchased as analytical grade from the Guangzhou Chemical Reagent Factory (Guangzhou, China).

### The preparation of sample solution

The purchased *Wushicha* Granule was dissolved using distilled water under ultrasound treatment, to avoid the possible solvent effect [11]. The dissolution however brought about a turbid liquid. The turbid liquid was then filtered through a 0.45 μm membrane, to prepare a filtrate. The filtrate (at 30 mg/mL) was then kept in cell-bottle at 2–6 °C for analysis [12].

Furthermore, 6 premixed counterfeit Granules (i.e., CWG 1 ~ CWG 6) were prepared for their lyophilized aqueous extract powders, in line with Jiang’s method [13]. Then, 6 lyophilized powders were dissolved using distilled water under ultrasound treatment at 30 mg/mL and filtered through a 0.45 μm membrane to prepare a filtrate, respectively. All filtrates were then kept in cell-bottle at 2–6 °C for analysis [12].

### Database establishment and UHPLC-Q-orbitrap MS analysis

The database has been built up using the corresponding authentic standards, according to the previous study [14]. In brief, these authentic standards were dissolved in methanol at 30 μg/mL, respectively. The methanolic solution was then filtered through a 0.45 μm membrane, and kept in cell-bottle at 2–6 °C for analysis. The ultrahigh-performance liquid chromatography (UHPLC) was conducted, according to the previous method [15, 16]. In brief, the UHPLC separation was achieved by the mobile phase comprising 0.1% HCOOH (phase A) and methanol (phase B). The binary gradient was set as following: 0–5 min, 10% B; 5–14.5 min, 10 → 100% B; 14.5–16 min, 100% B; 16–16.1 min, 100 → 0% B. Then, the 10% B mobile phase was kept for 4 min to equilibrate the system. The mobile phase run at a flow rate of 0.4 mL min 1. The column temperature was maintained at 40 °C and injection volume was 3 μL.

The Q-orbitrap MS analysis was performed a high-resolution Q-orbitrap mass spectrometer (Thermo Fisher Scientific, Waltham, MA, USA) and consulted with our previous instrument settings [17, 18]. The operating parameters were detailed as follows: auxiliary gas, 10; sheath gas, 40; sweep gas, 0; spray voltage, 4.5 kV. The temperature of auxiliary gas heater and capillary were both set at 450 °C. The full MS resolution and dd-MS^2^ were 70,000 and 17,500, respectively, and their AGC target was 2 × 10^5^. Nitrogen (N_2_) was applied for spray stabilization and the damping gas in the C- trap. The stepped normalized collision energy was set to 20, 50, and 90 V. The MS scanning scope was set as *m/z* 0–1500. The analyses were conducted under both negative and positive models. The positive model however was a supplement of the negative one. The UHPLC-Q-orbitrap MS analysis only focused on the authentic standards and *Wushicha* Granule (except for 6 counterfeits).

### Putative identification using software and MS spectra elucidation

Xcalibur 4.1 software package and TraceFinder General Quan (Thermo Fisher Scientific Inc., Waltham, MA, USA) were used for data acquisition and analysis [14]. The acquired data included retention time (R.T.), molecular peak, MS/MS profile, and diagnostic MS/MS fragments of authentic standards. The acquisition was achieved based on the previous conditions [19]. Through the comparison with the database, 63 compounds were preliminarily identified from the *Wushicha* sample solution. After manual elucidation of MS spectra fragmenting, 52 compounds were further confirmed to finish putative identification.

### Quantum chemical calculation details

Seven compounds were investigated for quantum chemical calculation, including caffeine, hesperidin, saikosaponin A, *S*-senkyunolide A, 18α-glycyrrhetinic acid, 18β-glycyrrhetinic acid, and magnolol. All calculations were accomplished using the Gaussian 16 in Linux system, including conformational optimization, dipole moment calculation, and highest occupied molecular orbital (HOMO)–lowest unoccupied molecular orbital (LUMO) energy gap. The basis set was at (U)B3LYP-D3(BJ)/ 6–31 + G(d,p) level [20–22]. The most stable conformation was optimized until no imaginary frequency; while the calculation results (including optimized conformation) were viewed via Gaussian View 6.1.1 [23]. The optimized conformation was further exported using Chem3D pro. 14.0. Gaussian 16, and Gaussian View 6.1.1 (Gaussian Inc., Wallingford, CT, USA).

### UV–vis spectra scanning experiments

The UV–vis spectra scanning experiments were conducted based on the previous method [24]. In brief, 6 compounds were dissolved in methanol at appropriate concentrations, respectively. Their methanolic solutions were then scanned using a UV–vis spectrophotometer (Unico 2600A, Shanghai, China) from 200 to 800 nm, respectively. Six compounds referred to hesperidin, phillyrin, magnolol, caffeine, *S-*senkyunolide A, 18β-glycyrrhetinic acid, and saikosaponin A. All these compounds were at ~ 0.02 mg/mL concentration.

### Anti-counterfeiting validation experiment based on 6 counterfeits

The *anti*-counterfeiting validation experiment was performed using ultra-performance liquid chromatography coupled with electrospray ionization quadrupole time-of-flight tandem mass spectrometry (UHPLC-ESI-Q-TOF–MS/MS), an analytic technology inferior to UHPLC-Q-orbitrap MS/MS. The chromatography analysis protocol was based on our previous studies [25, 26]; while the MS spectra monitoring was achieved using a Q − TOF − MS/MS apparatus (i.e., Triple TOF 5600^*plus*^ mass spectrometer, AB SCIEX, Framingham, MA, U.S.A.) [27]. However, the analytes were 6 counterfeit *Wushicha* Granules, i.e., CWG 1–CWG 6. For comparison, the *Wushicha* sample solution was also prepared for this analysis, under the same conditions.

## Results

### Putative identification based on UHPLC-Q-orbitrap MS/MS

**Fig. 2:**
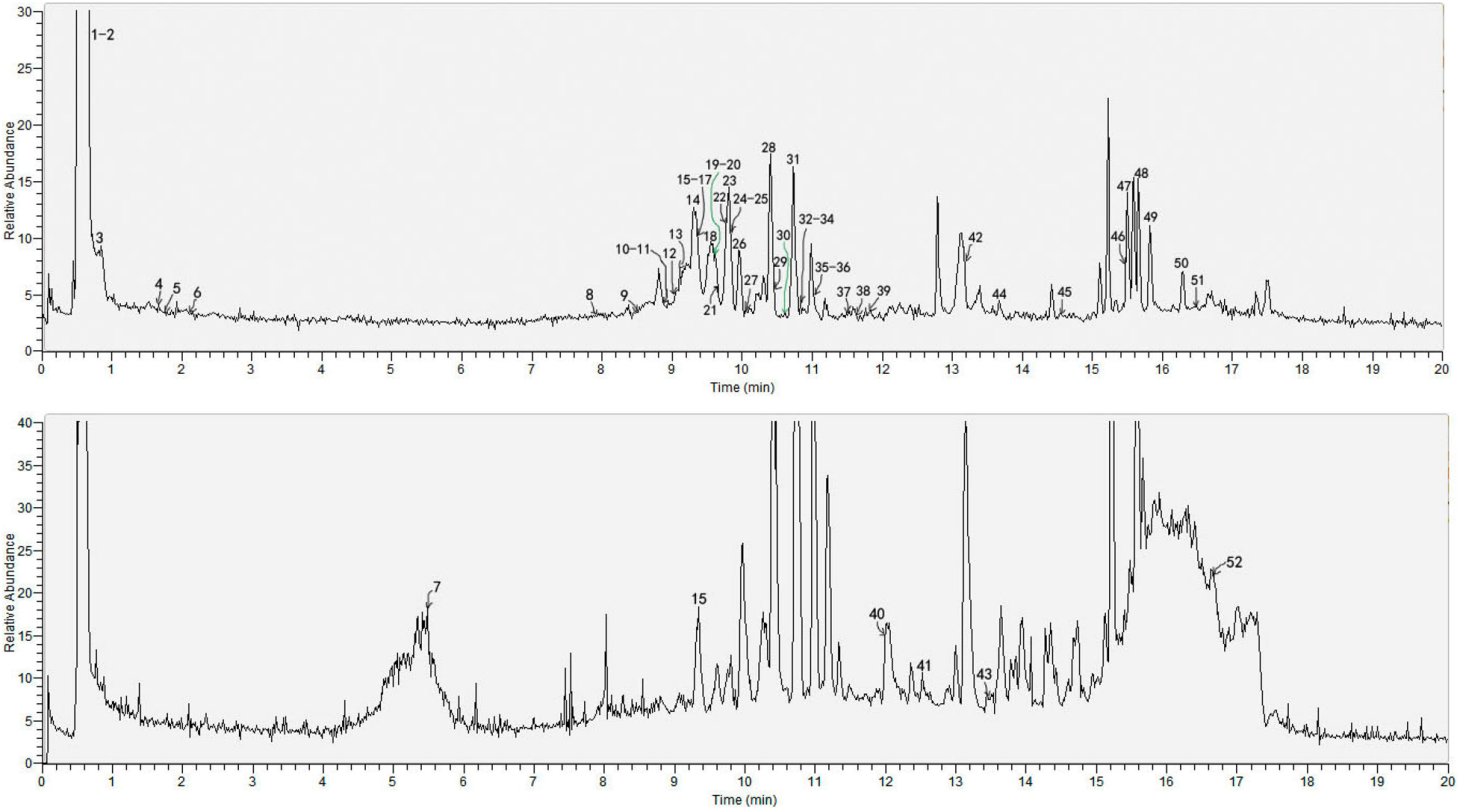
The total ion current (TIC) chromatogram of *Wushicha* Granule by the database-aided UHPLC-Q-orbitrap MS/MS analysis (Upper for negative ion mode; below for positive model. The positive mode however was the supplement for negative mode)

**Fig. 3:**
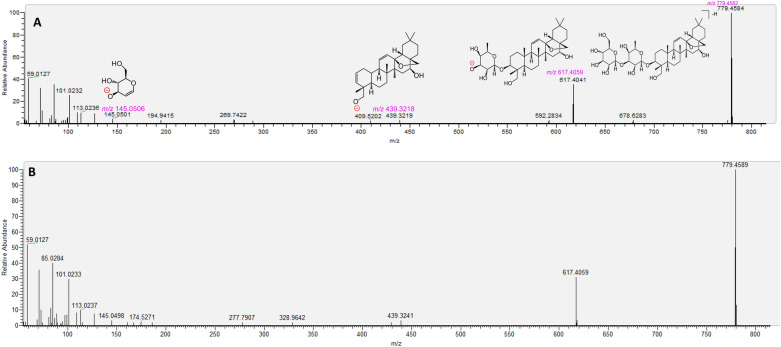
The putative identification of saikosaponin A (**28**) based on MS spectra under negative model. **A** MS/MS spectra and the relevant elucidation of standard saikosaponin A; **B** MS/MS spectra of the sample peak from *Wushicha* Granule. The *m/z* values in purple indicated the calculated ones. The detailed MS elucidation, and identification process were shown in in Additional file 27: S27

**Fig. 4:**
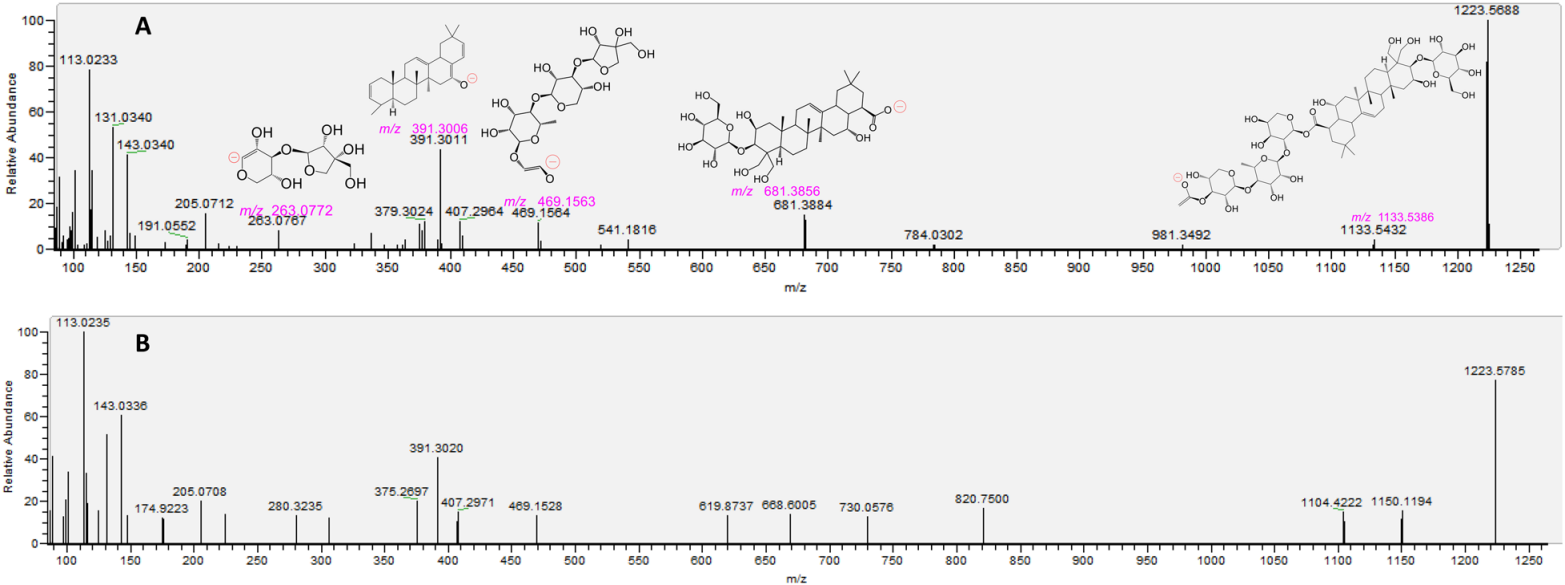
The putative identification of platycodin D (**38**) based on MS spectra under negative model. **A** MS/MS spectra and the relevant elucidation of standard platycodin D; **B** MS/MS spectra of the sample peak from *Wushicha* Granule. The *m/z* values in purple indicated the calculated ones. The detailed MS elucidation, and identification process were shown in Additional file 37: S37

**Fig. 5:**
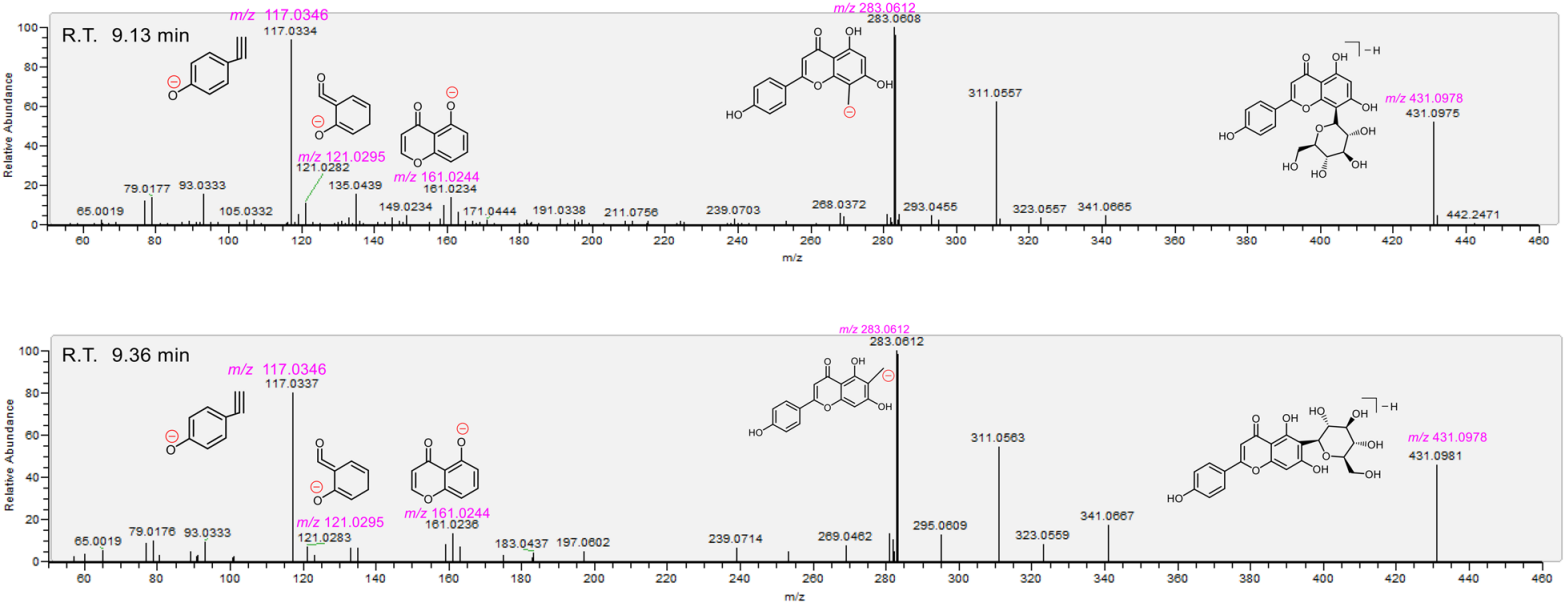
The distinction of vitexin (**13**, upper) and isovitexin (**17**, below) based on MS spectra under negative model. The *m/z* values in purple indicated the calculated ones. The detailed MS elucidation, and identification process were shown in in Additional file 13: S13 and Additional file 17: S17

**Fig. 6:**
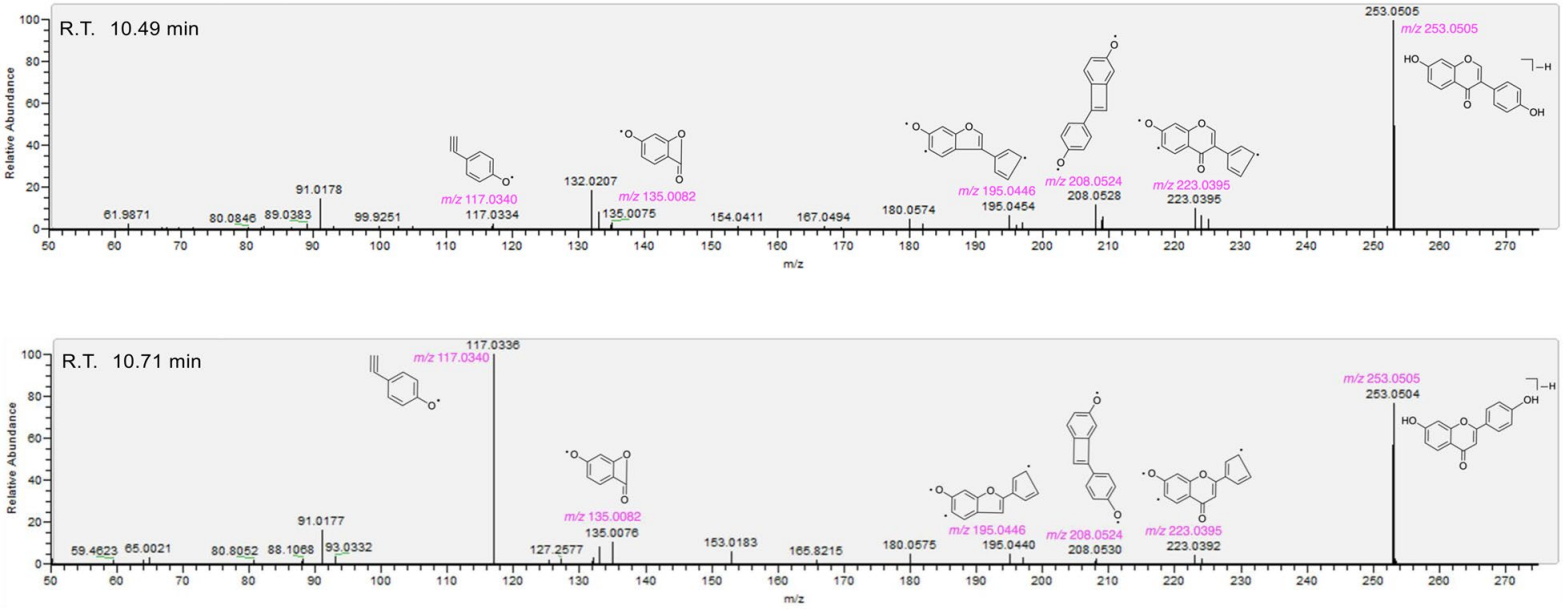
The MS/MS spectra, and the relevant elucidation of daidzein (**29,** upper) and 7,4'-dihydroxyflavone (**31,** below). The *m/z* values in purple indicated the calculated ones. The detailed MS elucidation, and identification process were shown in Additional file 28: S28 and Additional file 30: S30

**Fig. 7:**
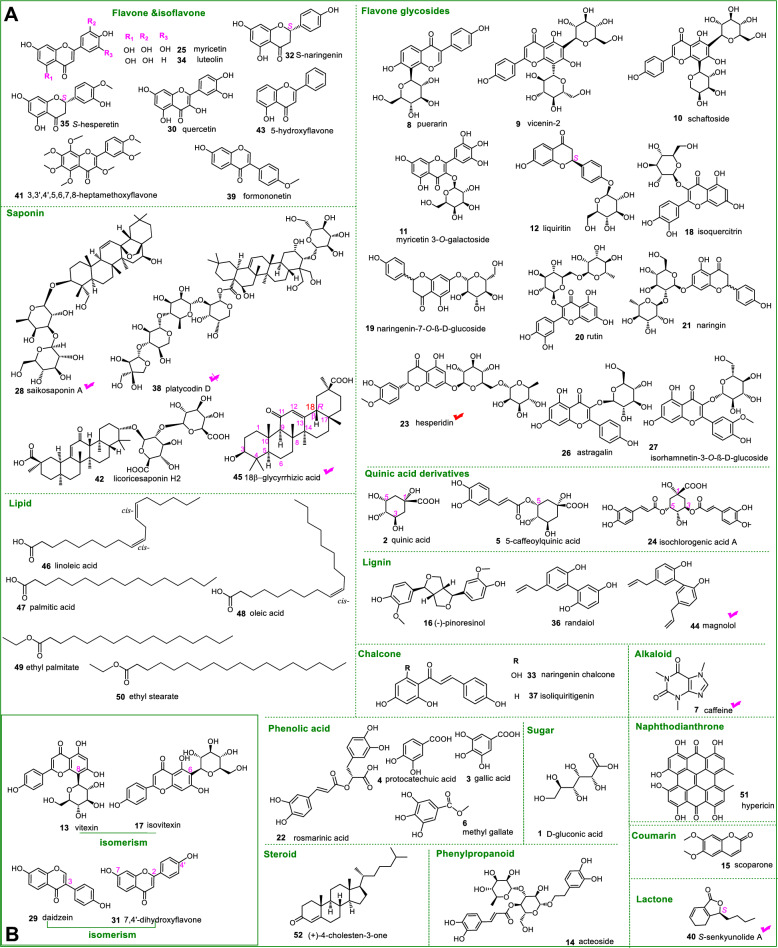
The structures of 52 identified compounds from *Wushicha* Granule by database-aided UHPLC-Q-orbitrap MS/MS strategy. **A** 48 non-isomeric compounds; **B** two pairs of isomeric compounds. The red “√” indicates the old Pharmacopoeia Q-markers; while the purple “√” means the new Q-marker candidates. The wave lines in **18** and **21** indicate the uncertain stereo-configuration

The *Wushicha* sample solution was firstly analyzed using UHPLC-Q-orbitrap MS/MS strategy. Through analysis, the total ion current (TIC) diagrams were obtained (Fig. 2); while the main ion peaks were further investigated for the R.T. values, molecular ion peak, and diagnostic MS/MS fragments (Table 3). These information was compared with that of authentic standards in the database; and thereafter the MS spectra of all compounds were fully elucidated, according to relevant principles (e.g., *Retro*-Diels–Alder fragmenting, Additional file 1: S1, Additional file 2: S2, Additional file 3: S3, Additional file 4: S4, Additional file 5: S5, Additional file 6: S6, Additional file 7: S7, Additional file 8: S8, Additional file 9: S9, Additional file 10: S10, Additional file 11: S11, Additional file 12: S12, Additional file 13: 13, Additional file 14: S14, Additional file 15: S15, Additional file 16: S16, Additional file 17: S17, Additional file 18: S18, Additional file 19: S19, Additional file 20: S20, Additional file 21: S21, Additional file 22: S22, Additional file 23: S23, Additional file 24: S24, Additional file 25: S25, Additional file 26: S26, Additional file 27: S27, Additional file 28: S28, Additional file 29: S29, Additional file 30: S30, Additional file 31: S31, Additional file 32: S32, Additional file 33: S33, Additional file 344: S34, Additional file 35: S35, Additional file 36: S36, Additional file 37: S37, Additional file 38: S38, Additional file 39: S39, Additional file 40: S40, Additional file 41: S41, Additional file 42: S42, Additional file 43: S43, Additional file 44: S44, Additional file 45: S45, Additional file 46: S46, Additional file 47: S47, Additional file 48: S48, Additional file 49: S49, Additional file 50: S50, Additional file 51: S51). In particular, 6 compounds, including saikosaponin A, platycodin D, vitexin, isovitexin, daidzein*,* and 7,4'-dihydroxyflavone, were also shown their MS spectra elucidation in Figs. 3, 4, 5, 6. Finally, the structures (and even configurations) of all identified compounds were detailed in Fig. 7, for the convenience of readers.

**Table 3:** The main experimental results of 52 putatively identified compounds (**1–52**)

No	RT min	Name	Molecular ion	Observed *m/z* value	Theoretical *m/z* value	Error (δ ppm)	Molecular ion peak and fragment peak *m/z*	Plant source
1	0.53	D-gluconic acid	C_6_H_11_O_7_^−^	195.0501	195.0505	2.0508	**195.0504, 177.0397, 159.0292, 129.0184,** 99.0077, 87.0076	Liushenqu
2	0.54	Quinic acid	C_7_H_11_O_6_^−^	191.0554	191.0556	1.0468	**191.0554, 173.0449, 127.0387**, 109.0286, 93.0334, 85.0283	Shanzha [28], Hongcha [29]
3	0.81	Gallic acid	C_7_H_5_O_5_^−^	169.0131	169.0137	3.5500	**169.0134, 125.0234,** 124.0156**, 107.0127,** 97.0284**,** 79.0178	Lianqiao [9, 30], hongcha [31]
4	1.65	Protocatechuic acid	C_7_H_5_O_4_^−^	153.0182	153.0188	3.9211	**153.0184**, 110.0316, **109.0284**, **108.0206**, 91.0177, 81.0334	Chuanxiong [32], Chaihu [33], Lianqiao[9]
5	1.73	5-caffeoylquinic acid	C_16_H_17_O_9_^−^	353.0872	353.0873	0.2832	**353.0866**, **191.0554**, **179.0340, 173.0449**, 161.0239, **135.0442**, 107.0489, 93.0334, 85.0283,	Chaihu [34]
6	2.14	Methyl gallate	C_8_H_7_O_5_^−^	183.0291	183.0293	1.0927	**183.0291, 168.0056,** 140.0110**, 139.0389, 124.0156**, 95.0128, 89.9247	Hongcha [35]
7^a^	5.46	Caffeine	C_8_H_11_N_4_O_2_^+^	195.0874	195.0882	4.1007	**195.0870, 138.0658, 123.0425, 110.0713**, 108.0552, 83.0607, 69.0453, 56.0502	Hongcha [31]
8	7.92	Puerarin	C_21_H_19_O_9_^−^	415.1028	415.1029	0.2409	**415.1035, 295.0614**, 277.0500**, 267.0663, 253.0504**, 222.0680, 209.0604, **132.0208,** 105.0334	Gancao [36], Maiya [37]
9	8.43	Vicenin-2	C_27_H_29_O_15_^−^	593.1500	593.1506	1.0115	**593.1511,** 473.1089, 383.0774, 353.0669, **325.0730**, **297.0768**, **283.0605**, 117.0336	Hongcha [31]
10	8.86	Schaftoside	C_26_H_27_O_14_^−^	563.1395	563.1401	1.0655	**563.1405**, **383.0769**, **353.0668**, 325.0721, **297.0767**, 135.0443, **117.0336,** 93.0334, 79.0178	Chaihu [38]
11	8.87	Myricetin-3-*O*-galactoside	C_21_H_19_O_13_^−^	479.0821	479.0826	1.0437	**479.0829**, **316.0221**, **287.0189**, **271.0249**, 259.0247, **242.0268**, 151.0028, 124.0155	Hongcha [39]
12	9.05	Liquiritin	C_21_H_21_O_9_^−^	417.1185	417.1186	0.2397	**417.1195**, **255.0661**, 153.0185, **135.0078**, **119.0492**, 108. 0206, 91.0178	Chaihu [40], Gancao [41]
13	9.13	Vitexin	C_21_H_19_O_10_^−^	431.0975	431.0978	0.6959	**431.0986**, **341.0061**, **311.0561**, **283.0613**, 268.0373, **135.0443,** 117.0336	Hongcha [17], Shanzha [28]
14	9.30	Acteoside	C_29_H_35_O_15_^−^	623.1967	623.1976	1.4442	**623.1977**, **461.1666**, 179.0341, **161.0236**, 143.0339, **133.0285**,115.0179	Houpo [42], Lianqiao [9]
15^a^	9.35	Scoparone	C_11_H_11_O_4_^+^	207.0649	207.0652	1.4488	**207.0646, 191.0331, 163.0386, 151.0750**, **146.0359**, 135.0437, 107.0492	Chuanxiong [30]
16	9.36	(-)-Pinoresinol	C_20_H_21_O_6_^−^	357.1336	357.1338	0.5600	**357.1339**, 342.1114, **151.0392**, **136.0156, 121.0285**, 108.0207,	Lianqiao [43]
17	9.39	Isovitexin	C_21_H_19_O_10_^−^	431.0976	431.0978	0.4639	**431.0981, 341.0667**, 323.0559**, 311.0556, 283.0612**, 269.0462, 117.0337	Hongcha[31]
18	9.51	Isoquercitrin	C_21_H_19_O_12_^−^	463.0873	463.0877	0.8638	**463.0884, 300.0270, 271.0244, 255.0293,** 243.0291, 227.0341,199.0390	Shanzha [44]
19	9.55	Naringenin-7-*O*-β-D-glucoside	C_21_H_21_O_10_^−^	433.1133	433.1135	0.4618	**433.1685, 271.0615**, 177.0187, **151.0029, 119.0493**, 107.0128, 93.0335	Shanzha [45]
20	9.56	Rutin	C_27_H_19_O_16_^−^	609.1445	609.1456	1.8058	**609.1445, 300.0277, 271.0249, 255.0297, 243.0297**, 199.0393, 171.0443, 151.0026	Houpo [42], Chuanxiong [30], Lianqiao [30], Liushenqu [46]
21	9.65	Naringin	C_27_H_31_O_14_^−^	579.1708	579.1714	1.0360	**579.1730**, 459.1143, **271.0611,** 227.0694**, 151.0028, 119.0492, 107.0127**	Zhishi[47], Chenpi[48]
22	9.76	Rosmarinic acid	C_18_H_15_O_8_^−^	359.0764	359.0767	0.8355	**359.0780**, **197.0450**, 179.0341, **161.0235**, 135.0442, **133.0285**, 123.0442	Zisuye [49],
23	9.79	Hesperidin	C_28_H_33_O_15_^−^	609.1816	609.1819	0.4925	609.1816, **301.0717**, 286.0483, 242.0582, 199.0396, **164.0107**, **108.0207**	Zhishi [47], Chenpi [50],
24	9.84	Isochlorogenic acid A	C_25_H_23_O_12_^−^	515.1180	515.1190	1.9413	**515.1226, 353.0878, 179.0342,** 173.0447, **135.0441**, 93.0335	Qianghuo [51]
25	9.84	Myricetin	C_15_H_9_O_8_^−^	317.0294	317.0297	0.9463	**317.0304**, 178.9979, **151.0029, 137.0235**, 119.0134, **109.0284**	Hongcha [31]
26	10.00	Astragalin	C_21_H_19_O_11_^−^	447.0924	447.0927	0.6710	**447.0934**, 327.0503, **284.0330**, **255.0298, 227.0346**, 211.0395, 199.0397, 183.0444	Chuanxiong [52]
27	10.05	Isorhamnetin-3*-O*-β-D-glucoside	C_22_H_21_O_12_^−^	477.1032	477.1033	0.2096	**477.1034**, 314.0420, 299.0193, **285.0401**, 257.0452, **271.0253, 243.0295**, 215.0343	Houpo [42]
28	10.38	Saikosaponin A	C_42_H_67_O_13_^−^	779.4584	779.4582	0.2566	**295.0605**, **185.0234**, 159.0446, 135.0442, 123.0438,** 109.0285**	Chaihu [33, 53]
29	10.49	Daidzein	C_15_H_9_O_4_^−^	253.0501	253.0501	0.0000	**253.0505**, **223.0395, 208.0528, 195.0454**, 180.0574, 132.0207, 91.0178	Zhishi [47]
30	10.62	Quercetin	C_15_H_9_O_7_^−^	301.0348	301.0348	0.0000	**301.0353,** 178.9978, **151.0028**, 149.0234, **121.0285, 107.0128**	Chaihu [33], Hongcha [31], Liushenqu [46]
31	10.71	7,4'-dihydroxyflavone	C_15_H_9_O_4_^−^	253.0501	253.0501	0.0000	**253.0504, 223.0392**, **208.0530,** 153.0183, **135.0076**, **117.0336**, 91.0177	Gancao [41]
32	10.87	*S-*naringenin	C_15_H_11_O_5_^−^	271.0609	271.0606	1.1068	**271.0613,** 177.0182, 165.0185,** 151.0027, 119.0492, 107.0128**	Zhishi [47], Chenpi [50]
33	10.88	Naringenin chalcone	C_15_H_11_O_5_^−^	271.0608	271.0606	0.7378	**271.0613, 187.0394**, **151.0028**, **119.0492**, 107.0128, 93.033	Chenpi [50]
34	10.89	Luteolin	C_15_H_9_O_6_^−^	285.0401	285.0399	0.7017	**285.0405**, **257.0444, 217.0504**, 199.0394, 175.0393, 151.0027, **133.0285**	Shanzha [28, 54]
35	11.08	*S*-hesperetin	C_16_H_13_O_6_^−^	301.0713	301.0712	0.3321	**301.0715**, **164.0104,** 151.0028, 136.0156, **108.0206,** 80.0255	Zhishi [47], Qianghuo [55]
36	11.09	Randaiol	C_15_H_13_O_3_^−^	241.0863	241.0865	0.8296	**241.0867, 223.0761, 197.0965**, 157.0652, 141.0700, 95.0126	Houpo [42]
37	11.55	Isoliquiritigenin	C_15_H_11_O_4_^−^	255.0656	255.0657	0.3921	**255.0603**, 153.0181, **135.0073**, **119.0493**, 91.0177	Chaihu [38], Gancao [41]
38	11.63	Platycodin D	C_57_H_91_O_28_^−^	1223.5701	1223.5697	0.3269	**1223.5709,** 407.2975, **391.3025**, 143.0335, 131.0340, 113.0223	Jiegeng [56]
39	11.79	Formononetin	C_16_H_11_O_4_^−^	267.0659	267.0657	0.7489	**267.0664, 252.0427, 223.0396**, 208.0523, 195.0446, **167.0494,132.0206**	Chaihu [57, 58]
40^a^	12.00	*S*-senkyunolide A	C_12_H_17_O_2_^+^	193.1220	193.1229	4.6602	**193.0491**, 175.1111, **147.1164, 137.0593, 105.069**9, 95.0494, 91.0545	Chuanxiong [32],
41	12.49	3,3',4',5,6,7,8-heptamethoxyflavone	C_22_H_23_O_9_^−^	433.1486	433.1499	3.0013	**433.1479**, 418.1250, **403.1008**, **345.0589**, 205.0860, **165.0542,** 127.0388	Chenpi [59]
42	13.20	Licoricesaponin H2	C_42_H_61_O_16_^−^	821.3970	821.3960	1.2174	**821.3956**, **193.0344, 113.0233**, 85.0284	Gancao[30]
43	13.26	5-hydroxyflavone	C_15_H_9_O_3_^−^	239.0699	239.0708	3.7646	**239.0694, 165.0694**, 155.0336, **137.0229**, 129.0332, **103.0543**	Zhisuzi* [60]
44	13.68	Magnolol	C_18_H_17_O_2_^−^	265.1230	265.1229	0.3772	**265.1234**, **247.1125**, 245.0972, 243.0811, 223.0767	Houpo
45	14.54	18β-glycyrrhetinic acid	C_30_H_45_O_4_^−^	469.3322	469.3318	0.8523	**469.3322**, **425.3415**, 409.3105, **355.2628**	Gancao [36, 61]
46	15.42	Linoleic acid	C_18_H_31_O_2_^−^	279.2325	279.2324	0.3581	**279.2330, 261.2221**, 140.6269, 111.0682, 96.9586, 83.0491, 62.8557	Baizhi [62], Chaihu [33], Qianghuo [63], Chuanxiong [52]
47	15.54	Palmitic acid	C_16_H_31_O_2_^−^	255.2326	255.2324	0.7836	**255.2329**, 247.5200, 135.6278, 87.0019, 69.3279, 61.1978	Houpo[42], Chuanxiong [52], Chaihu [33], Zhisuzi [60], Shanzha[28]
48	15.63	Oleic acid	C_18_H_33_O_2_^−^	281.2487	281.2486	0.3556	**281.2489,** 201.4027, 186.0976, 112.4930, **92.1643**, 59.9085	Zhisuzi [60], Chaihu [33], Hongcha[31], Baizhi [62], Lianqiao [9]
49	15.81	Ethyl palmitate	C_18_H_35_O_2_^−^	283.2641	283.2637	1.4121	**283.2642**, 265.2533, 186.1121, 149.0329, 125.7779, 92.1631	Houpo [42], Hongcha [31]
50	16.35	Ethyl stearate	C_20_H_39_O_2_^−^	311.1683	311.2950	407.0094	**311.1689, 183.0113**, 133.0650, **119.0492**, 79.9561	Houpo [42]
51	16.50	Hypericin	C_30_H_15_O_8_^−^	503.0770	503.0767	0.5963	**503.0771**, 459.0873, 433.0717, 405.0772	Lianqiao [64]
52^a^	16.70	( +)-4-cholesten-3-one	C_27_H_45_O ^+^	385.3456	385.3470	3.6331	**385.3451, 367.3355, 255.2094**, 123.0804, **109.0650**, 97.0651	Liushenqu [65]

The original MS spectra, and identification process were detailed in Additional file 1: S1, Additional file 2: S2, Additional file 3: S3, Additional file 4: S4, Additional file 5: S5, Additional file 6: S6, Additional file 7: S7, Additional file 8: S8, Additional file 9: S9, Additional file 10: S10, Additional file 11: S11, Additional file 12: S12, Additional file 13: 13, Additional file 14: S14, Additional file 15: S15, Additional file 16: S16, Additional file 17: S17, Additional file 18: S18, Additional file 19: S19, Additional file 20: S20, Additional file 21: S21, Additional file 22: S22, Additional file 23: S23, Additional file 24: S24, Additional file 25: S25, Additional file 26: S26, Additional file 27: S27, Additional file 28: S28, Additional file 29: S29, Additional file 30: S30, Additional file 31: S31, Additional file 32: S32, Additional file 33: S33, Additional file 344: S34, Additional file 35: S35, Additional file 36: S36, Additional file 37: S37, Additional file 38: S38, Additional file 39: S39, Additional file 40: S40, Additional file 41: S41, Additional file 42: S42, Additional file 43: S43, Additional file 44: S44, Additional file 45: S45, Additional file 46: S46, Additional file 47: S47, Additional file 48: S48, Additional file 49: S49, Additional file 50: S50, Additional file 51: S51. The *m/z* value in bold means the diagnostic fragments. The *m/z* values below 50 were also found by the Xcalibur 4.1 Software package, despite that the scan mode rang was set at *m/z* 100–1500 in the mass spectra. The *m/z* values in square bracket means the molecular ion peak. Under the same conditions, 18α-glycyrrhetinic acid was excluded through the comparison with the authentic standard (R.T.) and main diagnostic fragments of *m/z* 355 (Additional file 44: S44). *, The previous work may confuse 5-hydroxyflavone 3-hydroxyflavone. Four compounds (**7, 15, 40, 52**) with “a” symbol were identified under positive ion model. All other compounds were identified under negative ion model

### Quantum chemical calculation

The main calculation results of 7 compounds were shown in Table 4**.** Seven compounds referred to hesperidin (**23**), 18α-glycyrrhetinic acid, 18β-glycyrrhetinic acid (**45**), caffeine (**7**), saikosaponin A (**28**), *S-*senkyunolide A (**40**), and magnolol (**44**).

**Table 4:** The main calculation results of 7 compounds at (U)B3LYP-D3(BJ)/ 6–31 + G(d,p) level

	Compound	Dipole moment	HOMO–LUMO	Remark
23	hesperidin	− 5.8110	0.15293	Old Q-markers
–	18α-glycyrrhetinic acid	4.6173	0.18229	For comparison
45	18β-glycyrrhetinic acid	3.1347	0.18158	Revised Q-marker
7	Caffeine	3.7942	0.18801	New Q-marker
28	Saikosaponin A	4.3637	0.22096	
40	*S-*senkyunolide A	5.8740	0.17358	
44	Magnolol	1.666	0.19164	

The conformational optimization was the basis of other calculations. The conformational optimization results were not shown to reduce the layout. The unit of dipole moment value was Debye; HOMO–LUMO, the energy gap from highest occupied molecular orbital to lowest unoccupied molecular orbital, a.u. unit, 1 a.u. = 2625.5 kJ/mol. Phillyrin has been used to characterize Liangqiao by TLC method in the Pharmacopoeia; however, it was not detected out in the study for its weak ionization potential

### UV–vis spectra scanning

### Anti-counterfeiting validation experiment using 6 counterfeit Wushicha Granules

## Discussion

The study established a novel strategy, i.e., database-aided UHPLC-Q-orbitrap MS/MS, to simultaneously identify 52 compounds from *Wushicha* Granule. Compared with the conventional HPLC–UV strategy which could simultaneously identify 2–10 compounds [66–70], our strategy was undoubtedly of high-efficiency. Besides high-efficiency, our strategy was of high-reliability as well. As seen in Figs. 3, 4, 5, 6 and Additional file 1: S1, Additional file 2: S2, Additional file 3: S3, Additional file 4: S4, Additional file 5: S5, Additional file 6: S6, Additional file 7: S7, Additional file 8: S8, Additional file 9: S9, Additional file 10: S10, Additional file 11: S11, Additional file 12: S12, Additional file 13: 13, Additional file 14: S14, Additional file 15: S15, Additional file 16: S16, Additional file 17: S17, Additional file 18: S18, Additional file 19: S19, Additional file 20: S20, Additional file 21: S21, Additional file 22: S22, Additional file 23: S23, Additional file 24: S24, Additional file 25: S25, Additional file 26: S26, Additional file 27: S27, Additional file 28: S28, Additional file 29: S29, Additional file 30: S30, Additional file 31: S31, Additional file 32: S32, Additional file 33: S33, Additional file 344: S34, Additional file 35: S35, Additional file 36: S36, Additional file 37: S37, Additional file 38: S38, Additional file 39: S39, Additional file 40: S40, Additional file 41: S41, Additional file 42: S42, Additional file 43: S43, Additional file 44: S44, Additional file 45: S45, Additional file 46: S46, Additional file 47: S47, Additional file 48: S48, Additional file 49: S49, Additional file 50: S50, Additional file 51: S51, our strategy was carried out via multiple comparisons including molecular ion peak comparison, diagnostic MS/MS peak comparison, MS/MS profile comparison, and R.T. value comparison. All these comparisons were based on authentic standards in the database. Therefore, the identification was highly-convincing and thus described as “putative identification” in the study.

One of putative identification instances was saikosaponin A (**30**). As seen in Fig. 3, the sample peak was highly similar to that of authentic standard, especially in molecular ion peak, diagnostic MS/MS peak, and MS/MS profile. Further MS elucidation suggested 10^–5^ ~ 10^–6^ relative standard deviation (RSD) values between the experimental and calculated *m/z* values. Such high-accuracy could also be observed in the identification of platycodin D (**38**), a non-isomeric compound (Fig. 4).

Our strategy however could also be used to discriminate isomers, e.g., two vitexin isomers (**13** and **17**). The two showed similar molecular ion peaks, diagnostic MS/MS fragments, and even MS/MS profiles. However, their R.T. values were different from each other (Fig. 5). The two were thus successfully discriminated using the strategy [18]. Similarly, two isomers (**29** and **31)** were also successfully discriminated in the study (Fig. 6).

Both non-isomeric compound identification and isomers discrimination have offered detailed MS elucidation in Additional file 1: S1, Additional file 2: S2, Additional file 3: S3, Additional file 4: S4, Additional file 5: S5, Additional file 6: S6, Additional file 7: S7, Additional file 8: S8, Additional file 9: S9, Additional file 10: S10, Additional file 11: S11, Additional file 12: S12, Additional file 13: 13, Additional file 14: S14, Additional file 15: S15, Additional file 16: S16, Additional file 17: S17, Additional file 18: S18, Additional file 19: S19, Additional file 20: S20, Additional file 21: S21, Additional file 22: S22, Additional file 23: S23, Additional file 24: S24, Additional file 25: S25, Additional file 26: S26, Additional file 27: S27, Additional file 28: S28, Additional file 29: S29, Additional file 30: S30, Additional file 31: S31, Additional file 32: S32, Additional file 33: S33, Additional file 344: S34, Additional file 35: S35, Additional file 36: S36, Additional file 37: S37, Additional file 38: S38, Additional file 39: S39, Additional file 40: S40, Additional file 41: S41, Additional file 42: S42, Additional file 43: S43, Additional file 44: S44, Additional file 45: S45, Additional file 46: S46, Additional file 47: S47, Additional file 48: S48, Additional file 49: S49, Additional file 50: S50, Additional file 51: S51. This has become a great contrast with the previous tentative identification works [71–73]. For example, Duan and colleagues used old document data (2015 and 2019) to “recognize” flavonoid vitexin in the newest experiment (2021). Obviously, there was no comparability between the old document data and new experiment data; Correspondingly, there was no detailed MS elucidation [73]. Some identification works might be arbitrarily, e.g., the identification of vitexin. This is because its highly similar isomer (isovitexin) has not been completely excluded. These tentative identification works may cause inappropriate (and even wrong) structure, to mislead the readers.

The present study however has detailed the structures of 52 compounds (**1–52**). Structurally, these putatively identified compounds covered 15 main structural types, that is, flavone-glycoside, flavone, lipid, saponin, phenolic acid, lignin, quinic acid derivative, isoflavone, chalcone, sugar, lkaloid, coumarin, naphthodianthrone, lactone, phenylpropanoid, and steroid. From the perspective of stereo-chemistry, most chiral atoms have been verified for the stereo-configuration. The putative identification, along with the isomers discrimination and stereo-configuration verification, have provided a foundation for Q-marker addition.

The Q-marker addition however is required to comply with five principles proposed by academician Chang-xiao Liu. These principles can be briefly described as traceability, testability, relevance to pharmacology, relevance to TCM theory, and specificity [74, 75]. Apparently, Liu’s principles have not completely excluded industrialization of Q-marker, and thus has not prohibited massive and illegal addition industrialized Q-marker into the Granule yet. This may lead to a tragedy similar to Sanlu Melamine Incident in China (2008), as predicted by our team [19].

To prevent a similar tragedy, a new principle named “non-industrialization” was recently proposed by our team [17]. According to the principle, one Q-marker candidate should not be easily obtained via industrialization. Therefore, 9 identified compounds were firstly excluded as Q-marker candidate, including D-gluconic acid (**1**), quinic acid (**2**), gallic acid (**3**), protocatechuic acid (**4**), methyl gallate (**6**), linoleic acid (**46**), palmitic acid (**47**), oleic acid (**48**), ethyl palmitate (**49**), and ethyl stearate (**50**). This is because these natural compounds could be industrially synthesized as well [76–82].

From other 43 compounds, four compounds were recommended as new Q-marker candidates, including caffeine (**7**), saikosaponin A (**28**), *S-*senkyunolide A (**40**), and magnolol (**44**), in accordance with the above principles (Table 5). In particular, four new Q-markers (**7, 28, 40,** and **44**), together with one revised one (**45**) possessed higher HOMO–LUMO energy gaps than the old Q-marker (**23**, Table 3). This has implied that the former five (**7, 28, 40, 44,** and **45**) would possess better traceability than the latter one (**23**). This is because that, high energy gap indicates high stability, from the angle of chemical thermodynamics. Thus, during the processes of manufacturing, handling, transportation, and metabolism, five Q-markers (**7, 28, 40, 44,** and **45**) would not be destroyed by external stimulation (e.g., illumination, heat, or catalyst), to show excellent traceability. Of these, caffeine (**7**) is a government-controlled chemical in China and cannot be massively and industrially synthesized. In short, all these candidates have complied with Liu’s and our principles (Table 4).

**Table 5:** The compliance of 5 new Q-marker candidates (**7** caffeine, **28** saikosaponin A, **40*** S-*senkyunolide A, **44** magnolol, and **45** 18β-glycyrrhetinic acid) with Liu’s and our principles

	7	28	40	44	45	Evidence
Traceability	Yes	Yes	Yes	Yes	Yes	Figure 9, Ref. [8]
Testability	Yes	Yes	Yes	Yes	Yes	Figure 1, Ref. [8]
Specificity	Yes	Yes	Yes	Yes	Yes	Ref. [3], Table 3 (Plant source)
Efficiency-relevance	Yes	Yes	Yes	Yes	Yes	Ref [3, 83, 84]
TCM-relevance	Yes	Yes	Yes	Yes	Yes	Table 1, Ref. [3]
Non-industrialization	Yes	Yes	Yes	Yes	Yes	Ref. [83, 85]

Liu’s principles might also be used to explain why “glycyrrhetinic acid” has been selected as the Pharmacopoeia Q-marker previously. The Q-marker was applied to characterize Gancao via a tedious and unreliable TLC operation [3]. However, in the study, the so-called “glycyrrhetinic acid” was clearly identified as 18β-glycyrrhetinic acid (**45**) rather than 18α-glycyrrhetinic acid. The complete separation of two glycyrrhetinic acids mainly relied on the difference in R.T. values, as seen in Additional file 44: S44. This difference however originated from molecular polarity. As seen in Table 4, two glycyrrhetinic acids displayed different dipole moments: 4.6173 Debye for 18α- and 3.1347 Debye for 18β-. In other words, different dipole moments have brought about different molecular polarities. Different molecular polarities have facilitated two glycyrrhetinic acids to be separated through a C_18_ adsorption column [61]. Obviously, the TLC analysis was incapable for separation of two glycyrrhetinic acids. In summary, it was necessary and feasible to revised the old Pharmacopoeia Q-marker “glycyrrhetinic acid” as 18β-glycyrrhetinic acid (**45**). As mentioned above, the revision can avoid the safety-incident similar to Thalidomide Disaster (1960s).

The revised Q-marker (**45**), together with four new candidates (**7, 28, 40,** and **44**) and the old Q-marker (hesperidin **23**) have re-constructed a new Q-markers system for Pharmacopoeia. In the system, 6 Q-markers showed different molecular polarities from each other; their dipole moments varied from -5.8110 to 5.8740 Debye (Table 4). This ensures that the six can be completely separated by a C_18_ adsorption column (Fig. 2).

From the perspective of analytic approach, the present UHPLC-Q-orbitrap MS/MS apparatus would be not an ideal choose, because it was too expensive and could not be afforded by most of pharmaceutical factories or drug control institute. For this reason, a lower revision LC–MS, i.e., UHPLC-ESI-Q-TOF–MS/MS, was used for *anti*-counterfeiting validation experiments, for its relative cheapness and popularity. The study thus used the UHPLC-ESI-Q-TOF–MS/MS to analyze 6 counterfeit *Wushicha* Granules, i.e., CWG 1 ~ CWG 6.

**Fig. 8:**
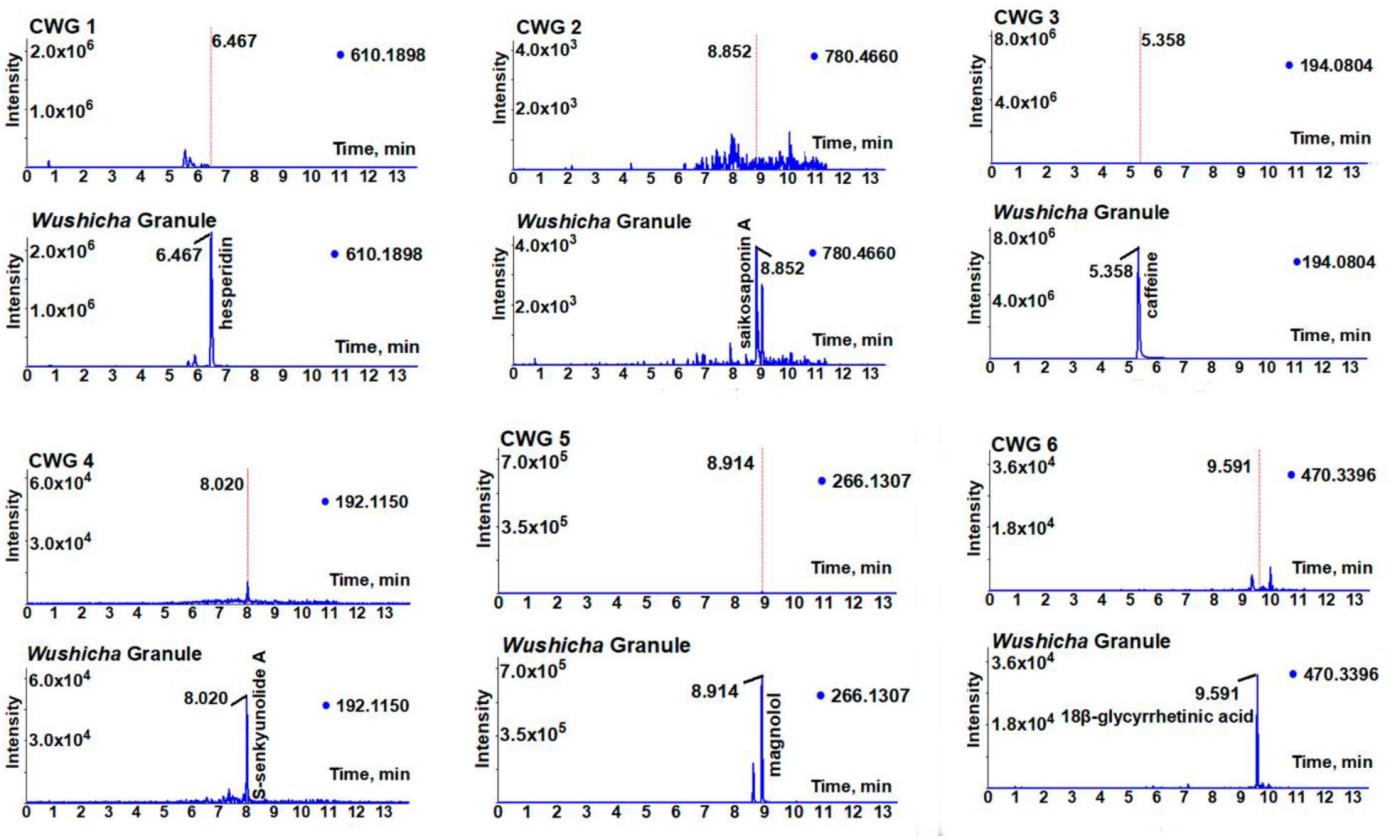
The results of *anti*-counterfeiting validation experiment of CWG 1–CWG 6. CWG 1, extraction of C_28_H_33_O_15_ (*m/z* 610, hesperidin); CWG 2, extraction of C_42_H_68_O_13_ (*m/z* 780, saikosaponin A); CWG 3, extraction of C_8_H_10_N_4_O_2_ (*m/z* 194, caffeine); CWG 4, extraction of C_12_H_16_O_2_ (*m/z* 193, *S*-senkyunolide A); CWG 5, extraction of C18H18O2 (*m/z* 266, magnolol); CWG 6, extraction of C_30_H_46_O_4_ (*m/z* 470.69, 18β-glycyrrhetinic acid). The analytic technology was UHPLC-ESI-Q-TOF–MS/MS

This first *anti*-counterfeiting validation experiment focused on CWG 1, one counterfeit *Wushicha* Granule without Chenpi and Zhishi. As seen in Fig. 8**,** CWG 1 showed no hesperidin (**23**) peak. In contrast, *Wushicha* Granule displayed a strong peak (2.0 × 10^6^) through the extraction of hesperidin formula (C_28_H_33_O_15_), which was equipped in LC–MS software. Owing hesperidin was the old Q-marker and could be only from either Chenpi or Zhishi (Table 3). The great contrast has clearly suggested the old Q-marker hesperidin was absent in CWG 1, implying that both Chenpi and Zhishi were absent in CWG 1. This has successfully recognized the counterfeit regarding both Chenpi and Zhishi. The successful instance further indicated that, our experiment based on LC–MS equipped extraction technology was feasible for *anti*-counterfeiting validation.

Using the LC–MS equipped extraction technology, CWG 2 was also analyzed in *anti*-counterfeiting validation experiment. As seen in Fig. 8**,** CWG 2 gave no saikosaponin A (**28**) peak; while *Wushicha* Granule gave a strong peak (7 × 10^5^). This difference suggested that, saikosaponin A was absent in CWG 2; and thus its corresponding CHM Chaihu was absent in CWG 2. Thus, the counterfeit regarding Chaihu has been successfully recognized by the UHPLC-ESI-Q-TOF–MS/MS analysis of Q-marker saikosaponin A (**28**).

Similarly, the counterfeits involved in Hongcha, Chuanxiong, and Houpo have also been successfully recognized by detection of their corresponding Q-markers, i.e., caffeine (**7**), *S*-senkyunolide A (**40**), magnolol (**44**), and 18β-glycyrrhetinic acid (**45**), respectively. Finally, the counterfeit concerning Gancao could be easily recognized by detection of 18β-glycyrrhetinic acid (**45**), a revised Q-marker. In summary, the validation experiments have successfully recognized 6 counterfeit *Wushicha* Granules (i.e., CWG 1 ~ CWG 6, Fig. 8), by means of analysis the corresponding Q-markers.

**Fig. 9:**
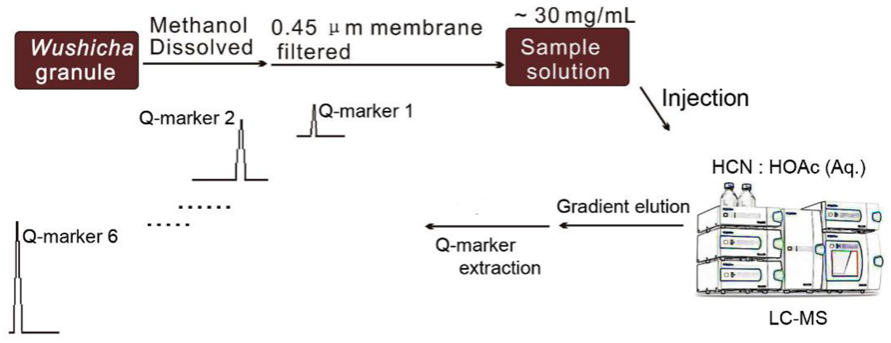
The flow chart of Q-markers analysis for *Wushicha* Granule

These Q-markers included one old Q-marker (**23**), one revised Q-marker (**45**), and 4 new Q-markers (**7, 28, 40,** and **44**). All these have constructed a new Q-markers system and corresponded 7 CHMs (Chenpi, Zhishi, Houpo, Chaihu, Gancao, Hongcha, and Chuanxiong). Accordingly, the Q-markers system can effectively recognize their counterfeits in *Wushicha* Granule. The Q-markers system, along with experimental description in *2.3 Section* and *2.8 Section*, have proposed an available procedure for analysis new Q-markers system (Fig. 9**)**. Through analysis of new Q-markers system, it can be judged whether there are counterfeits regarding Houpo, Chaihu, Gancao, Hongcha, Chuanxiong, Chenpi, and Zhishi. Apparently, all these will provide Pharmacopoeia with a reliable, feasible, and effective quality-control method concerning *Wushicha* Granule.

Finally, it should be noted that, (1) although the so-called “database-aided UHPLC-Q-orbitrap MS/MS putative identification strategy” has laid a solid foundation for Q-markers system update, however, the total amount of identified compounds is lower, compared with other “tentative identification strategy”. This will be improved through expanding the database in future.

**Fig. 10:**
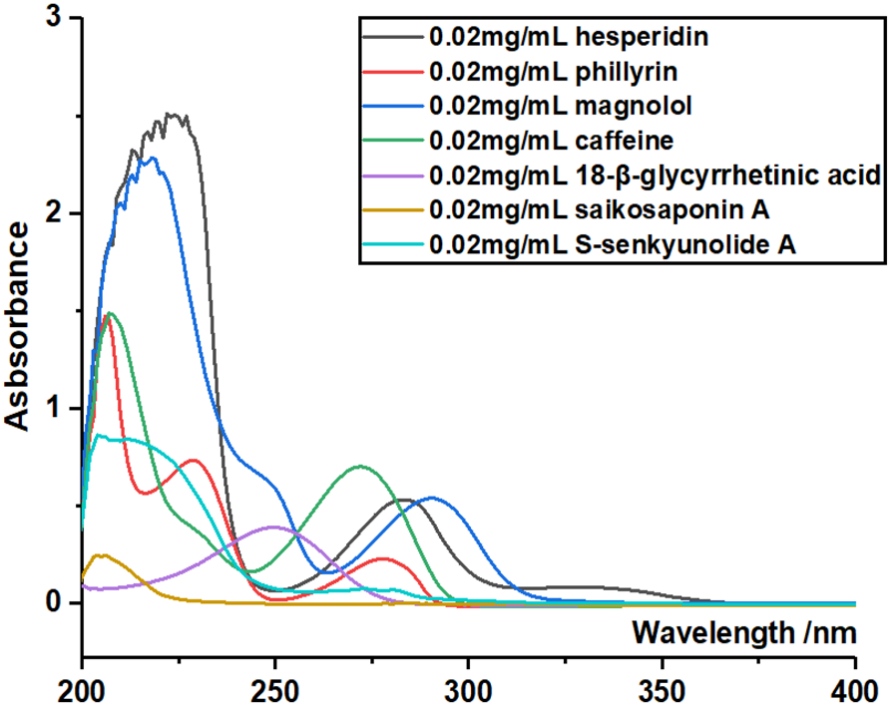
UV–vis spectra scanning of phillyrin, caffeine (**7**), hesperidin (**23**), saikosaponin A (**28**), *S-*senkyunolide A (**40**), magnolol (**44**), and 18β-glycyrrhetinic acid (**45**)

(2) As indicated by our UV–vis scanning experiment (Fig. 10), there was no co-wavelength for simultaneous analysis of all 6 Q-markers (**7, 23, 28, 40, 44,** and** 45**). All these have constructed). Therefore, the conventional HPLC–UV was not recommended as analytic approach for new Q-markers system.

(3) Platycodin D (**38**) is specific saponin for Jiegeng. As seen in Table 3, it has also been detected out in the study. However, its testability was not so good, for its low peak response (Fig. 2). Thus, it could be recommended as one optional Q-marker, when the analytic apparatus has high accuracy.

(4) As stated above, *Wushicha* Granule is a prescription consisting 19 CHMs. One CHM however is well known to enrich a good number of compounds; On other hand, one compound may distribute in different CHMs. This has made the Grandule to become an extremely complicated system, from the perspective chemistry. Thus, it is impossible to characterize all these CHMs. Nonetheless, our new Q-markers system has great improved the efficiency and reliability of quality-assessment method regarding *Wushicha* Granule in Pharmacopoeia. In the current Pharmacopoeia (2020), there was only one ole Q-marker (23) for reliable HPLC analysis. Aa a result, the total characterizing rate (TCR) was 10.5% (2 ÷ 19); while the specifically characterizing rate (SCR) was 0.0%, according to our recent definition [17]. For new Q-markers system, the TCR and SCR values were calculated as 36.8% (7 ÷ 19) and 26.3% (5 ÷ 19), respectively.

(5) The present study regarding Pharmacopoeia is not identical with the Pharmacopoeia itself. As stated by our tem [15, 19], the studies Pharmacopoeia have neither administrative compulsion nor legal authority. However, these studies can help Pharmacopoeia Commission to find a new and applicable Q-marker. This has highlighted the mission of our studies [15, 17, 19].

## Conclusions

By means of a novel database-aided UHPLC-Q-orbitrap MS/MS strategy, 48 non-isomeric compounds have been putatively identified; and two pairs of isomers have been successfully discriminated from *Wushicha* Granule; while A total of 52 compounds have been found from the Granule. Of these, 18β-glycyrrhetinic acid is recommended to replace the old Q-marker “glycyrrhetinic acid”, to prevent safety-incident. Meanwhile, four compounds (saikosaponin A, caffeine, *S-*senkyunolide A, and magnolol) are recommended as new Q-markers. Even through low version LC–MS technology, analysis of these Q-markers can effectively recognize six counterfeit *Wushicha* Granules. Thereby, they can prevent the counterfeiting in *Wushicha* Granule, and will improve the efficiency and reliability of Pharmacopoeia.

### Supplementary Information

**Additional file 1.** Identification of D-Gluconic acid (Cas 526-95-4, C_6_H_12_O_7_, M.W.196).

**Additional file 2. **Identification of Quinic acid (Cas 77-95-2, C_7_H_12_O_6_, M.W. 192.17).

**Additional file 3. **Identification of Gallic acid (Cas 149-91-7, C_7_H_6_O_5_, M.W. 170.12).

**Additional file 4. **Identification of protocatechuate (Cas 99-50-3, C_7_H_6_O_4_, M.W. 154.12).

**Additional file 5. **Identification of 5-Caffeoylquinic acid (Cas 906-33-2, C_16_H_18_O_9_, M.W. 354.311)

**Additional file 6. **Identification of methyl gallate (Cas 99-24-1, C_8_H_8_O_5_, M.W. 184.147).

**Additional file 7. **Identification of Caffeine (Cas 58-08-2,C_8_H_10_N_4_O_2_, M.W.194.191).

**Additional file 8. **Identification of puerarin (Cas 3681-99-0, C_21_H_20_O_9_, M.W. 416.38).

**Additional file 9.** Identification of Vicenin-2 (Cas 23666-13-9, C_27_H_30_O_15_, M.W. 594.52).

**Additional file 10. **Identification of schaftoside (Cas 51938-32-0, C_26_H_28_O_14_, M.W. 564.5)

**Additio.nal file 11. **Identification of Myricetin 3-O-galactoside (Cas 15648-86-9, C_21_H_20_O_13_, M.W. 480.37).

**Additional file 12.** Identification of Liquiritin (Cas 551-15-5, C_21_H_22_O_9_, M.W. 418.4).

**Additional file 13.** Identification of Vitexin (Cas 3681-93-4, C_21_H_20_O_10_, M.W. 432).

**Additional file 14. **Identification of Acteoside (Cas 61276-17-3, C_29_H_36_O_15_, M.W. 624.59).

**Additional file 15. **Identification of Scoparone (Cas 120-08-1, C_11_H_10_O_4_ M.W.206.19).

**Additional file 16. **Identification of (-)-Pinoresinol (Cas 81446-29-9, C_20_H_22_O_6_, M.W.358.39).

**Additional file 17. **Identification of isovitexin (Cas 38953-85-4, C_21_H_20_O_10_, M.W. 432.3775).

**Additional file 18. **Identification of isoquercitrin (Cas 21637-25-2, C_21_H_20_O_12_, M.W. 464.38).

**Additional file 19. **Identification of rutin (Cas 153-18-4, C_27_H_30_O_16_, M.W. 610.52).

**Additional file 20.** Identification of Naringin (Cas 10236-47-2, C_27_H_32_O_14_, M.W.580.53).

**Additional file 21. **Identification of Rosmarinic acid (Cas 20283-92-5, C_18_H_16_O_8_, M.W. 360.31).

**Additional file 22.** Identification of Hesperidin (Cas 520-26-3, C_28_H_34_O_15_, M.W.610.565).

**Additional file 23.** Identification of Isochlorogenic acid A (Cas 2450-53-5, C_25_H_24_O_12_, M.W.516.45).

**Additional file 24.** Identification of Myricetin (Cas 529-44-2, C_15_H_10_O_8_, M.W. 318.24).

**Additional file 25. **Identification of Astragalin (Cas 480-10-4, C_21_H_20_O_11_, M.W. 448.38).

**Additional file 26. **Identification of Isorhamnetin-3-o-β-d-glucoside (Cas 5041-82-7, C_22_H_22_O_12_, M.W. 478.4).

**Additional file 27.** Identification of Saikosaponin A (Cas 20736-09-8, C_42_H_68_O_13_, M.W. 780.982).

**Additional file 28. **Identification of Daidzein (Cas 486-66-8, C_15_H_10_O_4_, M.W. 254.24).

**Additional file 29.** Identification of Quercetin (Cas 117-39-5, C_15_H_10_O_7_, M.W. 302.23).

**Additional file 30.** Identification of 7,4'-dihydroxyflavone (Cas 2196-14-7, C_15_H_10_O_4_, M.W. 254).

**Additional file 31.** Identification of S-Naringenin (Cas 480-41-1, C_15_H_12_O_5_, M.W.272.25).

**Additional file 32.** Identification of naringenin chalcone (Cas 73692-50-9, C_15_H_12_O_5_, M.W. 272.25).

**Additional file 33.** Identification of Luteolin (Cas 491-70-3, C_15_H_10_O_6_, M.W.286.24).

**Additional file 34. **Identification of Hesperetin (Cas 520-33-2, C_16_H_14_O_6_, M.W. 302.28).

**Additional file 35. **Identification of Randaiol (Cas 87562-14-9, C_15_H_14_O_3_, M.W. 242.27).

**Additional file 36. **Identification of Isoliquiritigenin (Cas 961-29-5, C_15_H_12_O_4_, M.W. 256.257).

**Additional file 37. **Identification of Platycodin D (Cas 58479-68-8, C_57_H_92_O_28_, M.W. 1225.3).

**Additional file 38. **Identification of Formononetin (Cas 485-72-3, C_16_H_12_O_4_, M.W. 268.26).

**Additional file 39. **Identification of Senkyunolide A (Cas 63038-10-8, C_12_H_16_O_2_, M.W. 192.25).

**Additional file 40. **Identification of 3,5,6,7,8,3,4,-7-Methoxy-2-phenyl-4H-chromen-4-one (Cas 1178-24-1, C_22_H_24_O_9_, M.W. 432.421).

**Additional file 41. **Identification of Licoricesaponin H2 (Cas 118441-85-3, C_42_H_62_O_16_, M.W. 822.93).

**Additional file 42. **Identification of 5-Hydroxyflavone (Cas 491-78-1, C_15_H_10_O_3_, M.W. 238.24).

**Additional file 43. **Identification of Magnolol (Cas 528-43-8, C_18_H_18_O_2_, M.W. 266.32).

**Additional file 44. **Identification of 18β-glycyrrhetinic acid and exclusion of 18α- glycyrrhetinic acid.

**Additional file 45. **Identification of linoleic acid (Cas 60-33-3, C_18_H_32_O_2_, M.W. 280.4).

**Additional file 46. **Identification of palmitic Acid (Cas 57-10-3, C_16_H_32_O_2_, M.W. 256.4).

**Additional file 47. **Identification of Oleic Acid (Cas 112-80-1, C_18_H_34_O_2_, M.W. 282.468).

**Additional file 48. **Identification of palmitic acid ethyl ester (Cas 628-97-7, C_18_H_36_O_2_, M.W. 284.484).

**Additional file 49. **Identification of Ethyl Stearate (Cas 111-61-5, C_20_H_40_O_2_, M.W. 312.53).

**Additional file 50. **Identification of Hypericin (Cas 548-04-9, C_30_H_16_O_8_, M.W. 504.45).

**Additional file 51.** Identification of (+)-4-Cholesten-3-one (Cas 601-57-0, C_27_H_44_O, M.W. 384.65).

## Data Availability

All the data used to support the findings of this study are available from the corresponding author upon reasonable request.
