# Supplementary figures and images for: Database-aided UHPLC-Q-orbitrap MS/MS strategy putatively identifies 52 compounds from Wushicha Granule to propose anti-counterfeiting quality-markers for pharmacopoeia
Source: Chin Med. 2023 Sep 9;18:116. doi: 10.1186/s13020-023-00829-2 (PMC10492348; doi:10.1186/s13020-023-00829-2)

Additional file 1: Identification of D-Gluconic acid (Cas 526-95-4, C6H12O7, M.W.196)

Standard


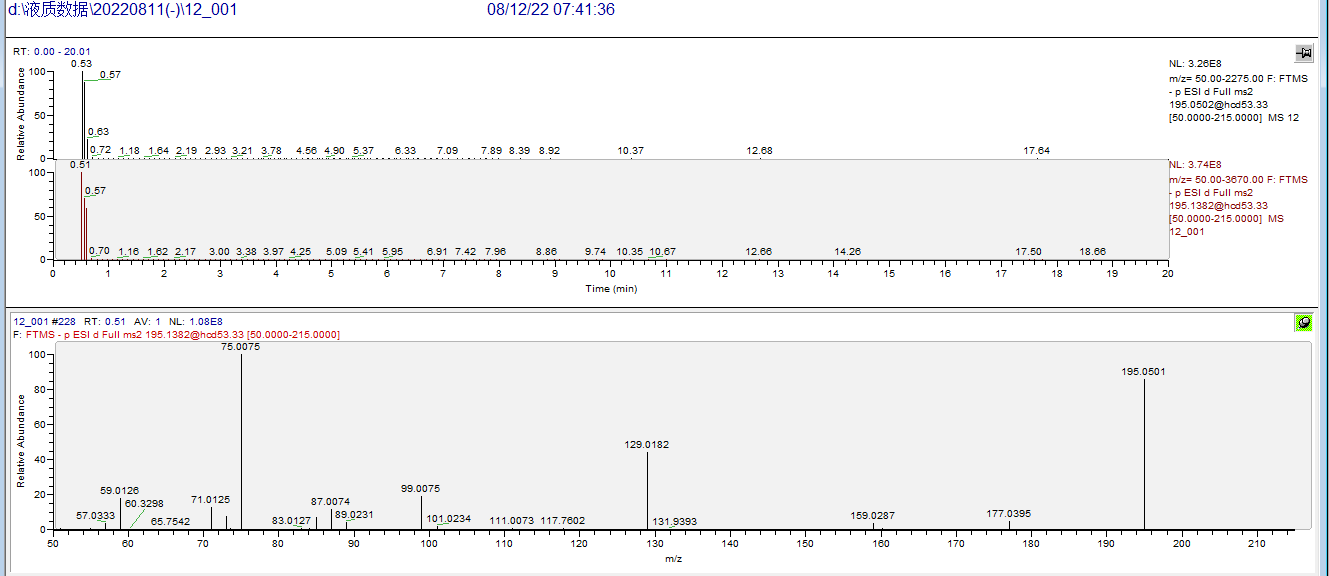


Sample


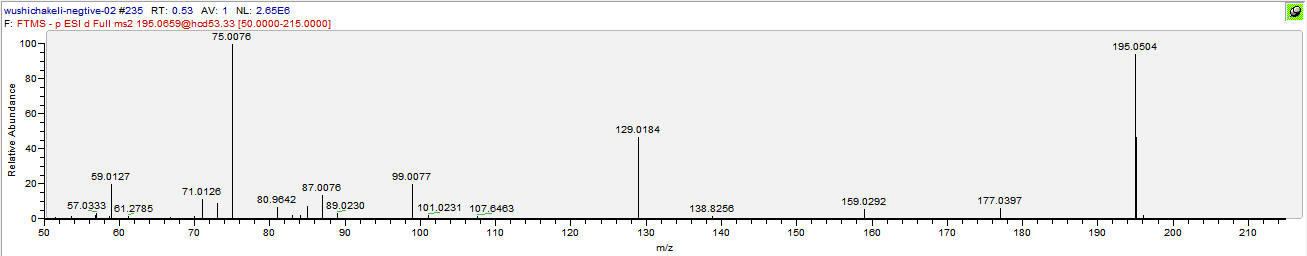

Supplement: Supplementary file 1 — Additional file 1. Identification of D-Gluconic acid (Cas 526-95-4, C6H12O7, M.W.196). [file 13020_2023_829_MOESM1_ESM.docx]

Additional file 2. Identification of Quinic acid (Cas 77-95-2, C7H12O6, M.W. 192.17)

Standard
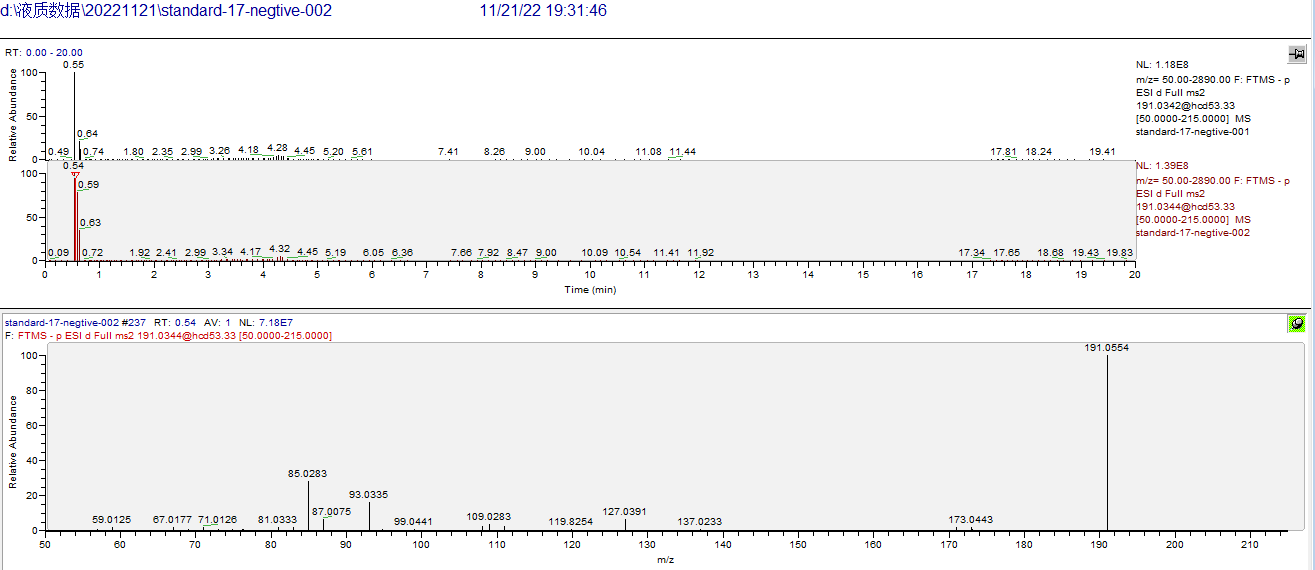


Sample


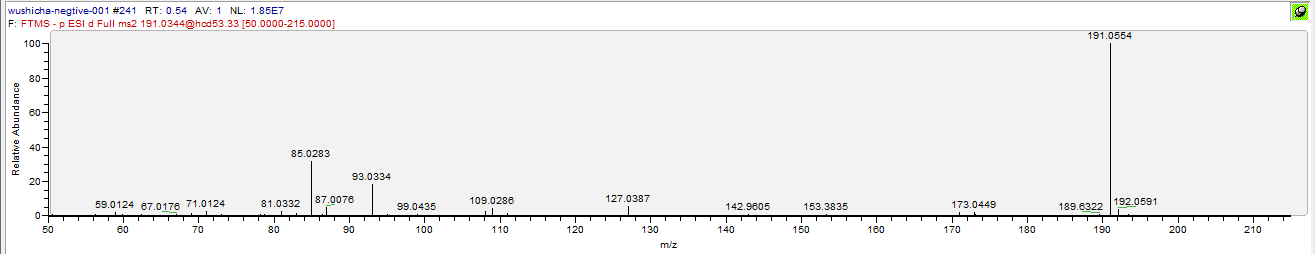

Supplement: Supplementary file 2 — Additional file 2. Identification of Quinic acid (Cas 77-95-2, C7H12O6, M.W. 192.17). [file 13020_2023_829_MOESM2_ESM.docx]

Additional file 3. Identification of Gallic acid (Cas 149-91-7, C7H6O5, M.W. 170.12)

Standard


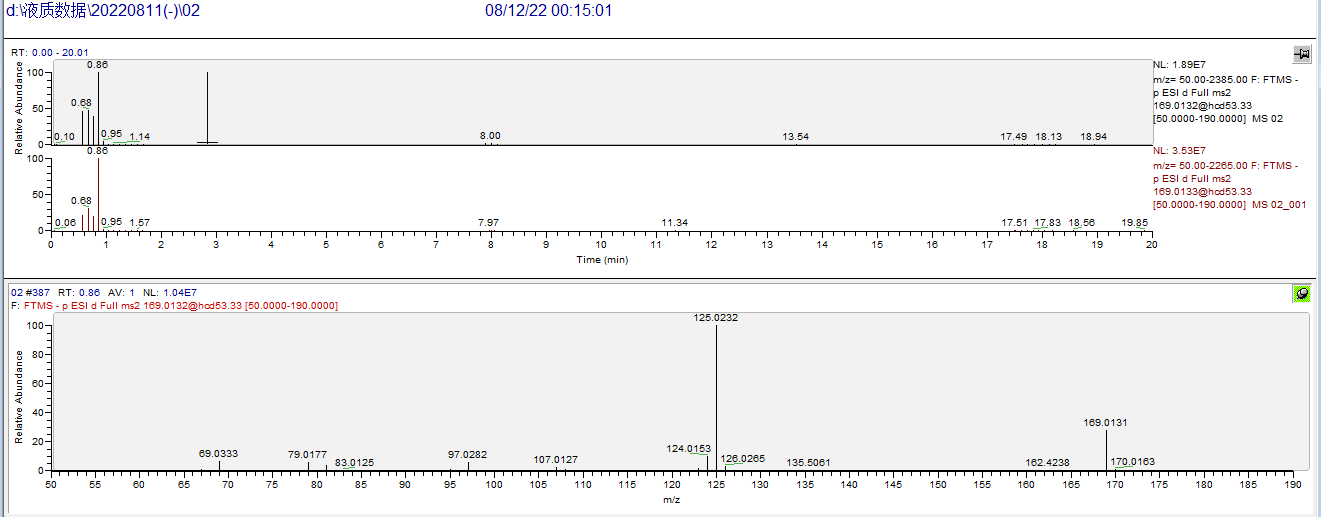


Sample


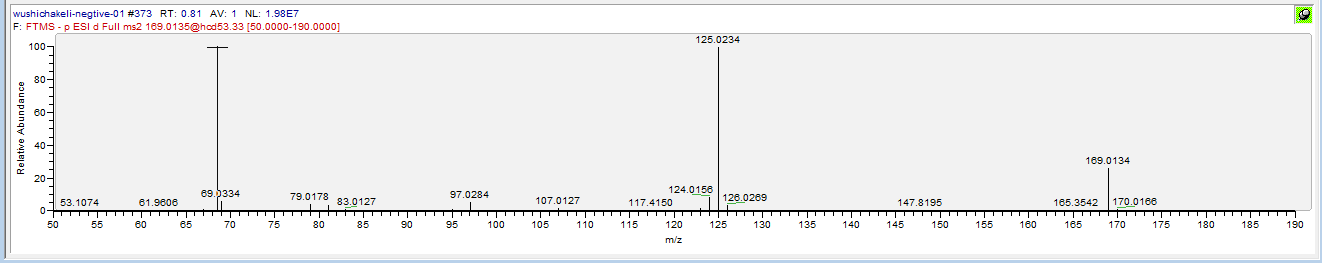

Supplement: Supplementary file 3 — Additional file 3. Identification of Gallic acid (Cas 149-91-7, C7H6O5, M.W. 170.12). [file 13020_2023_829_MOESM3_ESM.docx]

Additional file 4. Identification of protocatechuate (Cas 99-50-3, C7H6O4, M.W. 154.12)

Standard
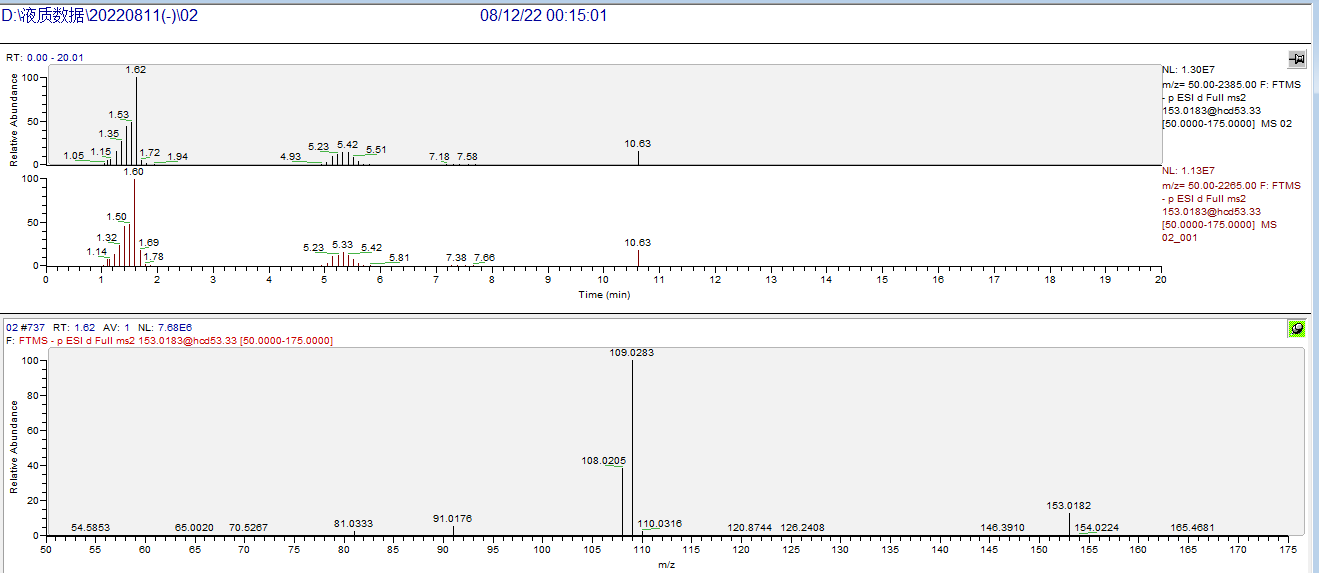


Sample


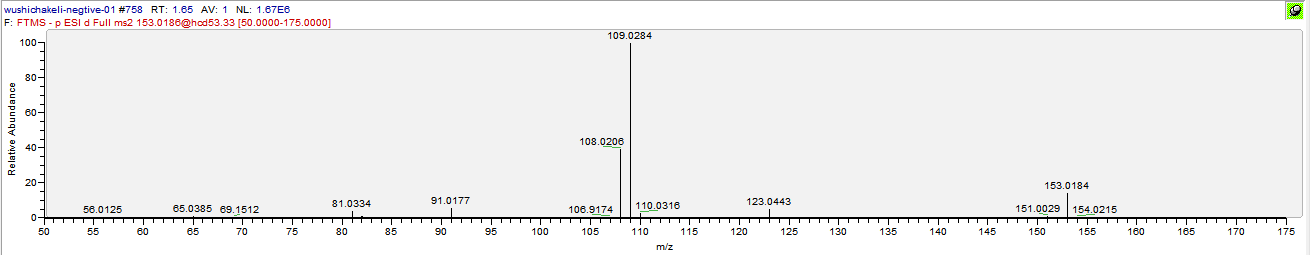

Supplement: Supplementary file 4 — Additional file 4. Identification of protocatechuate (Cas 99-50-3, C7H6O4, M.W. 154.12). [file 13020_2023_829_MOESM4_ESM.docx]

Additional file 5. Identification of 5-Caffeoylquinic acid (Cas 906-33-2, C16H18O9, M.W. 354.311)

Standard


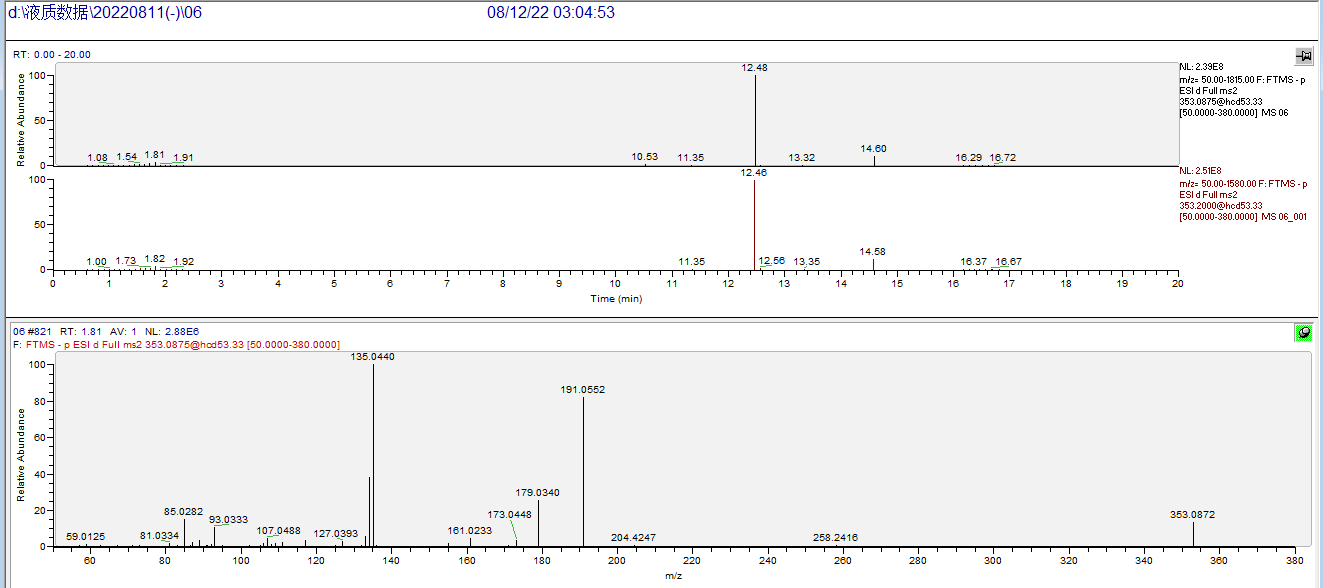


Sample


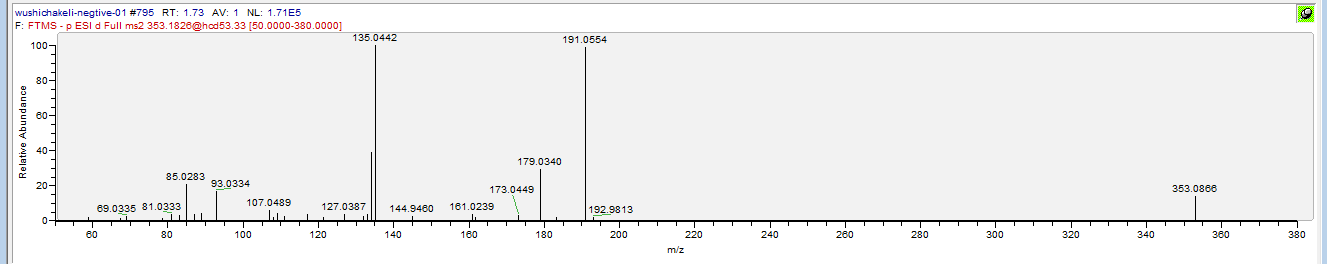

Supplement: Supplementary file 5 — Additional file 5. Identification of 5-Caffeoylquinic acid (Cas 906-33-2, C16H18O9, M.W. 354.311) [file 13020_2023_829_MOESM5_ESM.docx]

Additional file 6. Identification of methyl gallate (Cas 99-24-1, C8H8O5, M.W. 184.147)

Standard


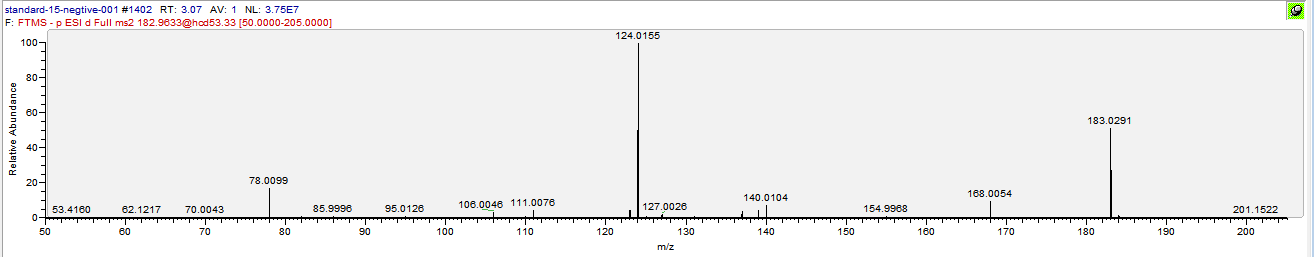


Sample


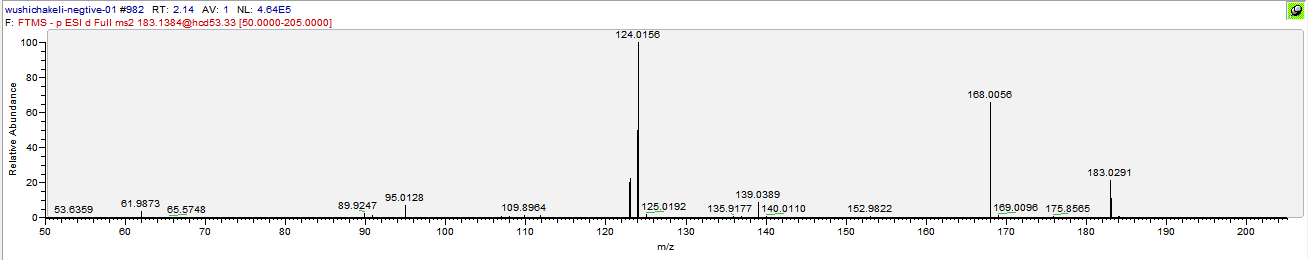

Supplement: Supplementary file 6 — Additional file 6. Identification of methyl gallate (Cas 99-24-1, C8H8O5, M.W. 184.147). [file 13020_2023_829_MOESM6_ESM.docx]

Additional file 7. Identification of Caffeine (Cas 58-08-2,C8H10N4O2, M.W.194.191)

Standard


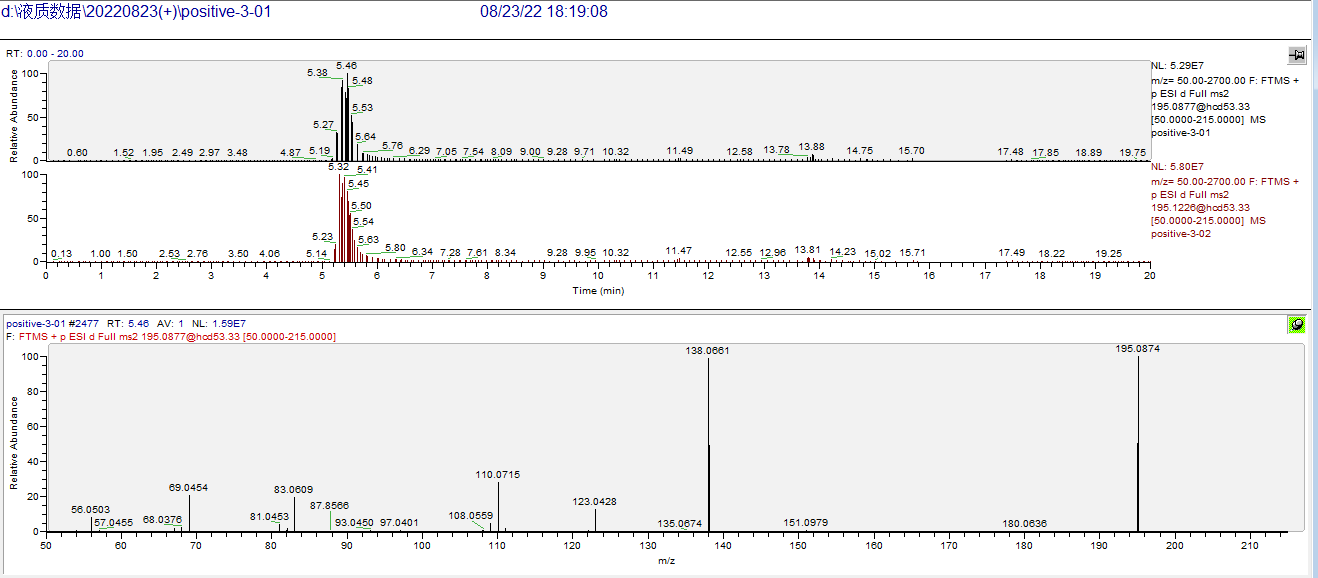


Sample


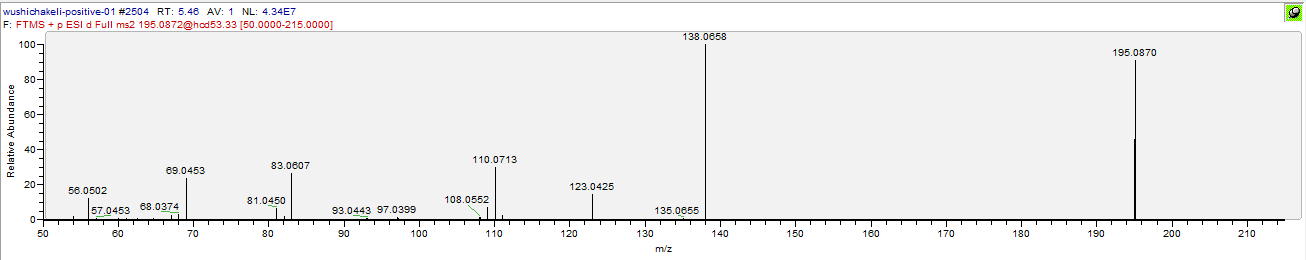

Supplement: Supplementary file 7 — Additional file 7. Identification of Caffeine (Cas 58-08-2,C8H10N4O2, M.W.194.191). [file 13020_2023_829_MOESM7_ESM.docx]

Additional file 8. Identification of puerarin (Cas 3681-99-0, C21H20O9, M.W. 416.38)

Standard


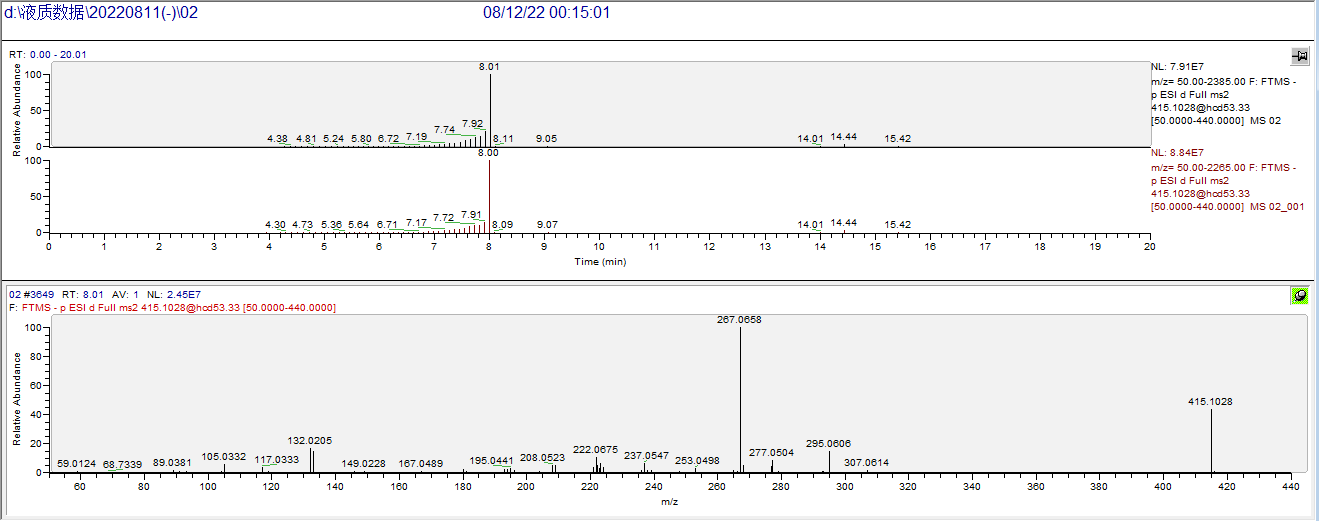


Sample


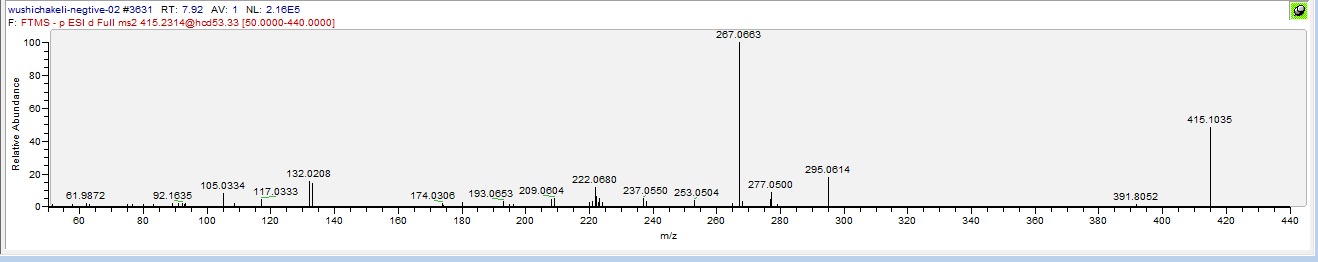

Supplement: Supplementary file 8 — Additional file 8. Identification of puerarin (Cas 3681-99-0, C21H20O9, M.W. 416.38). [file 13020_2023_829_MOESM8_ESM.docx]

Additional file 9. Identification of Vicenin-2 (Cas 23666-13-9, C27H30O15, M.W. 594.52)

Standard


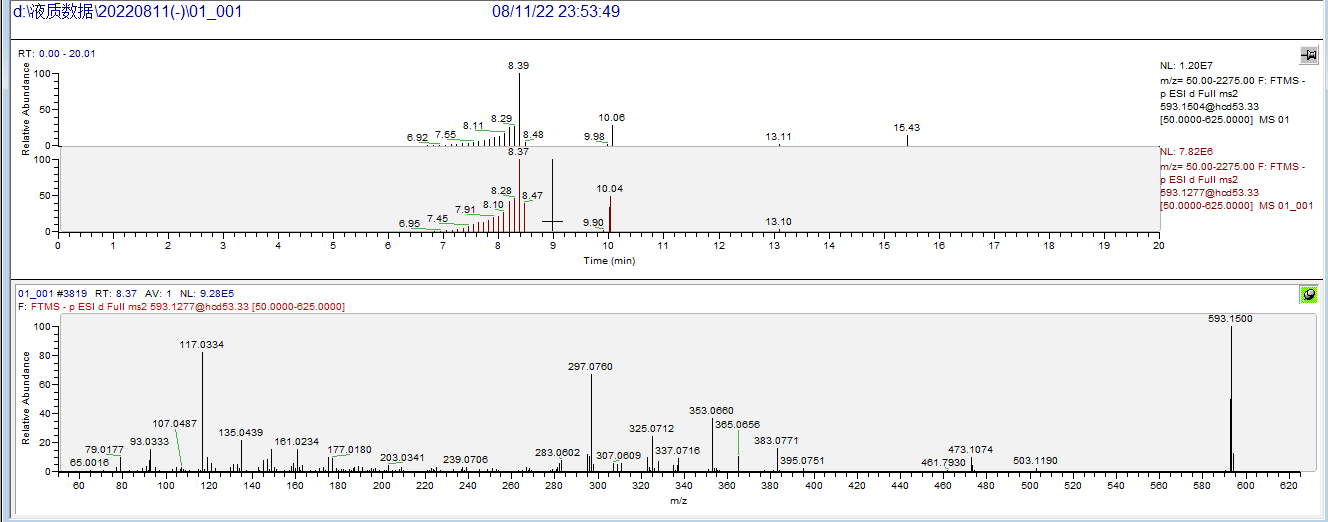


Sample


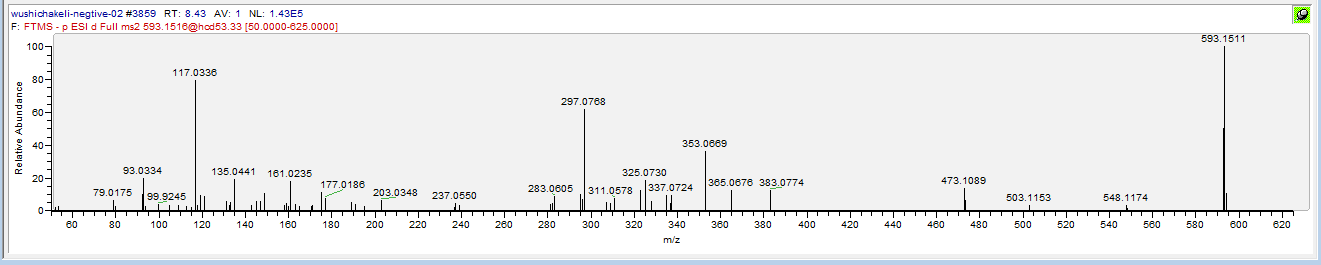

Supplement: Supplementary file 9 — Additional file 9. Identification of Vicenin-2 (Cas 23666-13-9, C27H30O15, M.W. 594.52). [file 13020_2023_829_MOESM9_ESM.docx]

Additional file 10. Identification of schaftoside (Cas 51938-32-0, C26H28O14, M.W. 564.5)

Standard


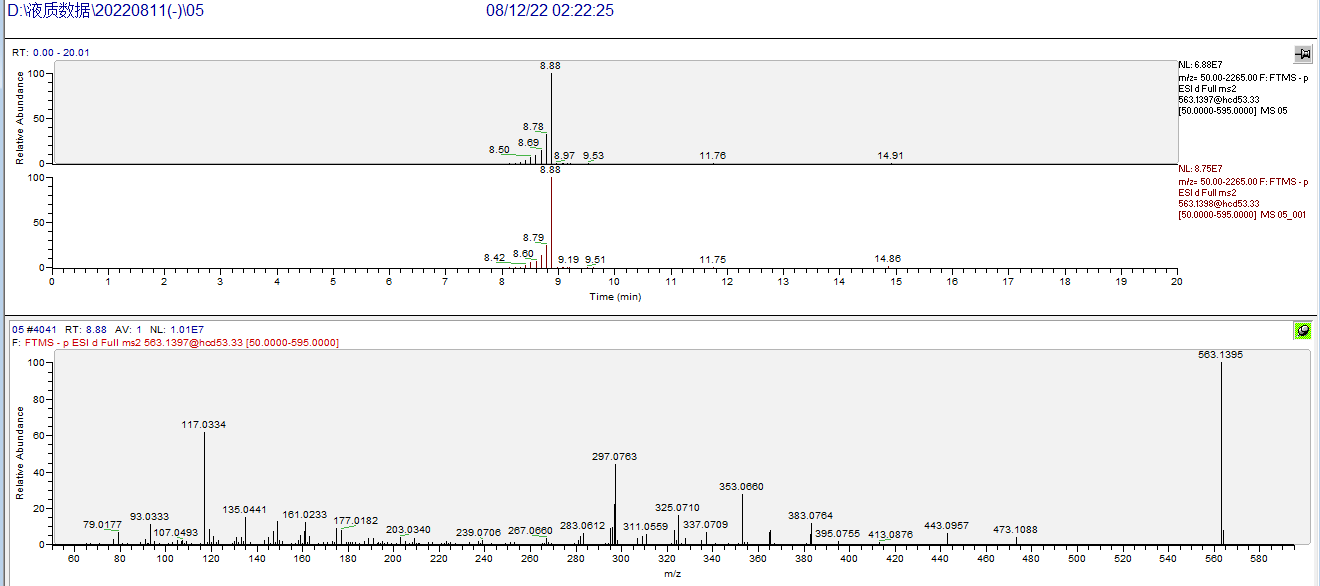


Sample


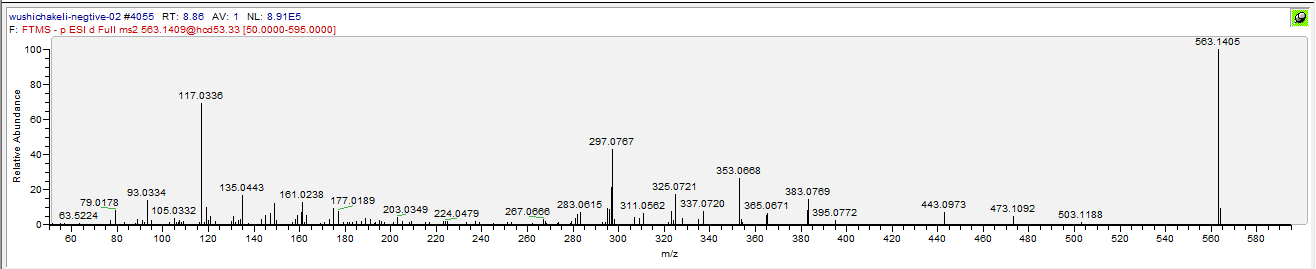

Supplement: Supplementary file 10 — Additional file 10. Identification of schaftoside (Cas 51938-32-0, C26H28O14, M.W. 564.5) [file 13020_2023_829_MOESM10_ESM.docx]

Additional file 11. Identification of Myricetin 3-O-galactoside (Cas 15648-86-9, C21H20O13, M.W. 480.37)

Standard


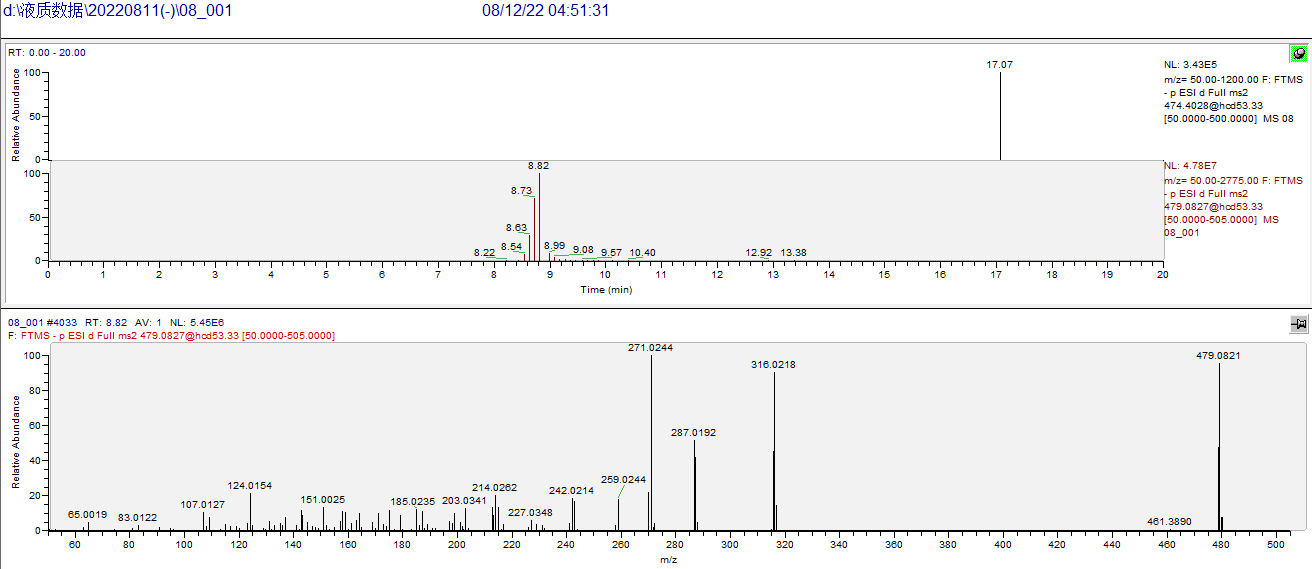


Sample


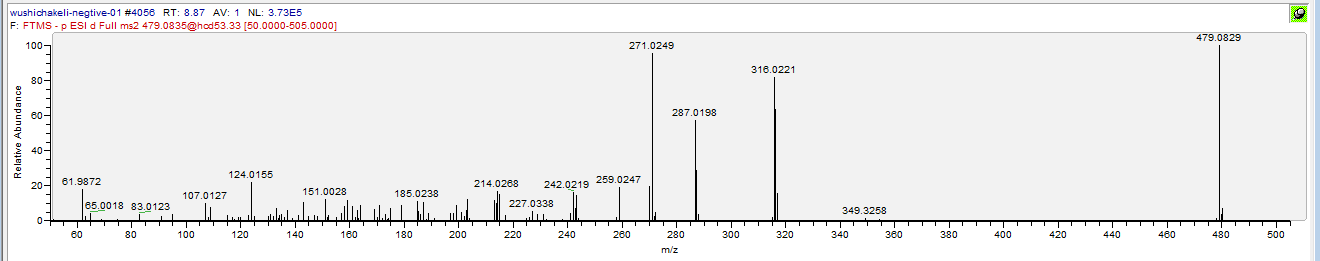

Supplement: Supplementary file 11 — Additio.nal file 11. Identification of Myricetin 3-O-galactoside (Cas 15648-86-9, C21H20O13, M.W. 480.37). [file 13020_2023_829_MOESM11_ESM.docx]

Additional file 12. Identification of Liquiritin (Cas 551-15-5, C21H22O9, M.W. 418.4)

Standard


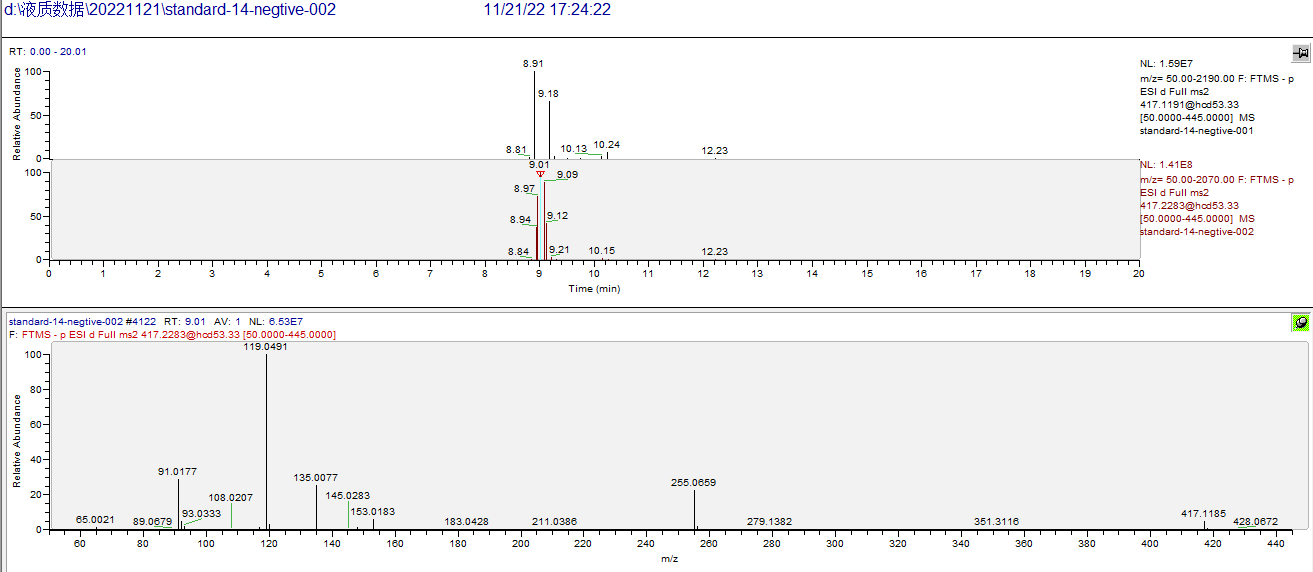


Sample


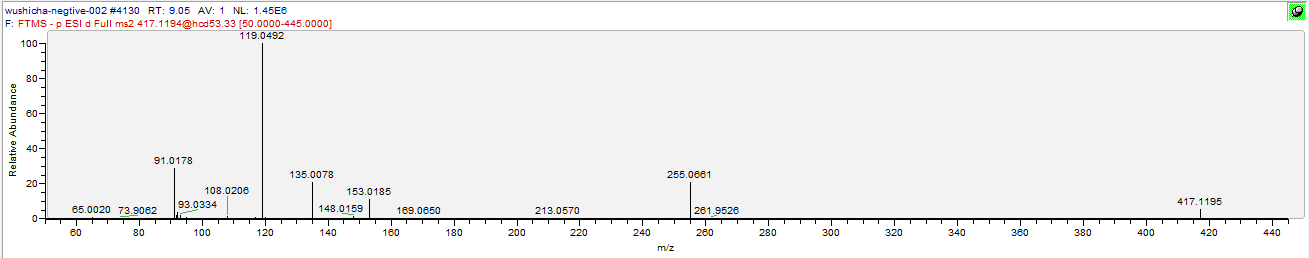

Supplement: Supplementary file 12 — Additional file 12. Identification of Liquiritin (Cas 551-15-5, C21H22O9, M.W. 418.4). [file 13020_2023_829_MOESM12_ESM.docx]

Additional file 13. Identification of Vitexin (Cas 3681-93-4, C21H20O10, M.W. 432)

Standard


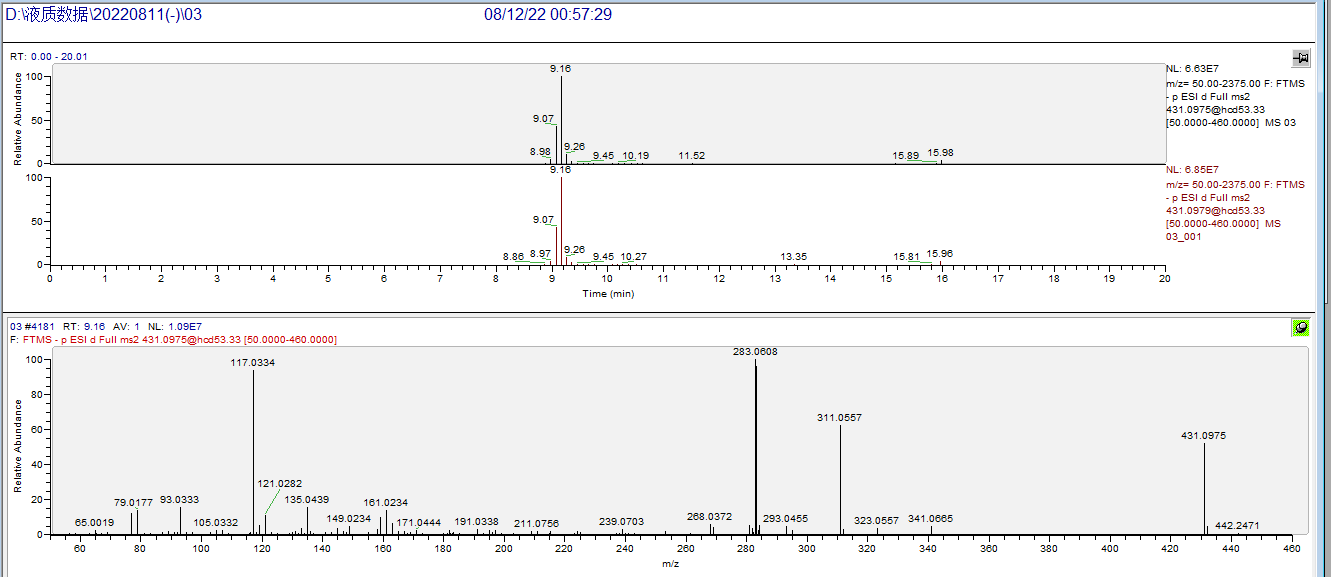


Sample


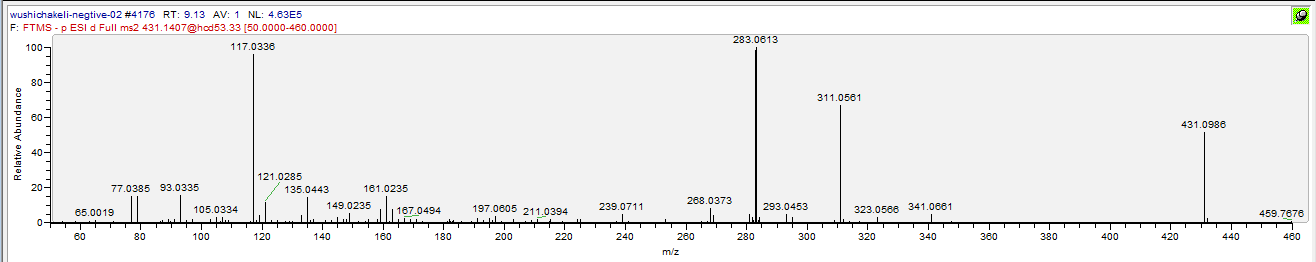

Supplement: Supplementary file 13 — Additional file 13. Identification of Vitexin (Cas 3681-93-4, C21H20O10, M.W. 432). [file 13020_2023_829_MOESM13_ESM.docx]

Additional file 14. Identification of Acteoside (Cas 61276-17-3, C29H36O15, M.W. 624.59)

Standard


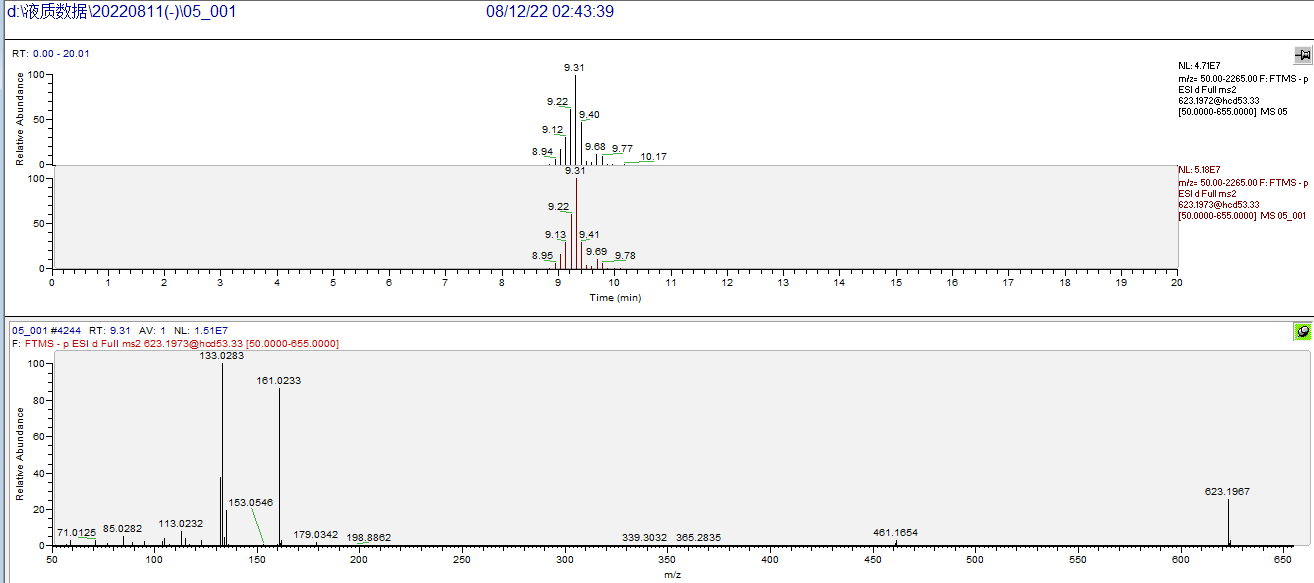


Sample


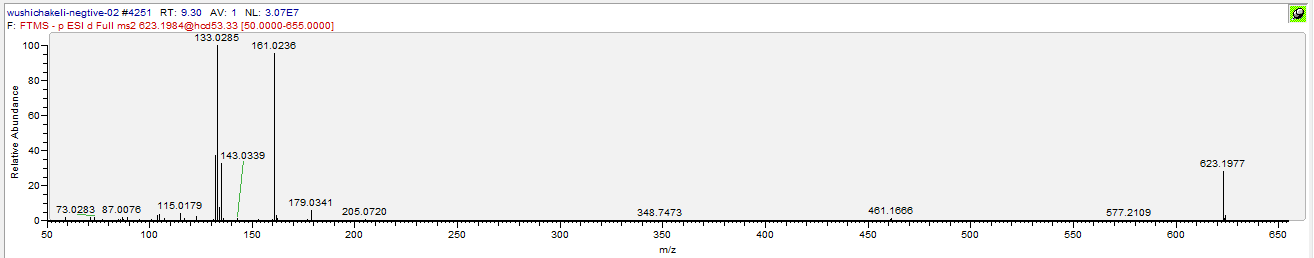

Supplement: Supplementary file 14 — Additional file 14. Identification of Acteoside (Cas 61276-17-3, C29H36O15, M.W. 624.59). [file 13020_2023_829_MOESM14_ESM.docx]

Additional file 15. Identification of Scoparone (Cas 120-08-1, C11H10O4 M.W.206.19)

Standard


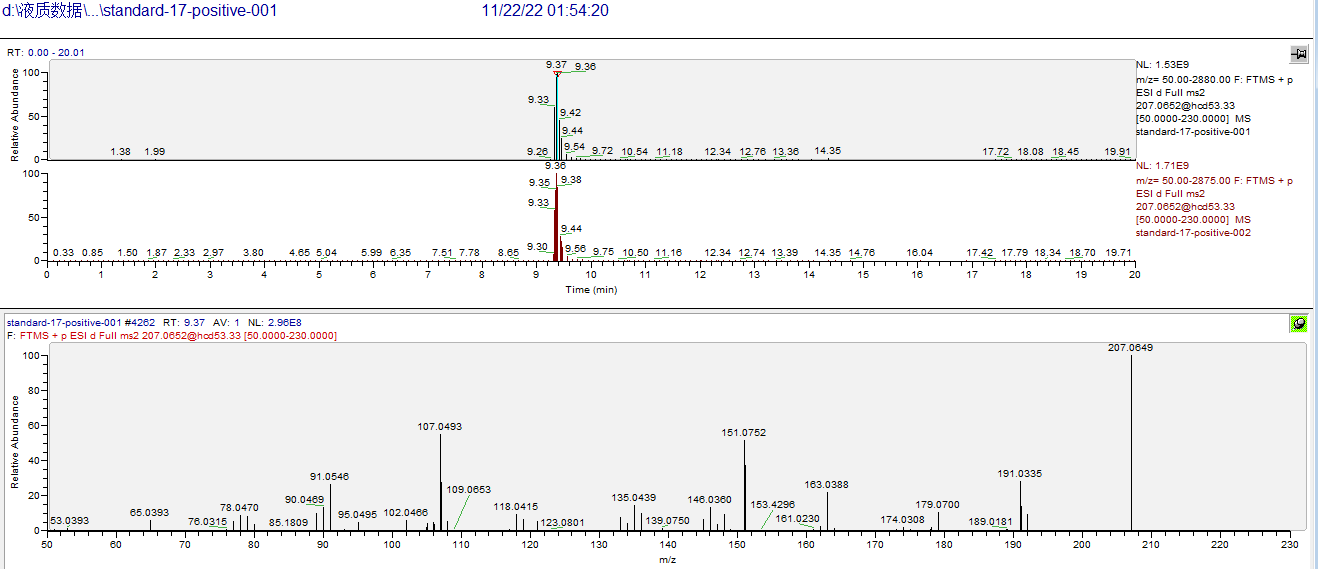


Sample


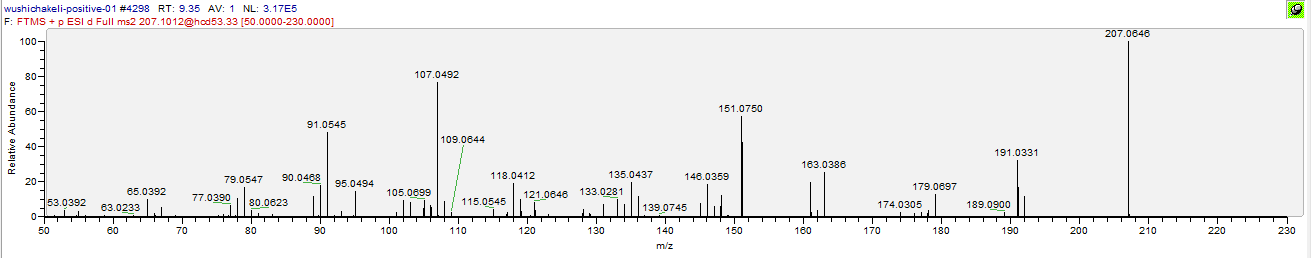

Supplement: Supplementary file 15 — Additional file 15. Identification of Scoparone (Cas 120-08-1, C11H10O4 M.W.206.19). [file 13020_2023_829_MOESM15_ESM.docx]

Additional file 16. Identification of (-)-Pinoresinol (Cas 81446-29-9, C20H22O6, M.W.358.39 )

Standard


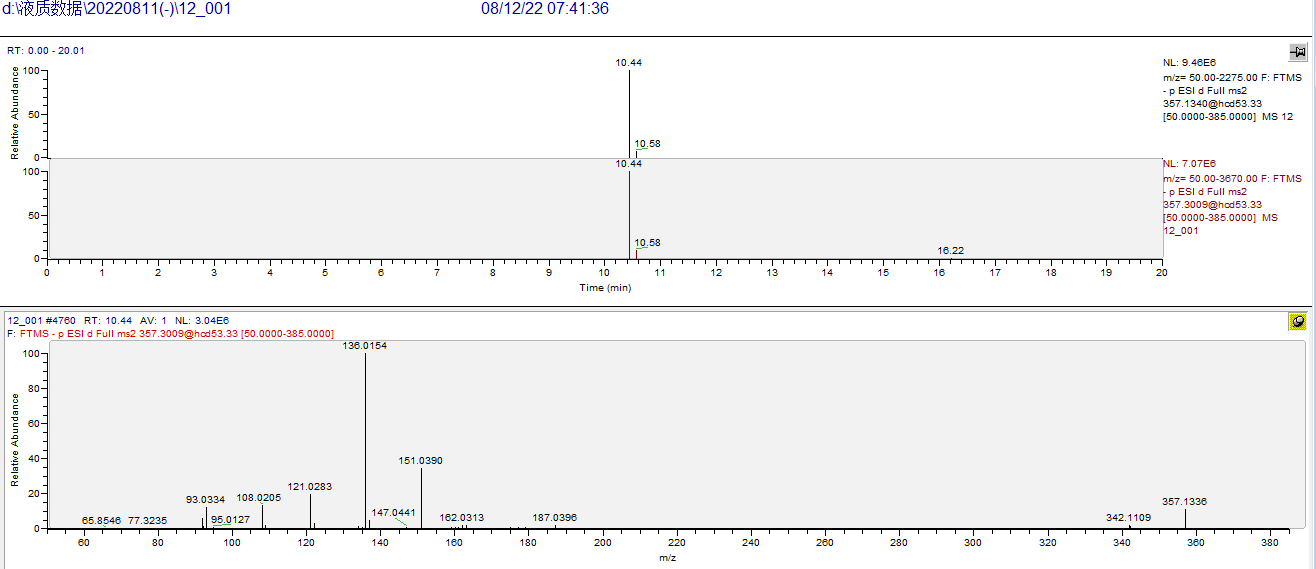


Sample


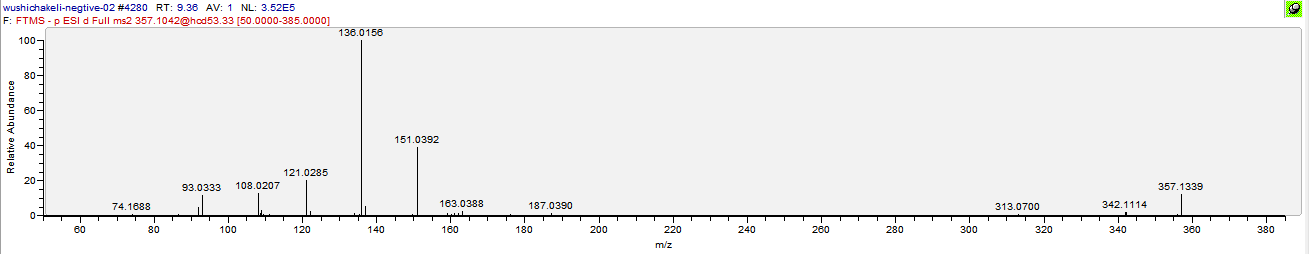

Supplement: Supplementary file 16 — Additional file 16. Identification of (-)-Pinoresinol (Cas 81446-29-9, C20H22O6, M.W.358.39). [file 13020_2023_829_MOESM16_ESM.docx]

Additional file 17. Identification of isovitexin (Cas 38953-85-4, C21H20O10, M.W. 432.3775)

Standard


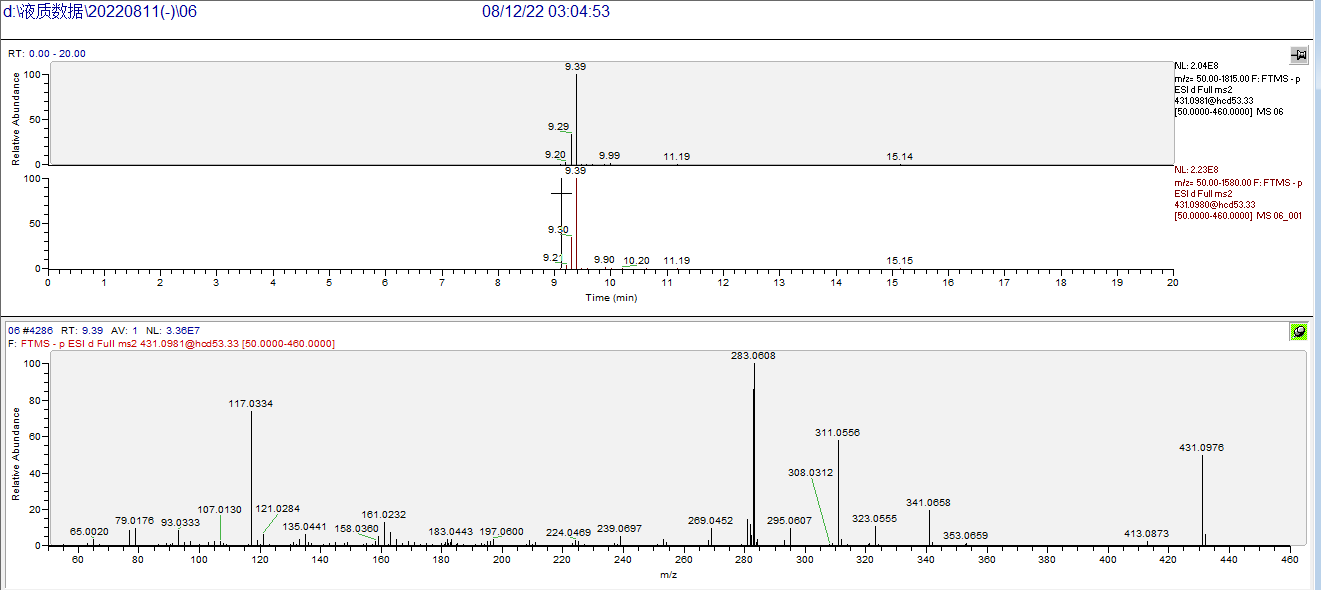


Sample


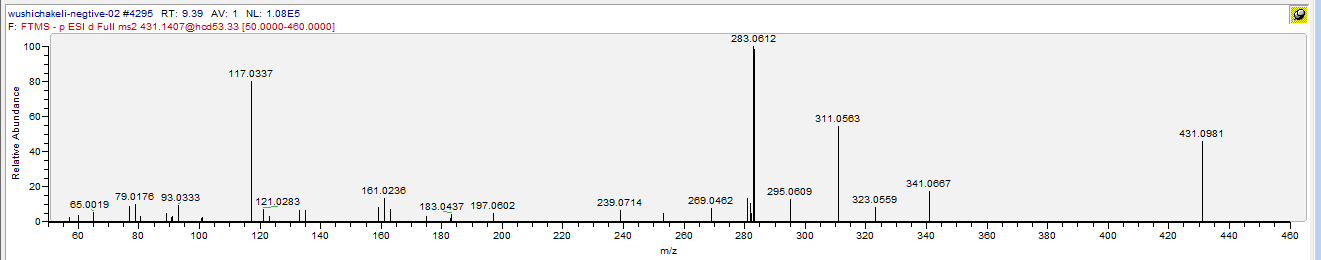

Supplement: Supplementary file 17 — Additional file 17. Identification of isovitexin (Cas 38953-85-4, C21H20O10, M.W. 432.3775). [file 13020_2023_829_MOESM17_ESM.docx]

Additional file 18. Identification of isoquercitrin (Cas 21637-25-2, C21H20O12, M.W. 464.38)

Standard


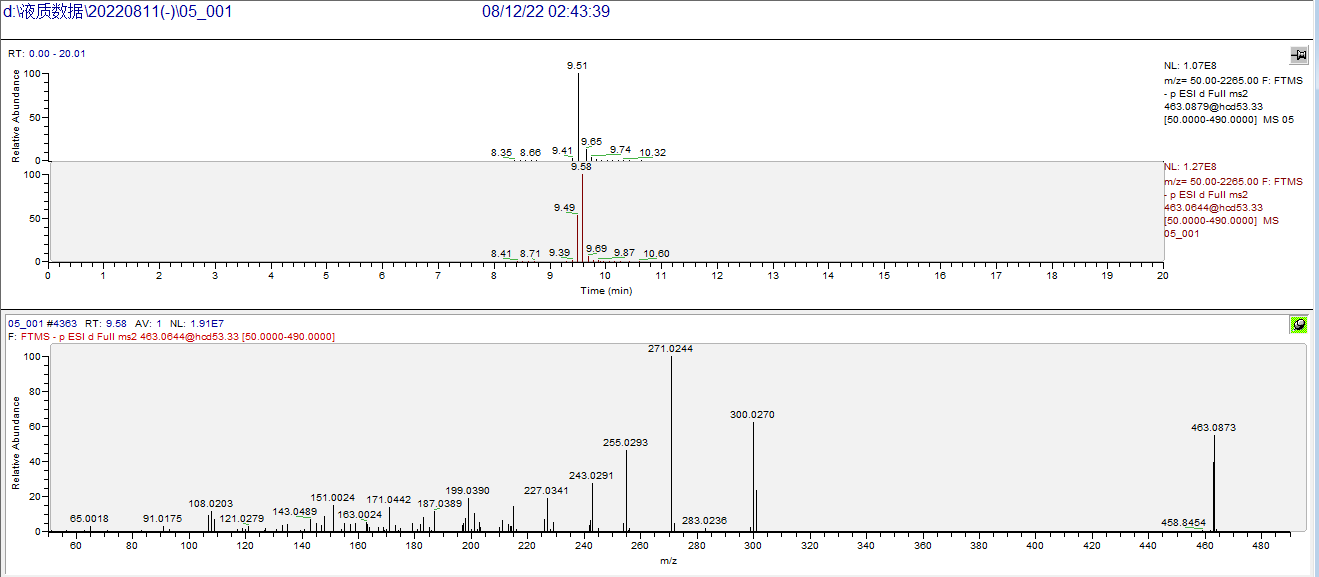


Sample


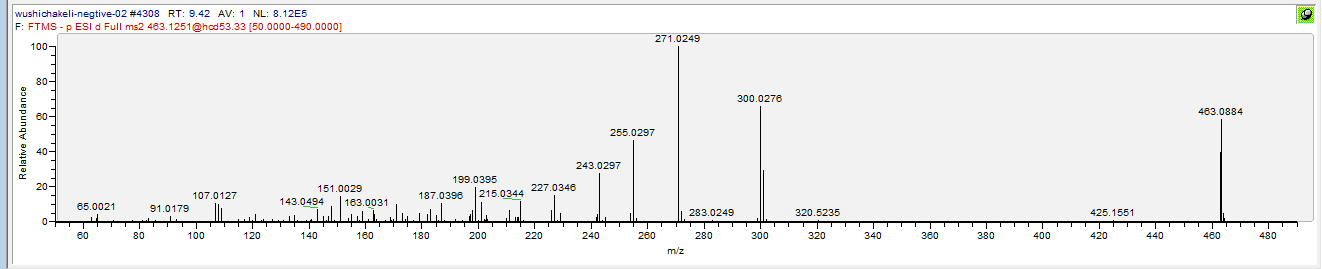

Supplement: Supplementary file 18 — Additional file 18. Identification of isoquercitrin (Cas 21637-25-2, C21H20O12, M.W. 464.38). [file 13020_2023_829_MOESM18_ESM.docx]

Additional file 19. Identification of rutin (Cas 153-18-4, C27H30O16, M.W. 610.52)

Standard


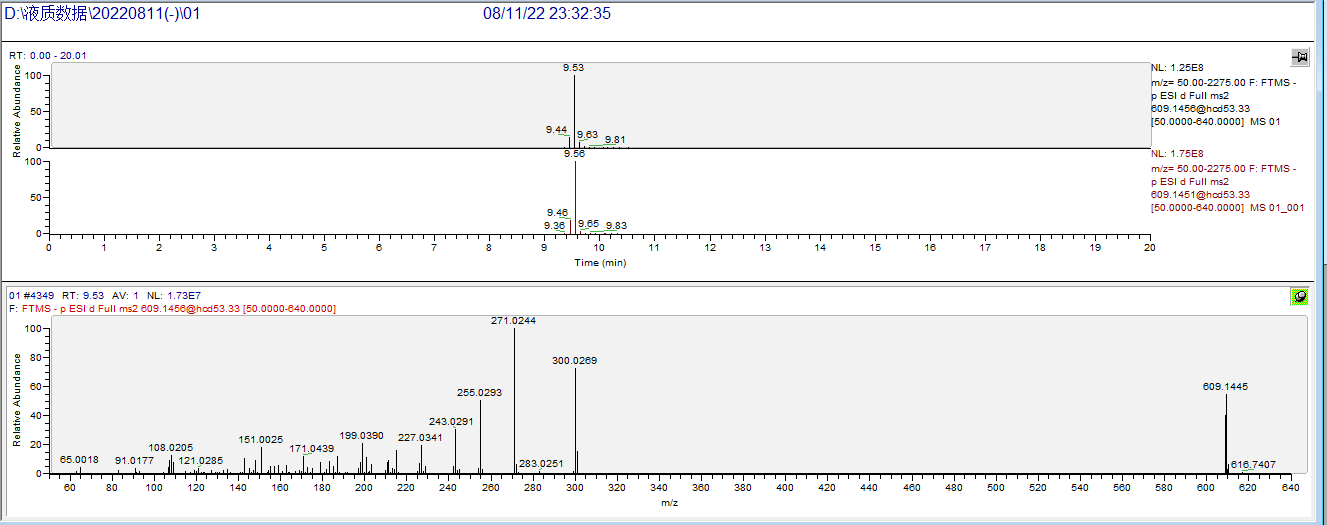


Sample


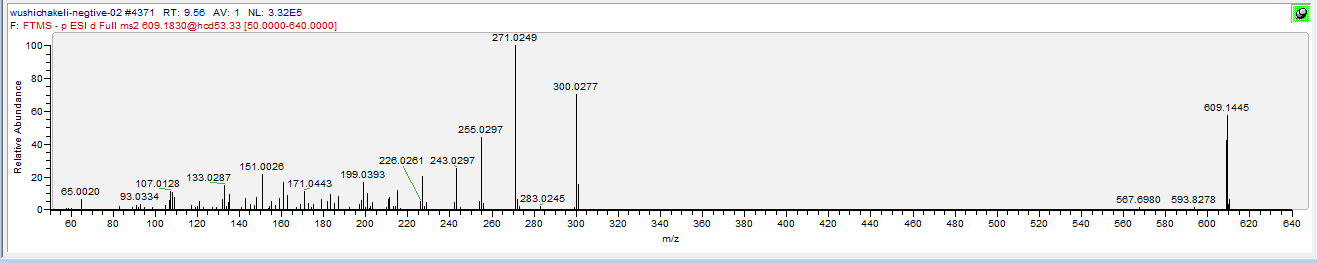

Supplement: Supplementary file 19 — Additional file 19. Identification of rutin (Cas 153-18-4, C27H30O16, M.W. 610.52). [file 13020_2023_829_MOESM19_ESM.docx]

Additional file 20. Identification of Naringin (Cas 10236-47-2, C27H32O14, M.W.580.53 )

Standard


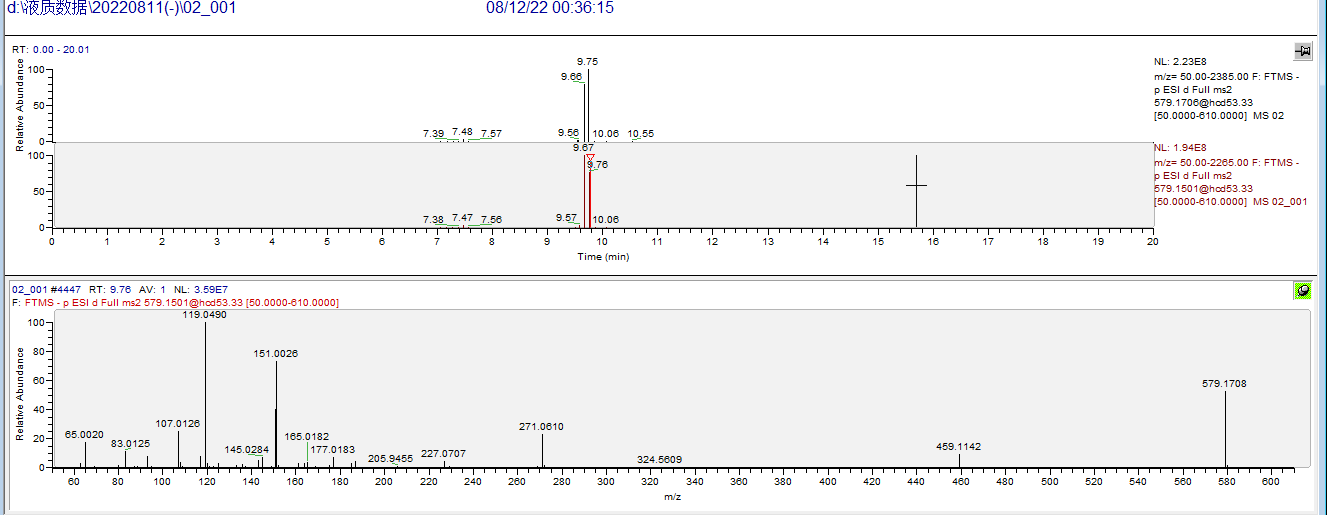


Sample


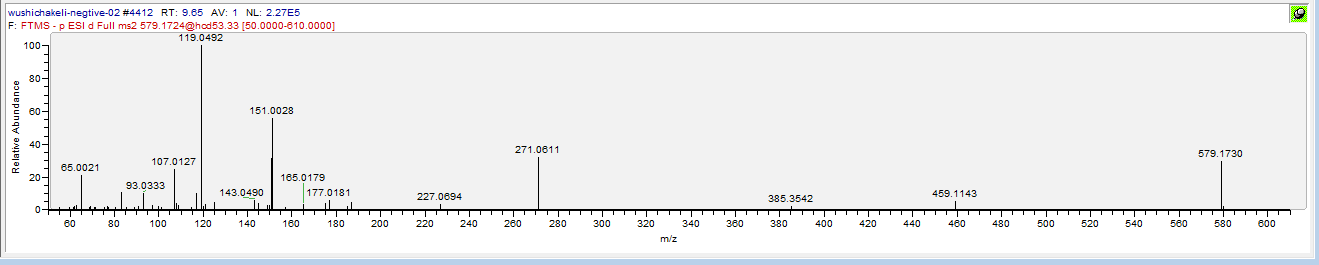

Supplement: Supplementary file 20 — Additional file 20. Identification of Naringin (Cas 10236-47-2, C27H32O14, M.W.580.53). [file 13020_2023_829_MOESM20_ESM.docx]

Additional file 21. Identification of Rosmarinic acid (Cas 20283-92-5, C18H16O8, M.W. 360.31)

Standard


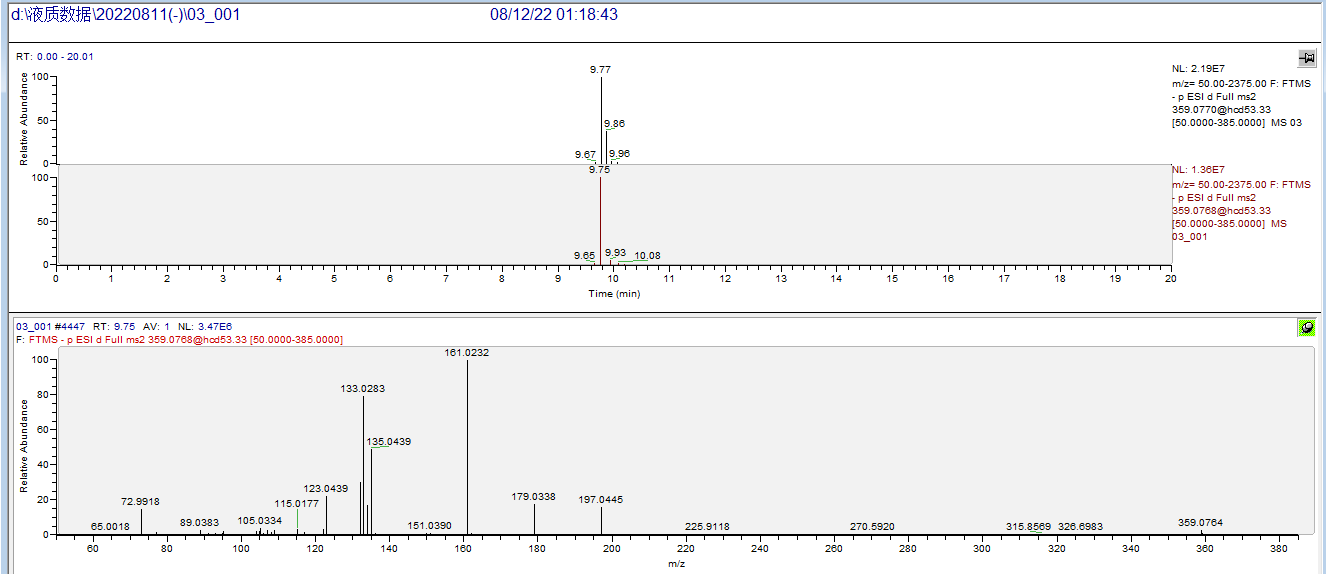


Sample


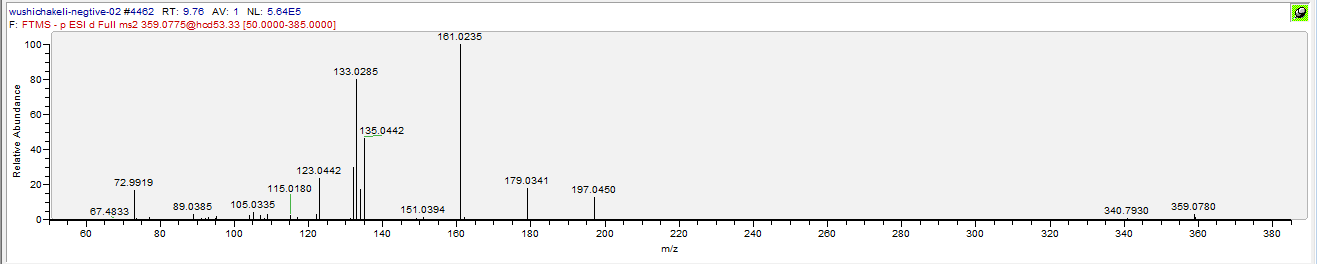

Supplement: Supplementary file 21 — Additional file 21. Identification of Rosmarinic acid (Cas 20283-92-5, C18H16O8, M.W. 360.31). [file 13020_2023_829_MOESM21_ESM.docx]

Additional file 22. Identification of Hesperidin (Cas 520-26-3, C28H34O15, M.W.610.565 )

Standard


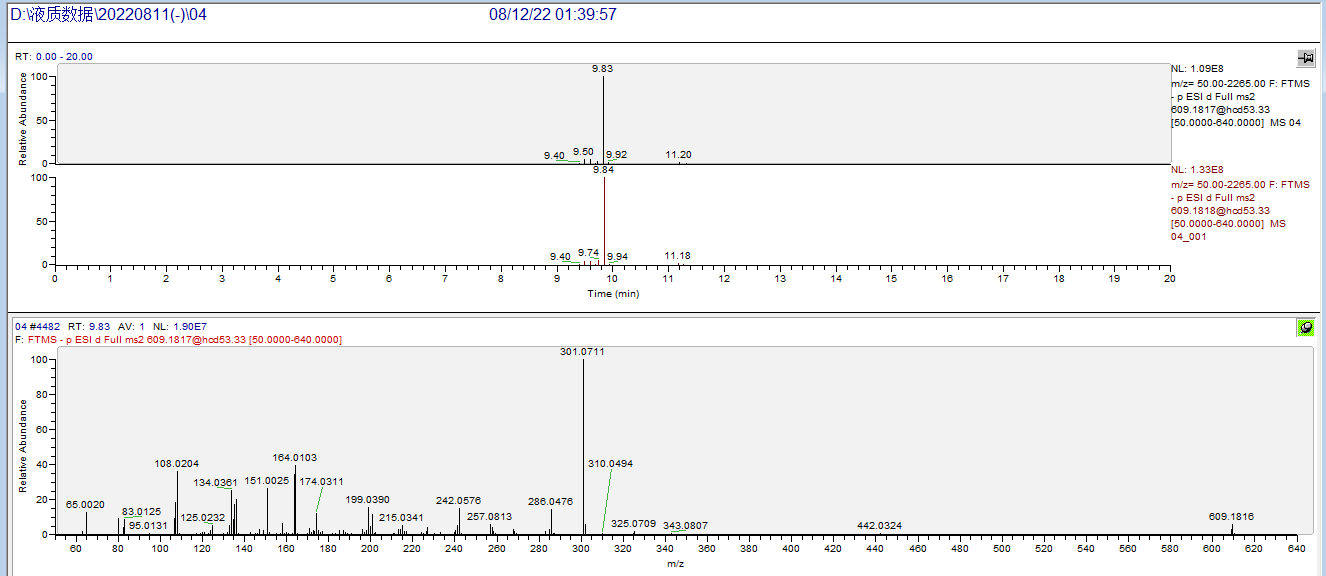


Sample


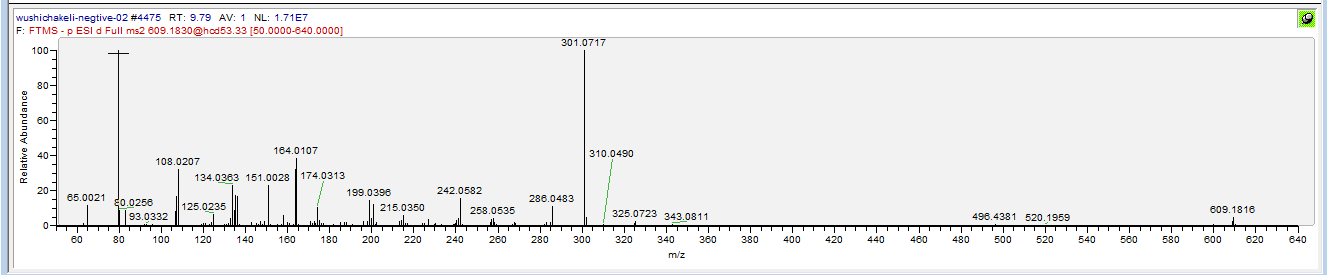

Supplement: Supplementary file 22 — Additional file 22. Identification of Hesperidin (Cas 520-26-3, C28H34O15, M.W.610.565). [file 13020_2023_829_MOESM22_ESM.docx]

Additional file 23. Identification of Isochlorogenic acid A (Cas 2450-53-5, C25H24O12, M.W.516.45)

Standard


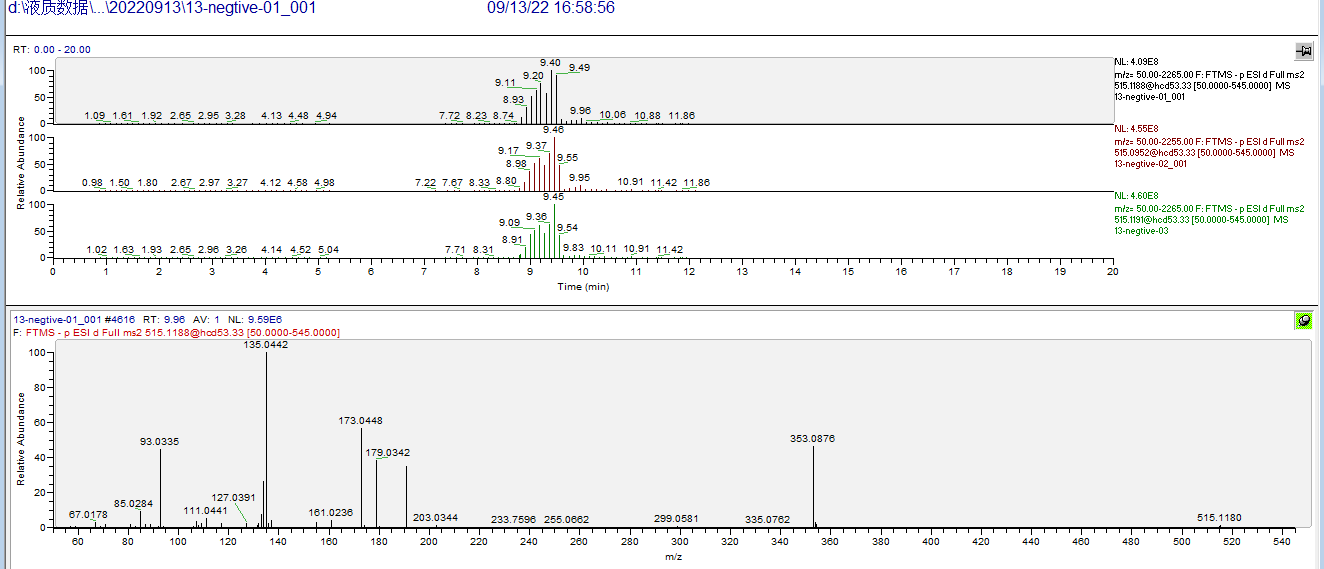


Sample


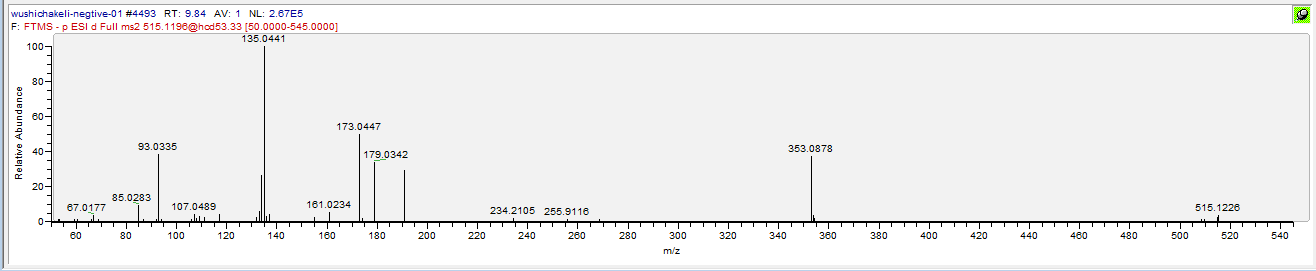

Supplement: Supplementary file 23 — Additional file 23. Identification of Isochlorogenic acid A (Cas 2450-53-5, C25H24O12, M.W.516.45). [file 13020_2023_829_MOESM23_ESM.docx]

Additional file 24. Identification of Myricetin (Cas 529-44-2, C15H10O8, M.W. 318.24)

Standard


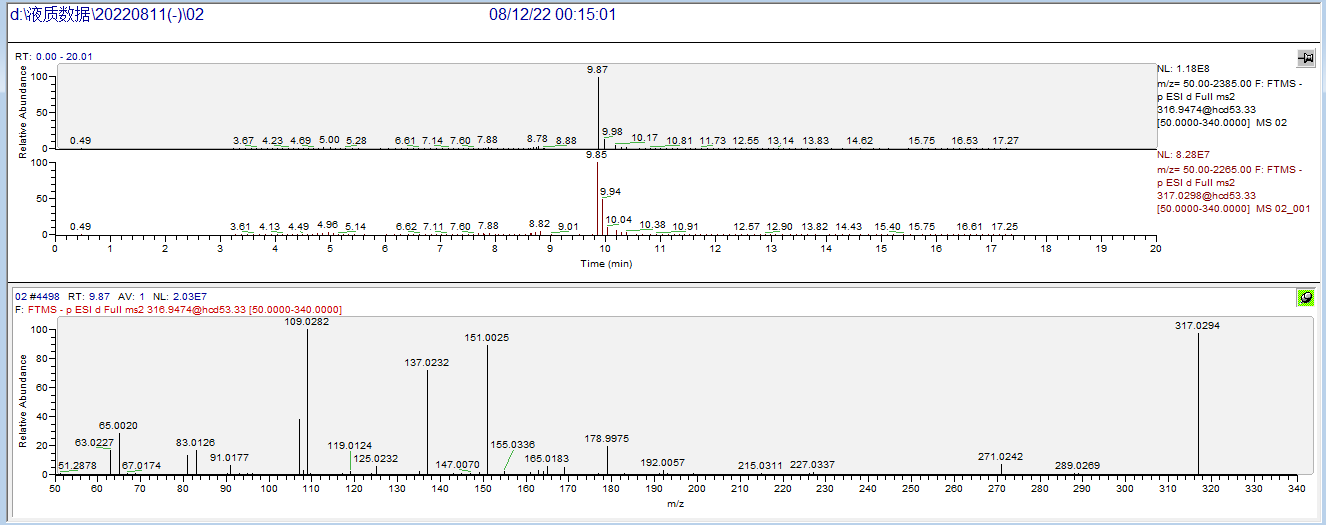


Sample


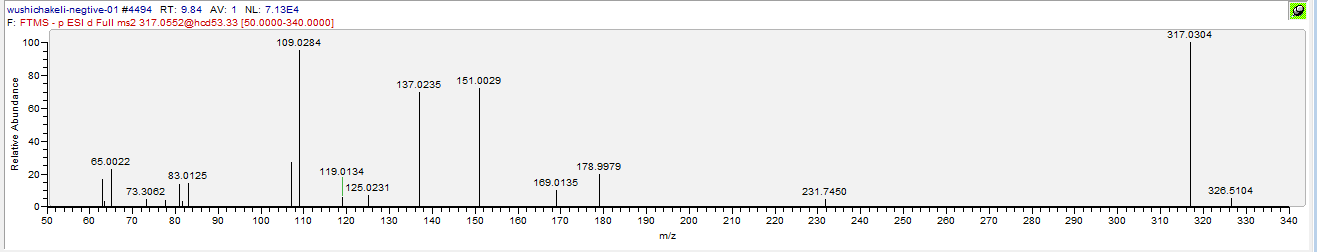

Supplement: Supplementary file 24 — Additional file 24. Identification of Myricetin (Cas 529-44-2, C15H10O8, M.W. 318.24). [file 13020_2023_829_MOESM24_ESM.docx]

Additional file 25. Identification of Astragalin (Cas 480-10-4, C21H20O11, M.W. 448.38)

Standard


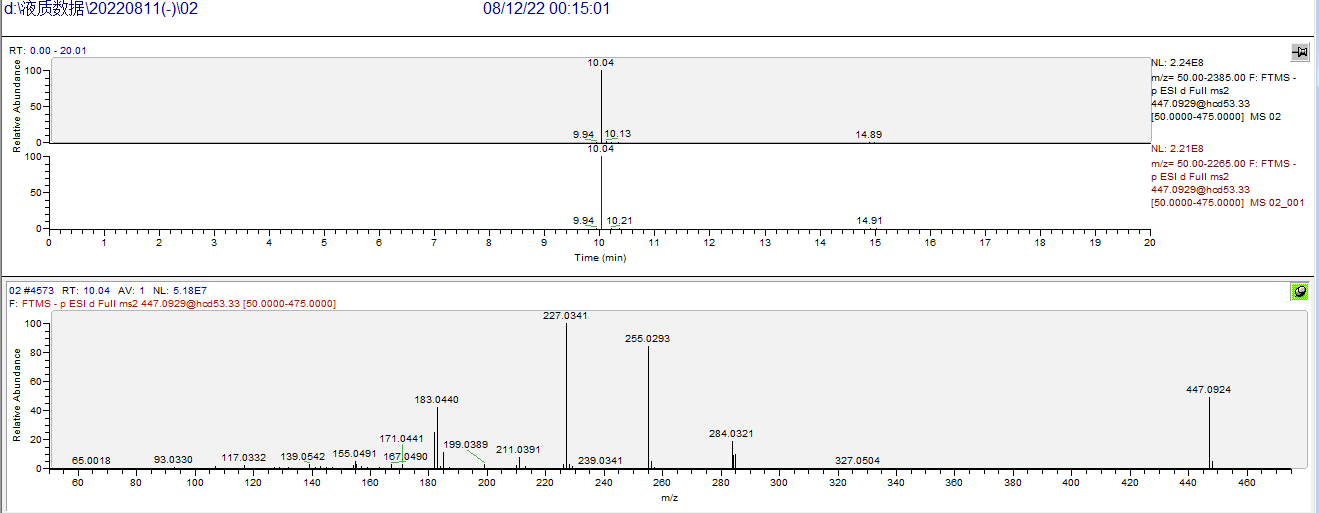


Sample


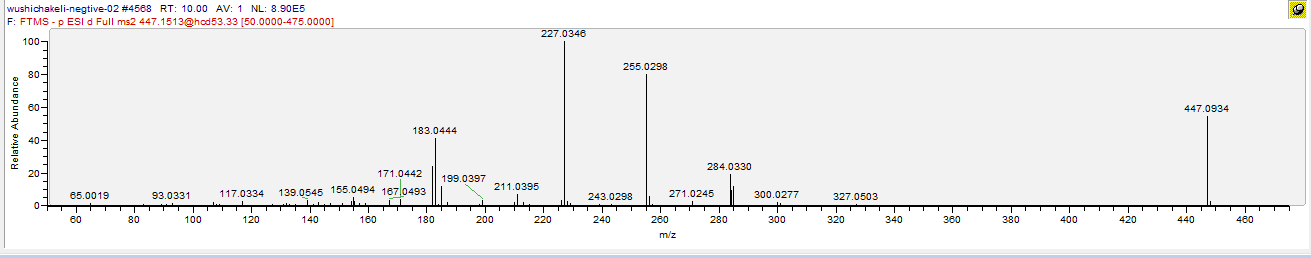

Supplement: Supplementary file 25 — Additional file 25. Identification of Astragalin (Cas 480-10-4, C21H20O11, M.W. 448.38). [file 13020_2023_829_MOESM25_ESM.docx]

Additional file 26. Identification of Isorhamnetin-3-o-β-d-glucoside (Cas 5041-82-7 , C22H22O12, M.W. 478.4)

Standard


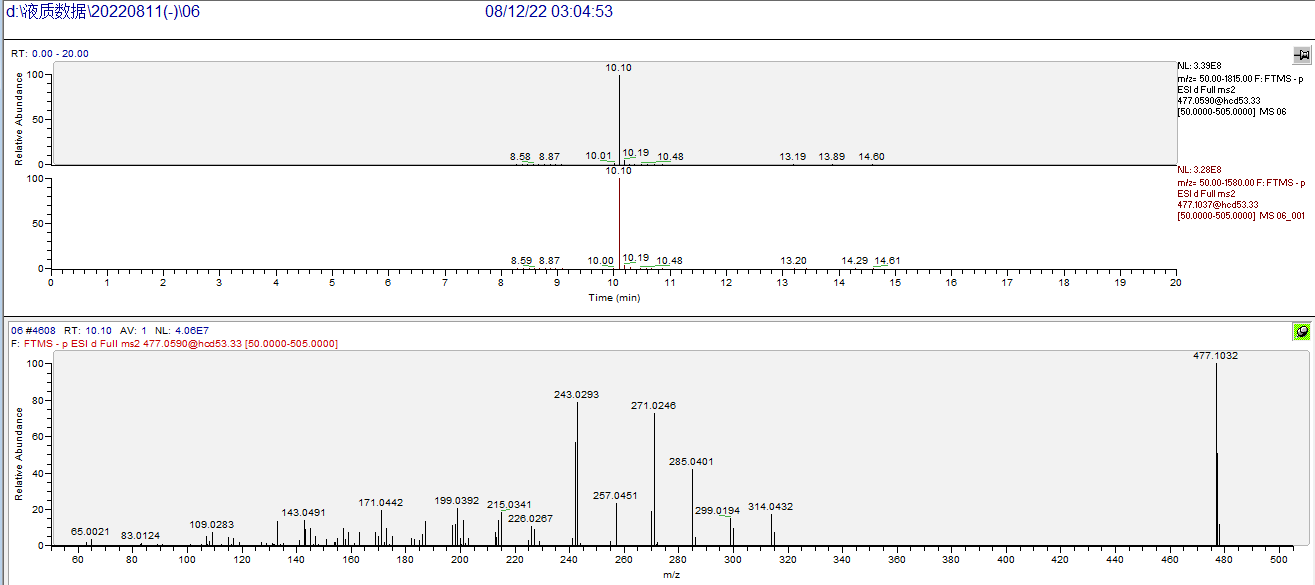


Sample


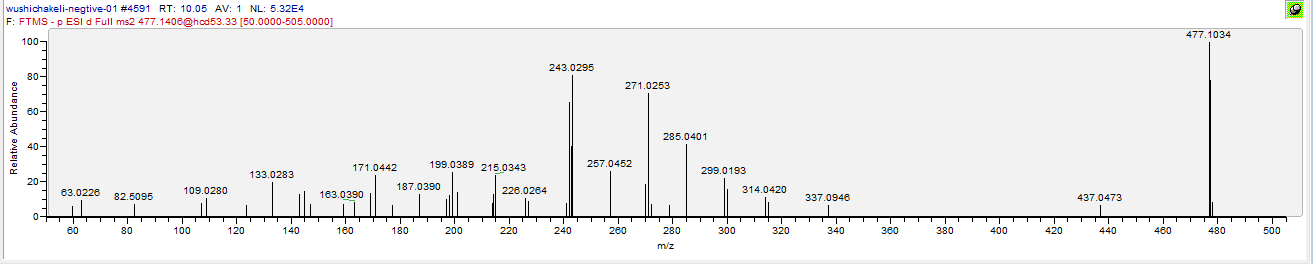

Supplement: Supplementary file 26 — Additional file 26. Identification of Isorhamnetin-3-o-β-d-glucoside (Cas 5041-82-7, C22H22O12, M.W. 478.4). [file 13020_2023_829_MOESM26_ESM.docx]

Additional file 27. Identification of Saikosaponin A (Cas 20736-09-8, C42H68O13, M.W. 780.982)

Standard


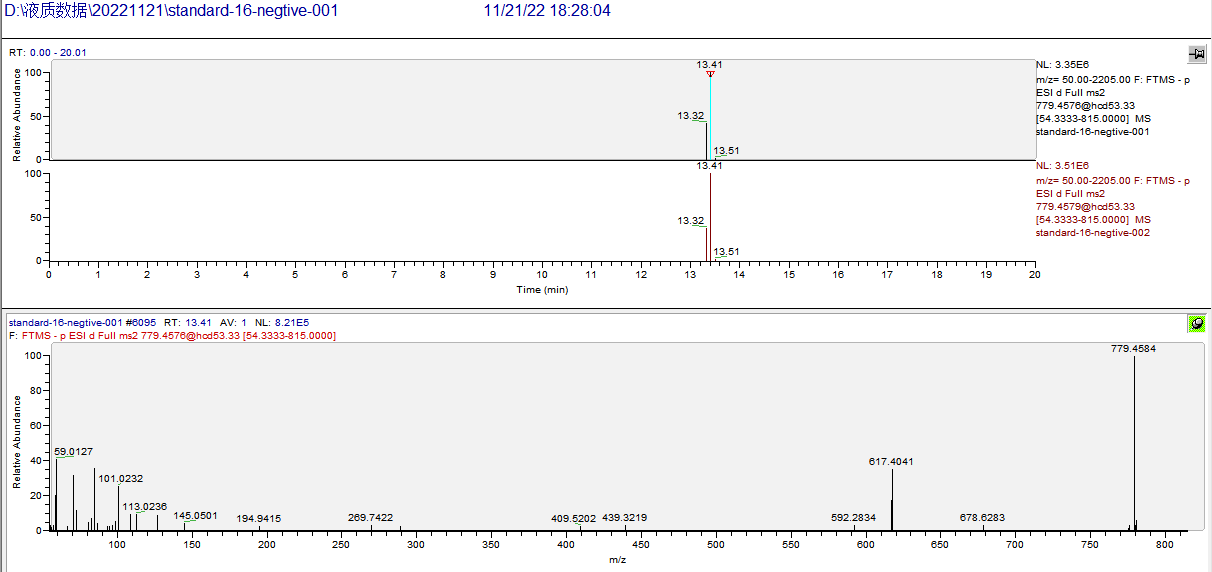


Sample


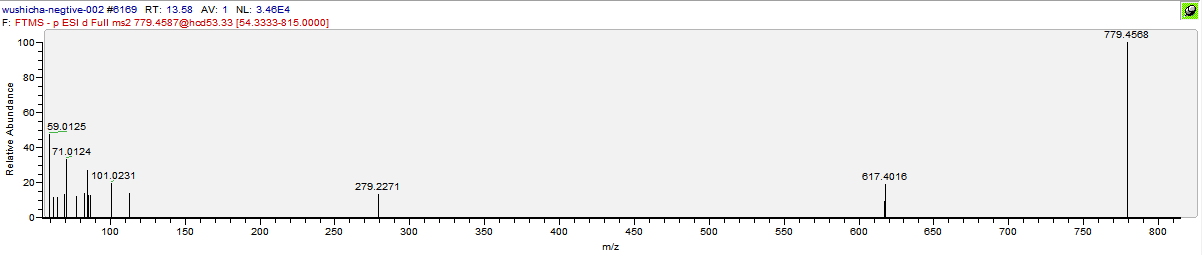

Supplement: Supplementary file 27 — Additional file 27. Identification of Saikosaponin A (Cas 20736-09-8, C42H68O13, M.W. 780.982). [file 13020_2023_829_MOESM27_ESM.docx]

Additional file 28. Identification of Daidzein (Cas 486-66-8, C15H10O4, M.W. 254.24)

Standard


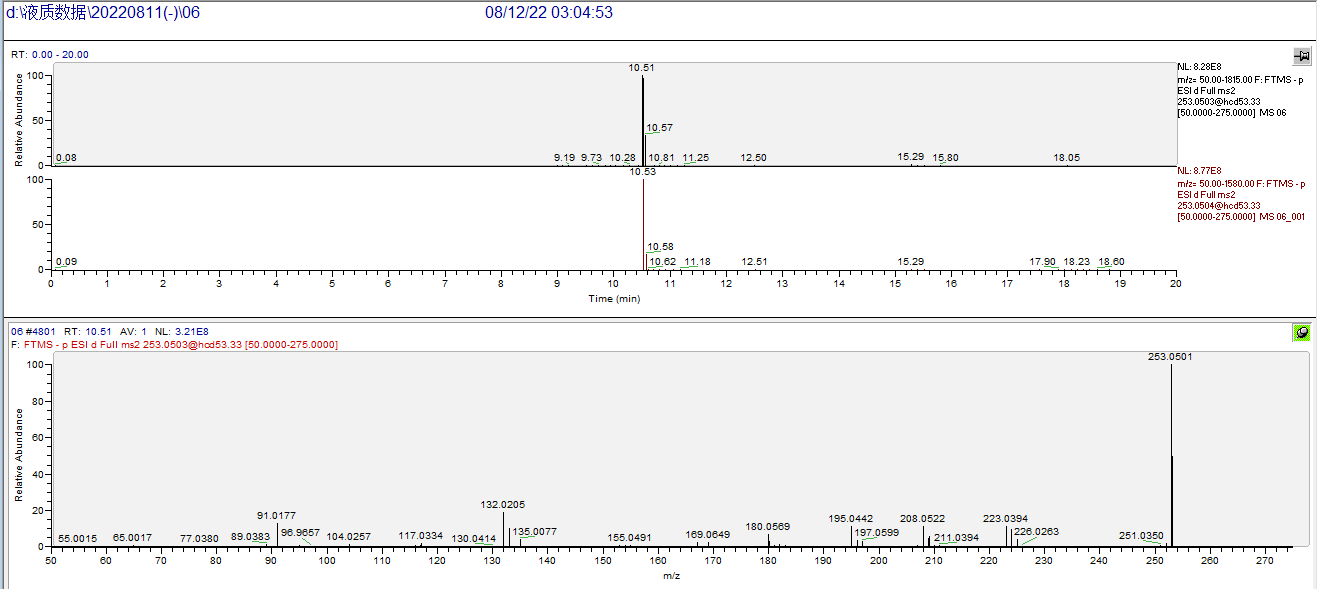


Sample


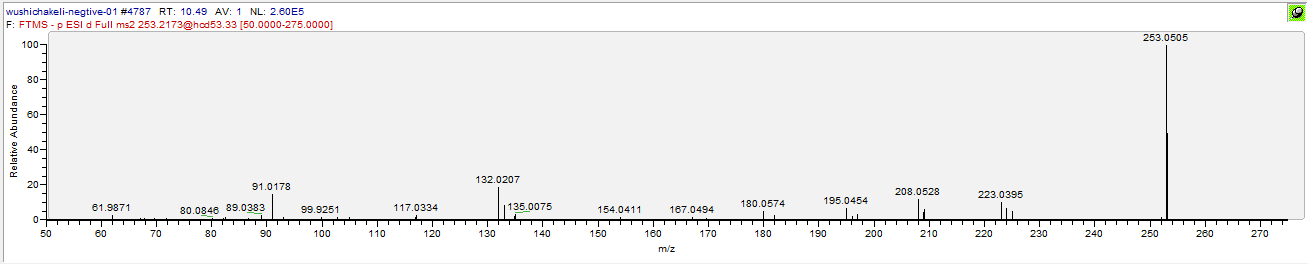

Supplement: Supplementary file 28 — Additional file 28. Identification of Daidzein (Cas 486-66-8, C15H10O4, M.W. 254.24). [file 13020_2023_829_MOESM28_ESM.docx]

Additional file 29. Identification of Quercetin (Cas 117-39-5, C15H10O7, M.W. 302.23)

Standard


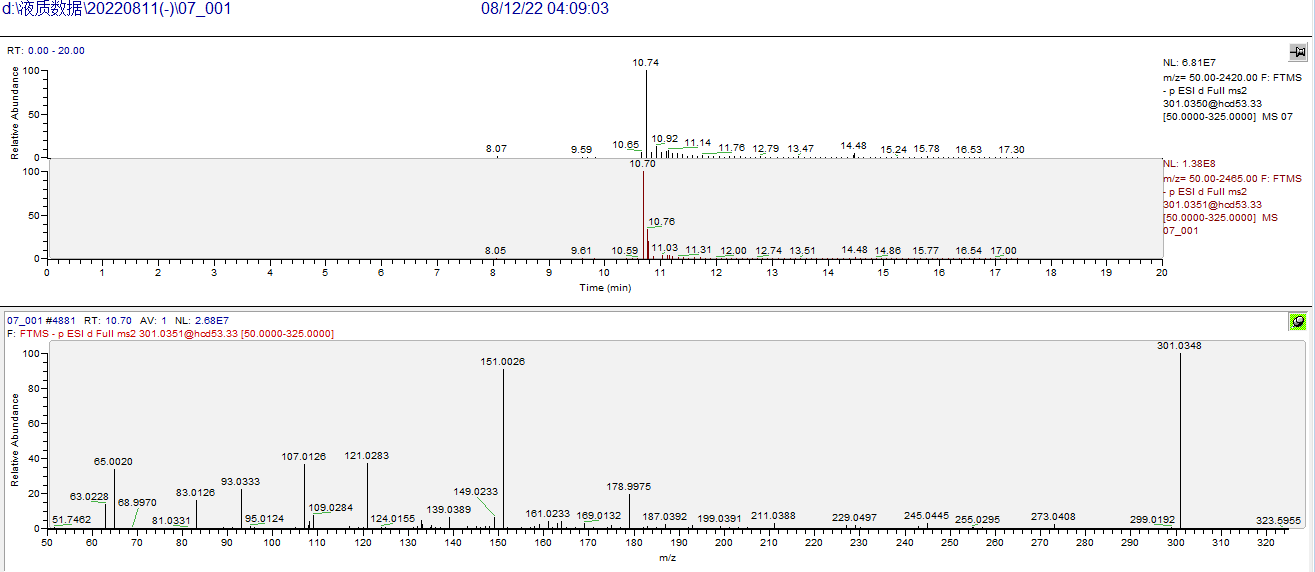


Sample


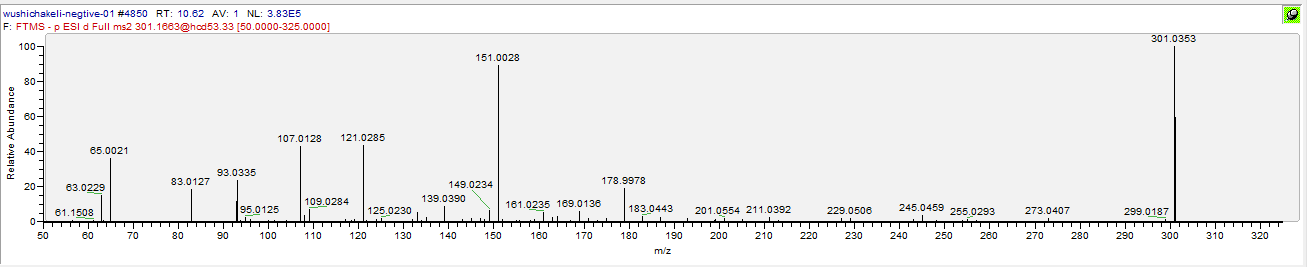

Supplement: Supplementary file 29 — Additional file 29. Identification of Quercetin (Cas 117-39-5, C15H10O7, M.W. 302.23). [file 13020_2023_829_MOESM29_ESM.docx]

Additional file 30. Identification of 7,4'-dihydroxyflavone (Cas 2196-14-7, C15H10O4, M.W. 254)

Standard


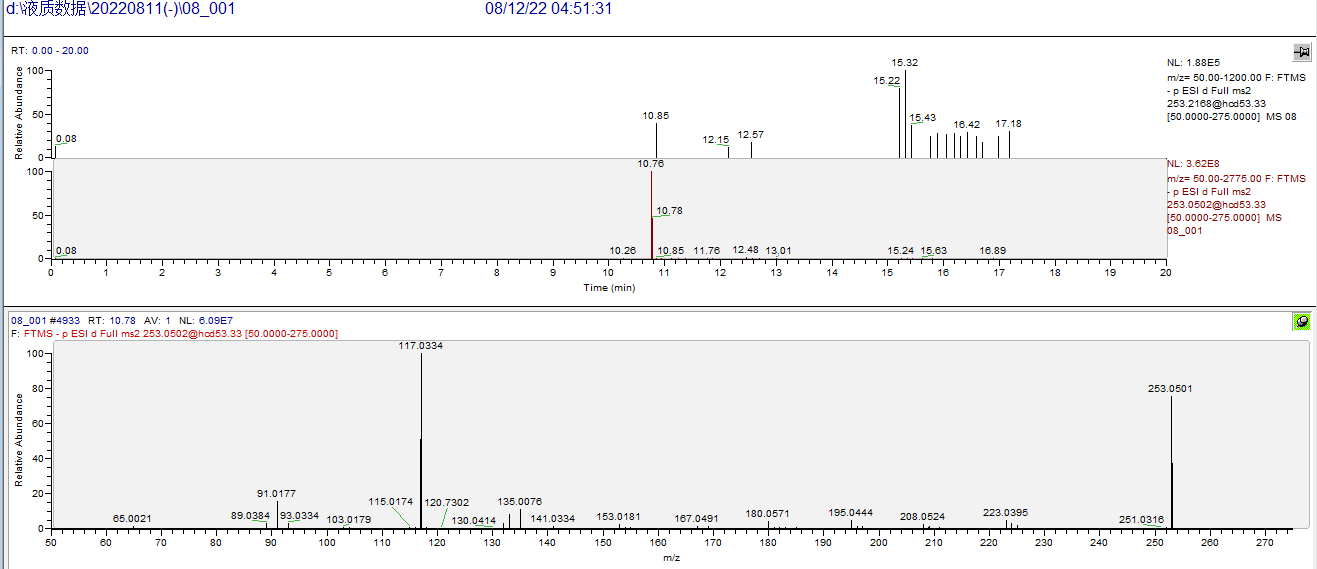


Sample


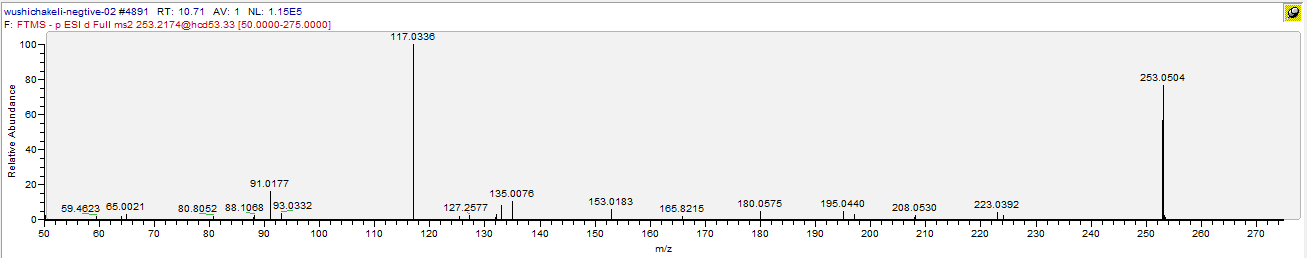

Supplement: Supplementary file 30 — Additional file 30. Identification of 7,4'-dihydroxyflavone (Cas 2196-14-7, C15H10O4, M.W. 254). [file 13020_2023_829_MOESM30_ESM.docx]

Additional file 31. Identification of S-Naringenin (Cas 480-41-1, C15H12O5, M.W.272.25)

Standard


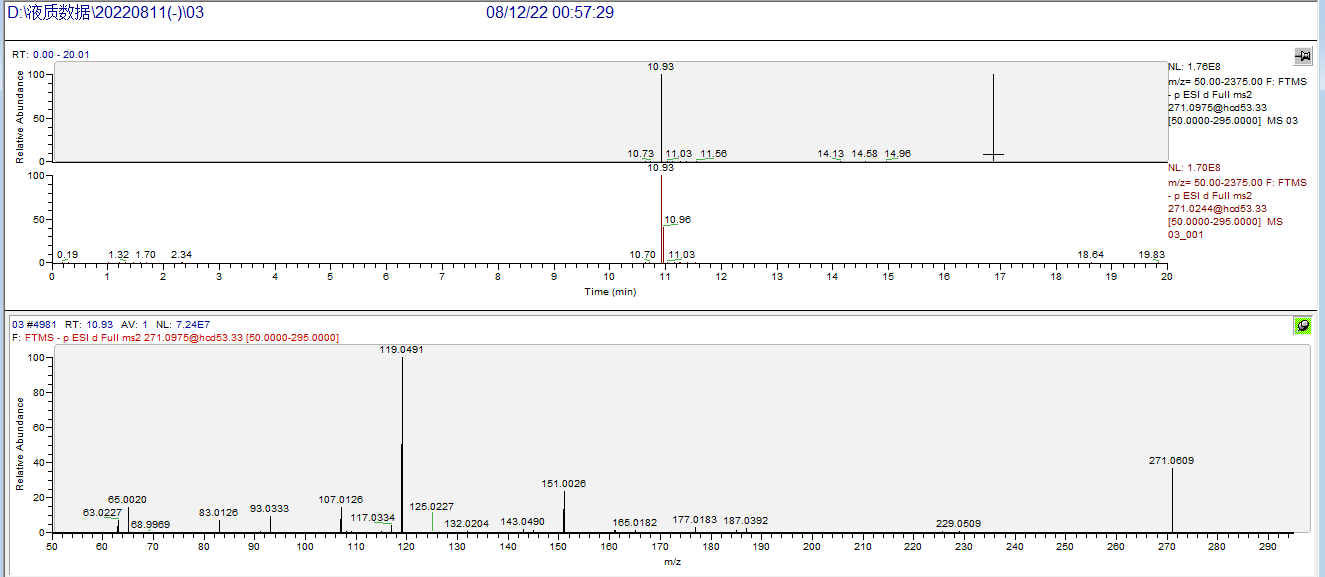


Sample


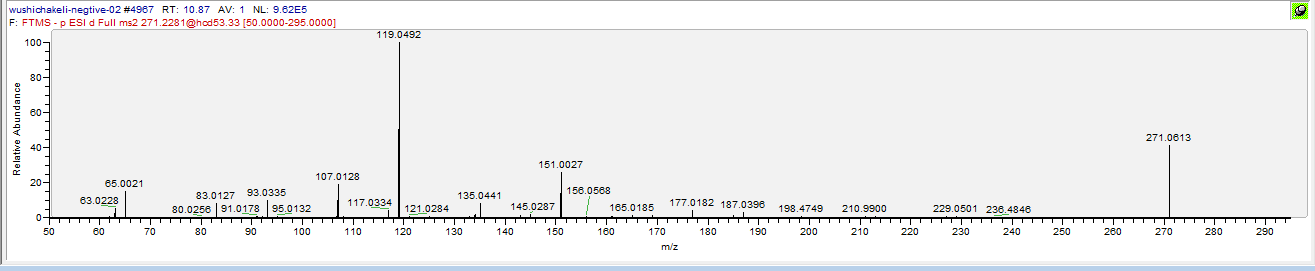

Supplement: Supplementary file 31 — Additional file 31. Identification of S-Naringenin (Cas 480-41-1, C15H12O5, M.W.272.25). [file 13020_2023_829_MOESM31_ESM.docx]

Additional file 32. Identification of naringenin chalcone (Cas 73692-50-9, C15H12O5, M.W. 272.25)

Standard


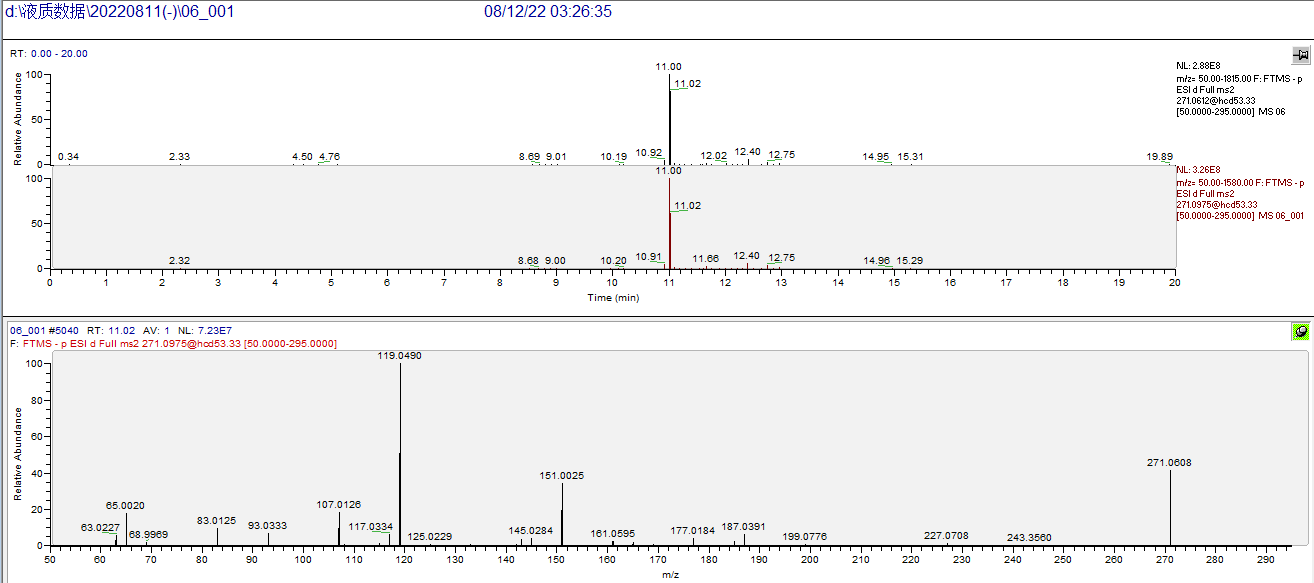


Sample


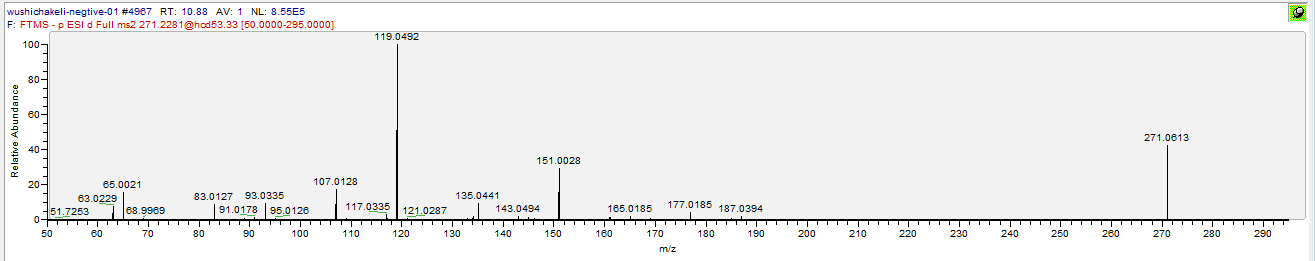

Supplement: Supplementary file 32 — Additional file 32. Identification of naringenin chalcone (Cas 73692-50-9, C15H12O5, M.W. 272.25). [file 13020_2023_829_MOESM32_ESM.docx]

Additional file 33. Identification of Luteolin (Cas 491-70-3, C15H10O6, M.W.286.24)

Standard


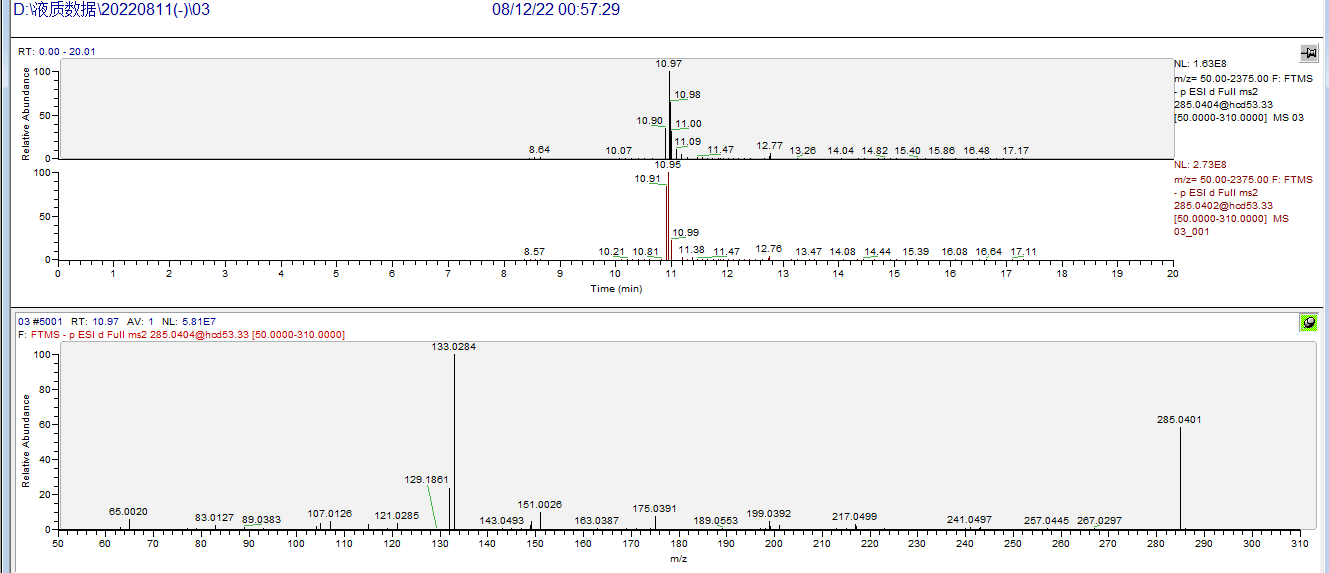


Sample


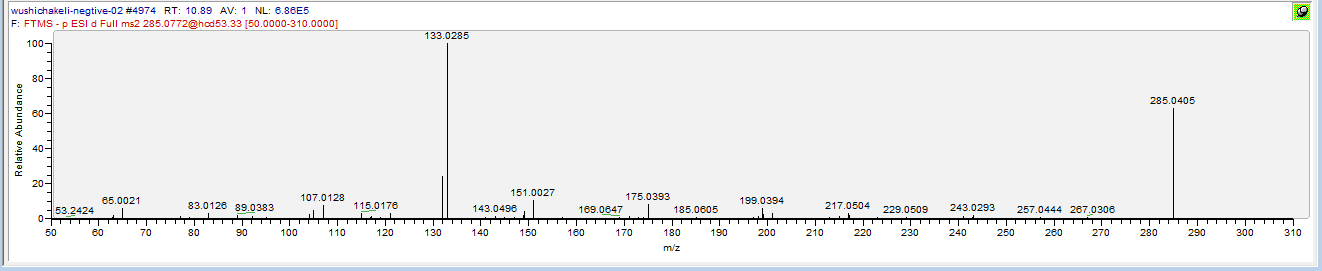

Supplement: Supplementary file 33 — Additional file 33. Identification of Luteolin (Cas 491-70-3, C15H10O6, M.W.286.24). [file 13020_2023_829_MOESM33_ESM.docx]

Additional file 34. Identification of Hesperetin (Cas 520-33-2, C16H14O6, M.W. 302.28)

Standard


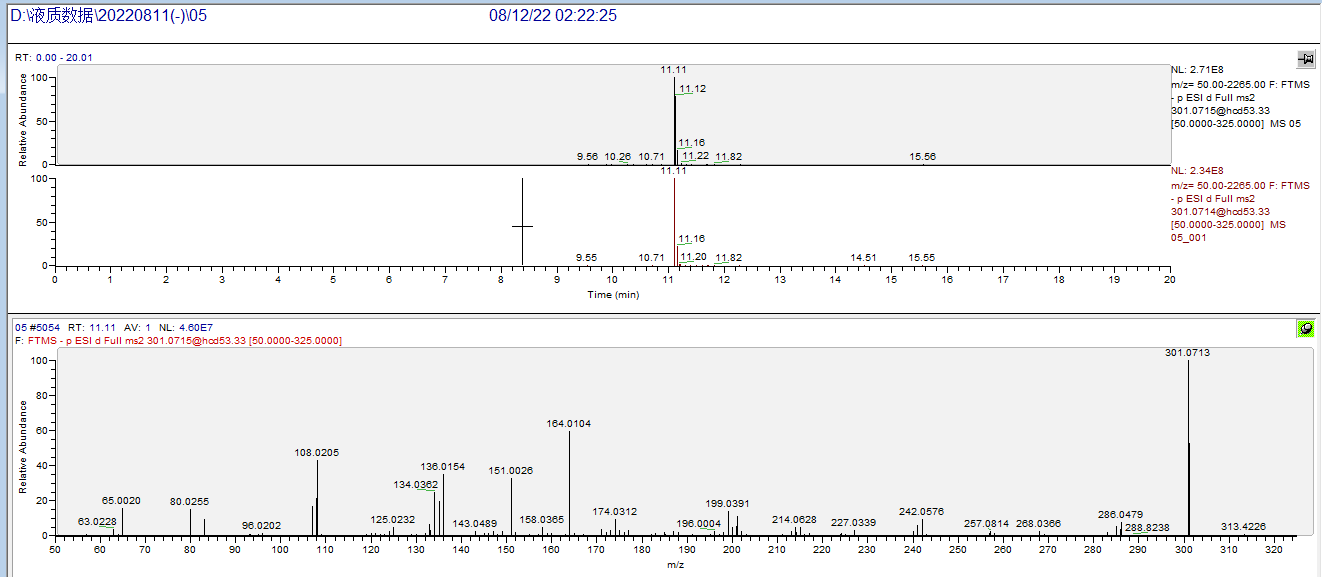


Sample


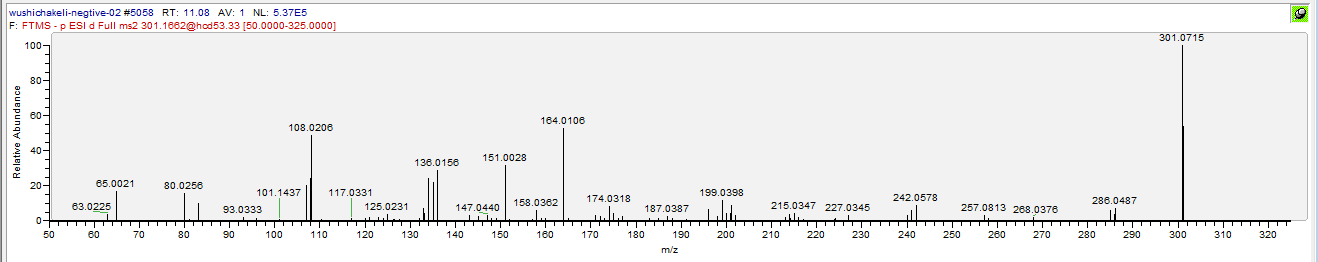

Supplement: Supplementary file 34 — Additional file 34. Identification of Hesperetin (Cas 520-33-2, C16H14O6, M.W. 302.28). [file 13020_2023_829_MOESM34_ESM.docx]

Additional file 35. Identification of Randaiol (Cas 87562-14-9, C15H14O3, M.W. 242.27)

Standard


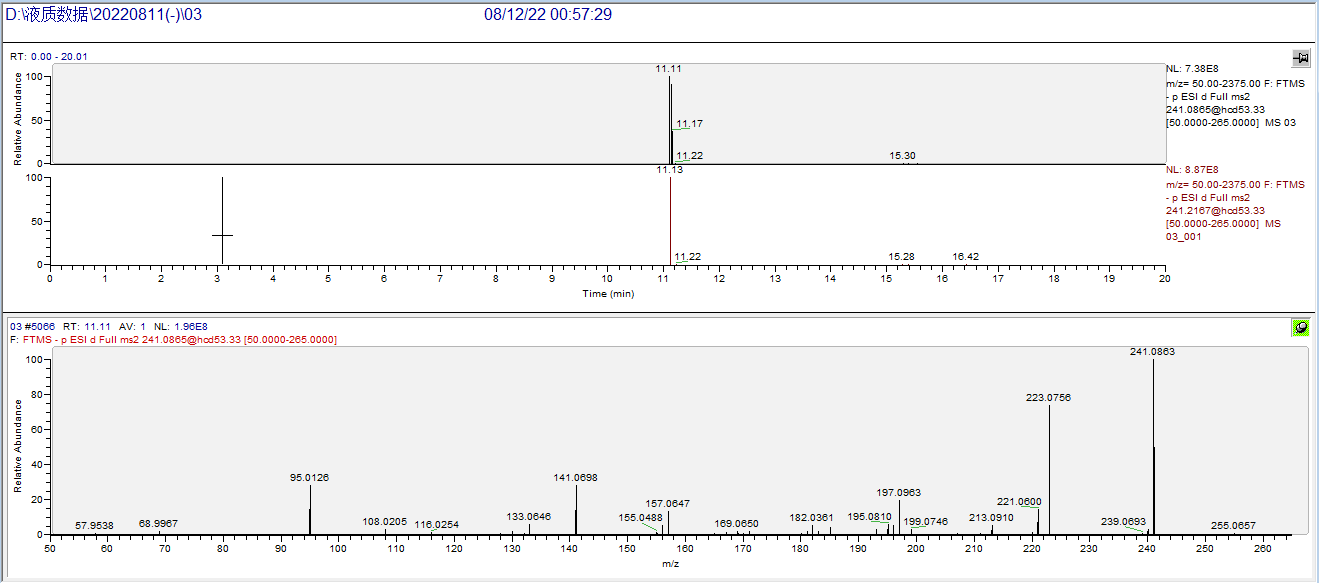


Sample


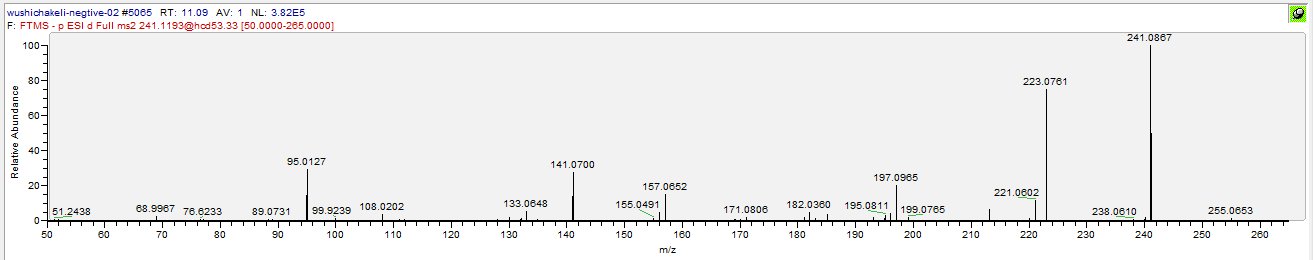

Supplement: Supplementary file 35 — Additional file 35. Identification of Randaiol (Cas 87562-14-9, C15H14O3, M.W. 242.27). [file 13020_2023_829_MOESM35_ESM.docx]

Additional file 36. Identification of Isoliquiritigenin (Cas 961-29-5, C15H12O4, M.W. 256.257)

Standard


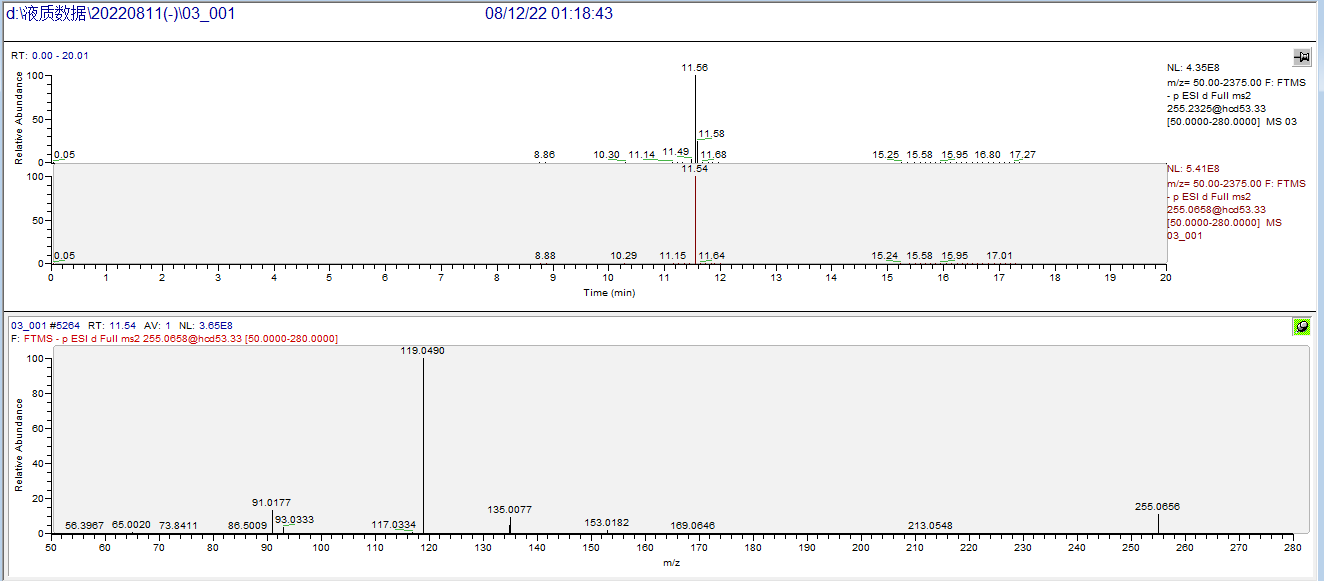


Sample


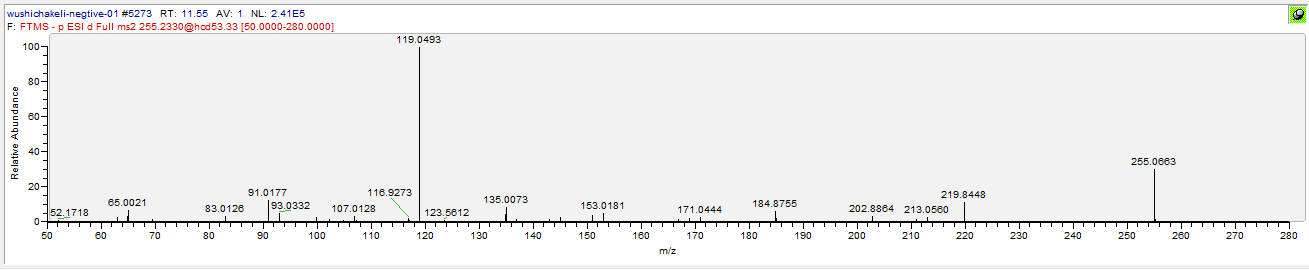

Supplement: Supplementary file 36 — Additional file 36. Identification of Isoliquiritigenin (Cas 961-29-5, C15H12O4, M.W. 256.257). [file 13020_2023_829_MOESM36_ESM.docx]

Additional file 37. Identification of Platycodin D (Cas 58479-68-8, C57H92O28, M.W. 1225.3)

Standard


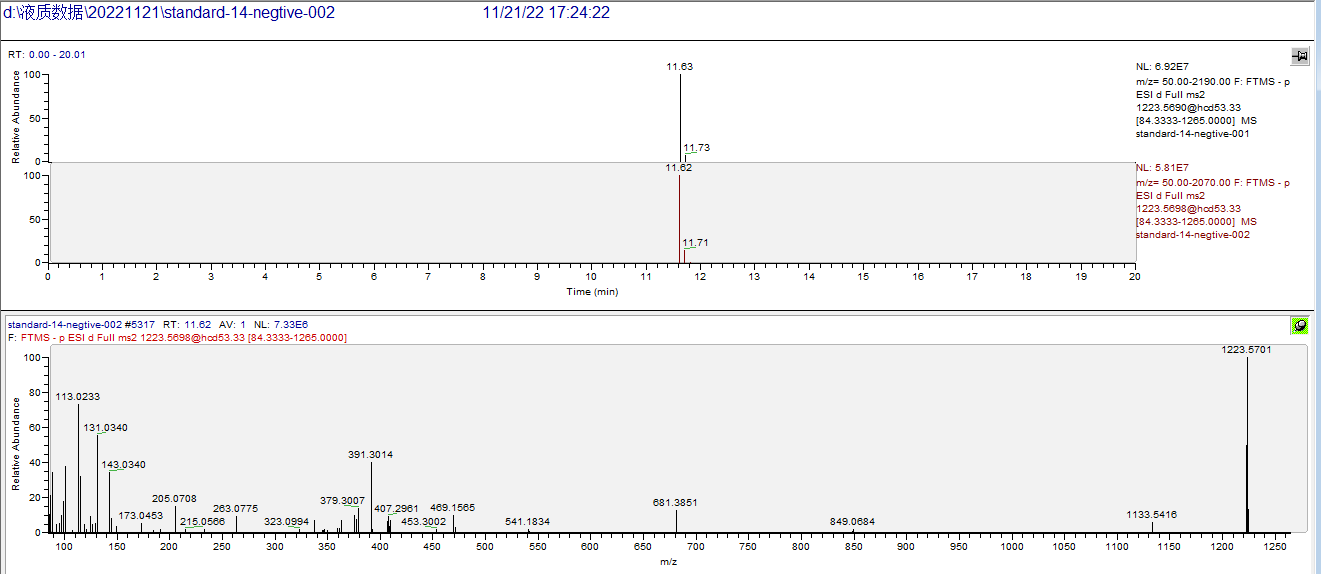


Sample


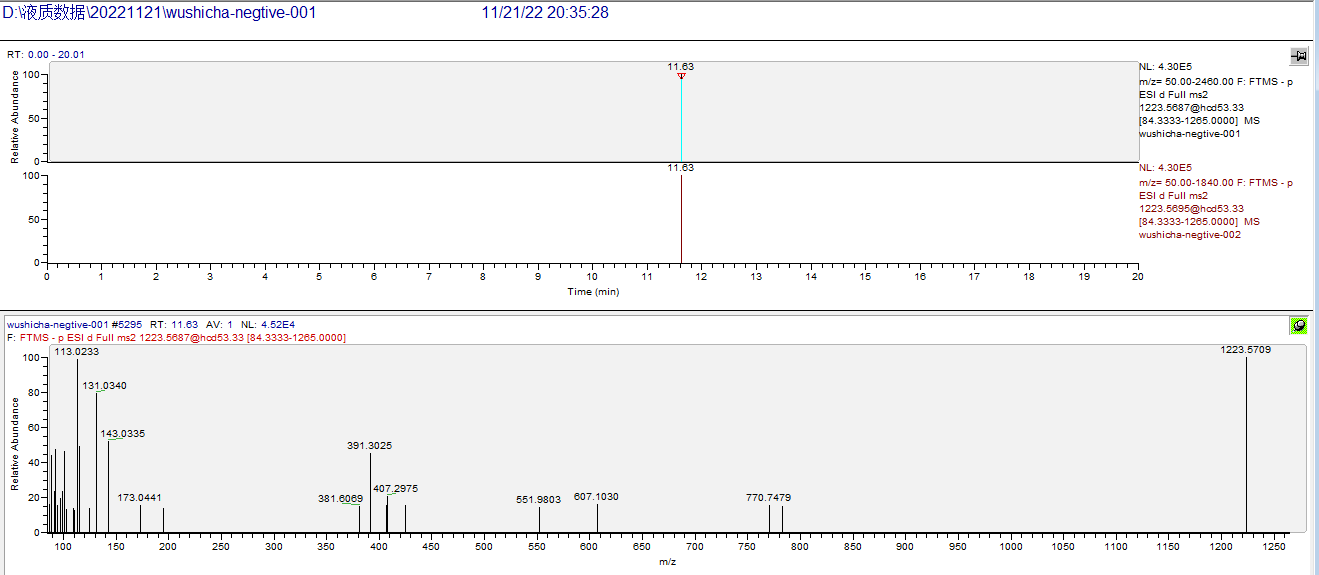

Supplement: Supplementary file 37 — Additional file 37. Identification of Platycodin D (Cas 58479-68-8, C57H92O28, M.W. 1225.3). [file 13020_2023_829_MOESM37_ESM.docx]

Additional file 38. Identification of Formononetin (Cas 485-72-3, C16H12O4, M.W. 268.26)

Standard


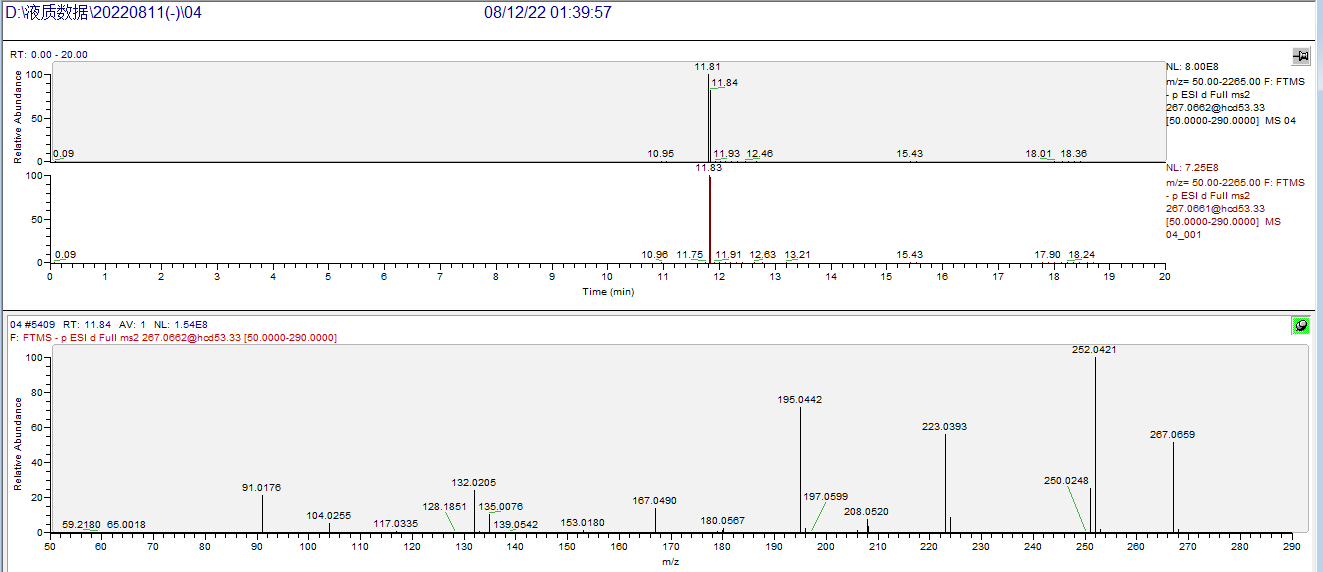


Sample


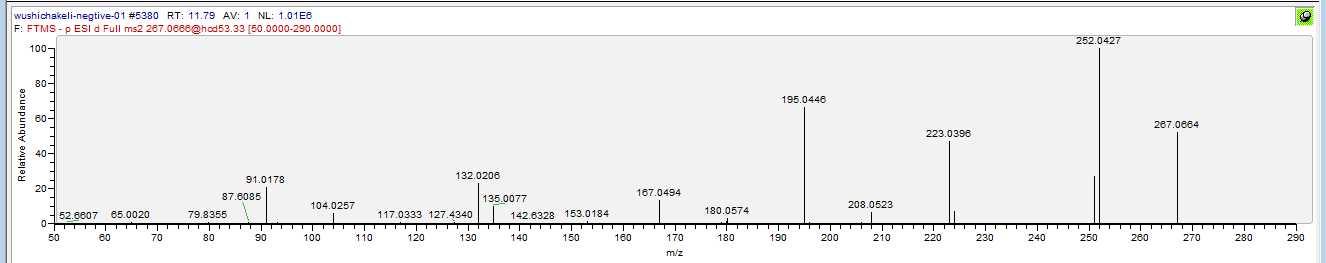

Supplement: Supplementary file 38 — Additional file 38. Identification of Formononetin (Cas 485-72-3, C16H12O4, M.W. 268.26). [file 13020_2023_829_MOESM38_ESM.docx]

Additional file 39. Identification of Senkyunolide A (Cas 63038-10-8, C12H16O2, M.W. 192.25)

Standard


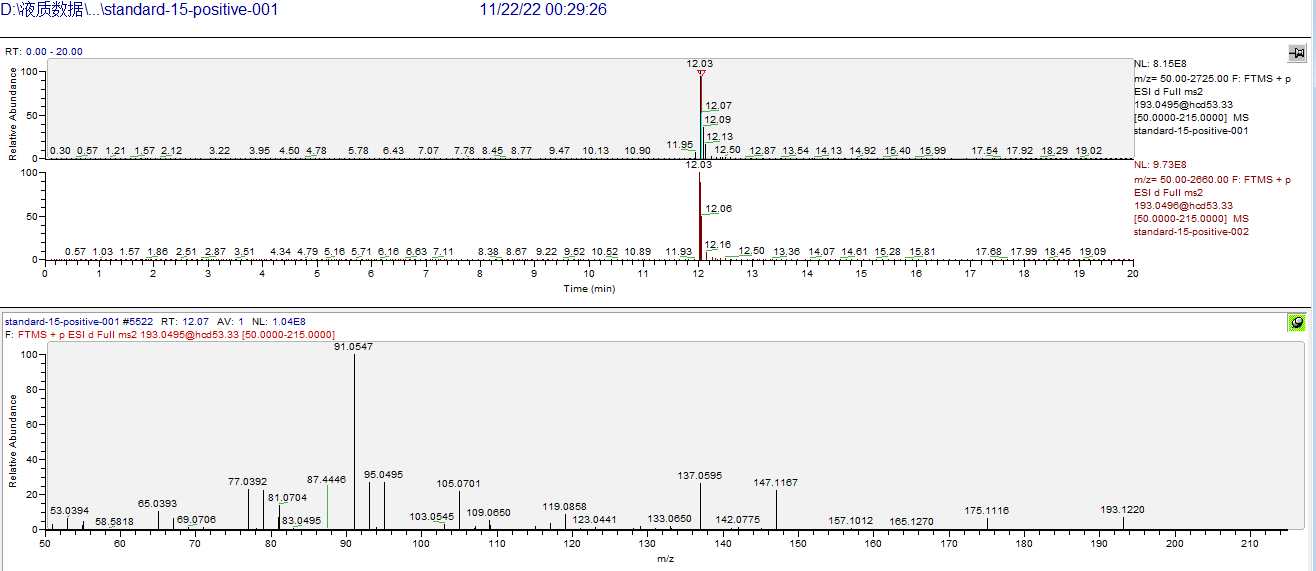


Sample


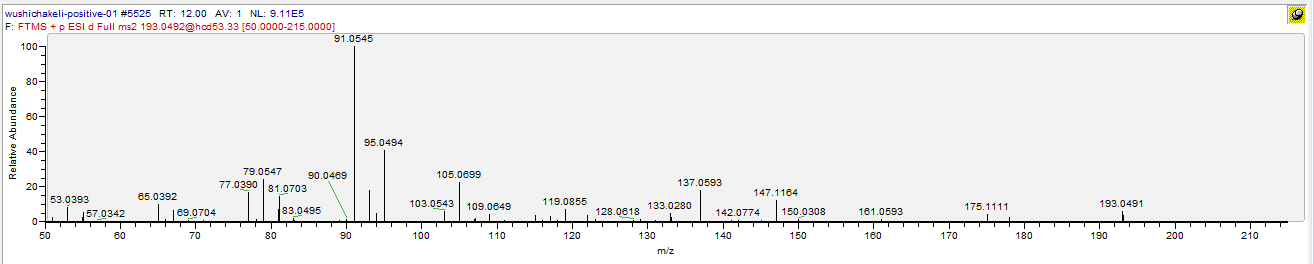

Supplement: Supplementary file 39 — Additional file 39. Identification of Senkyunolide A (Cas 63038-10-8, C12H16O2, M.W. 192.25). [file 13020_2023_829_MOESM39_ESM.docx]

Additional file 40. Identification of 3,5,6,7,8,3,4,-7-Methoxy-2-phenyl-4H-chromen-4-one (Cas 1178-24-1, C22H24O9, M.W. 432.421)

Standard


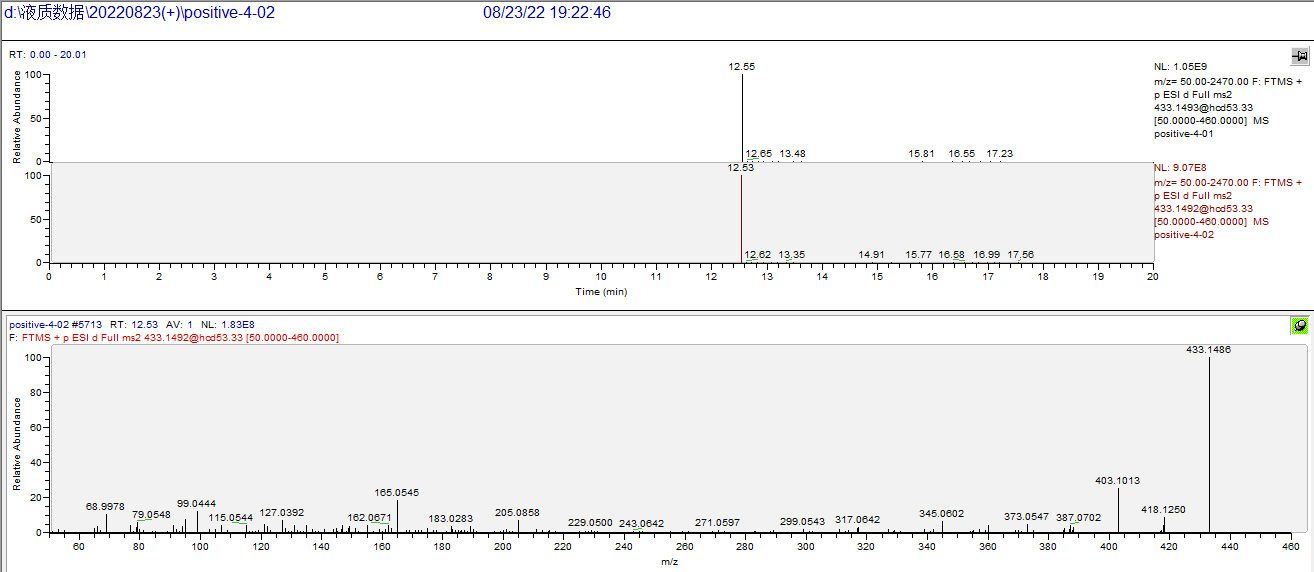


Sample


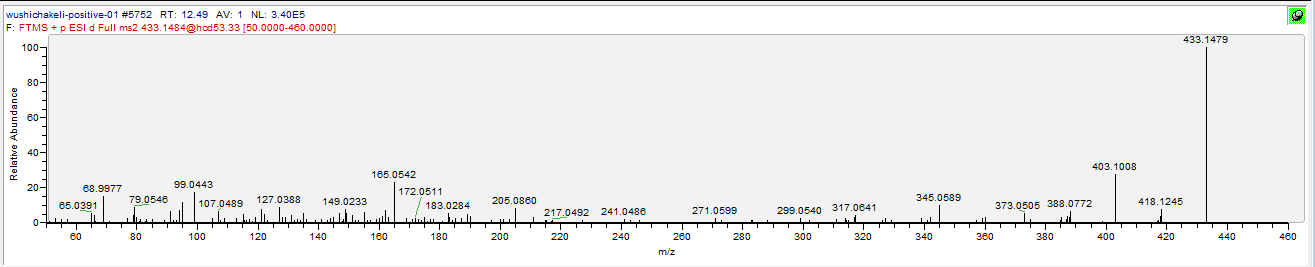

Supplement: Supplementary file 40 — Additional file 40. Identification of 3,5,6,7,8,3,4,-7-Methoxy-2-phenyl-4H-chromen-4-one (Cas 1178-24-1, C22H24O9, M.W. 432.421). [file 13020_2023_829_MOESM40_ESM.docx]

Additional file 41. Identification of Licoricesaponin H2 (Cas 118441-85-3, C42H62O16, M.W. 822.93)

Standard


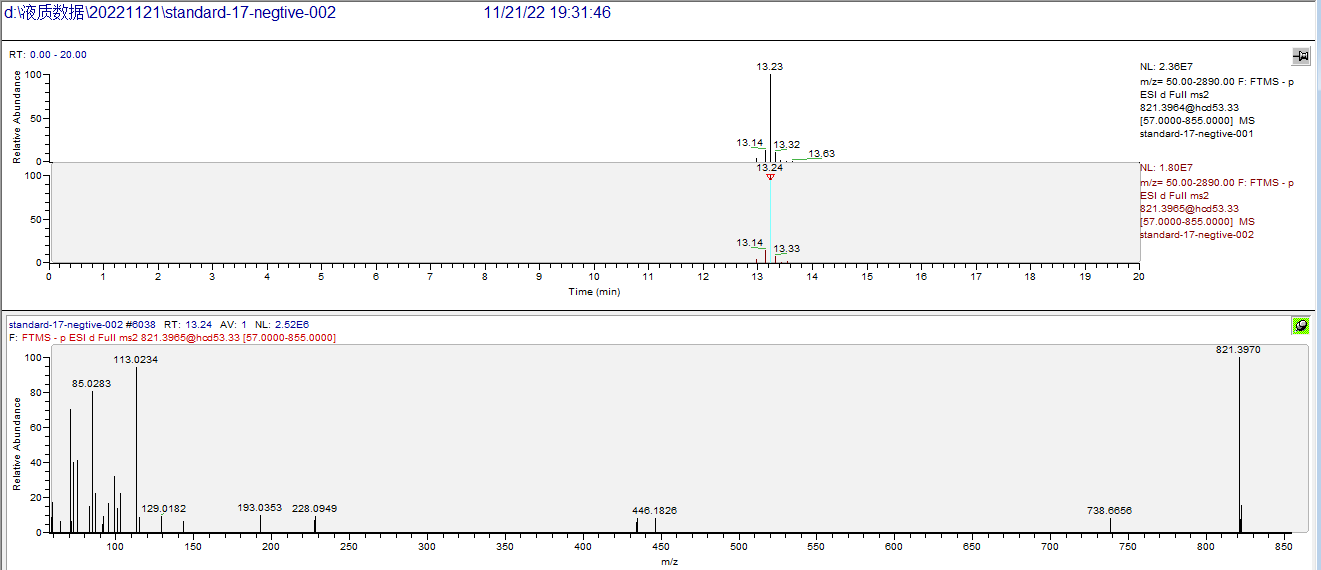


Sample


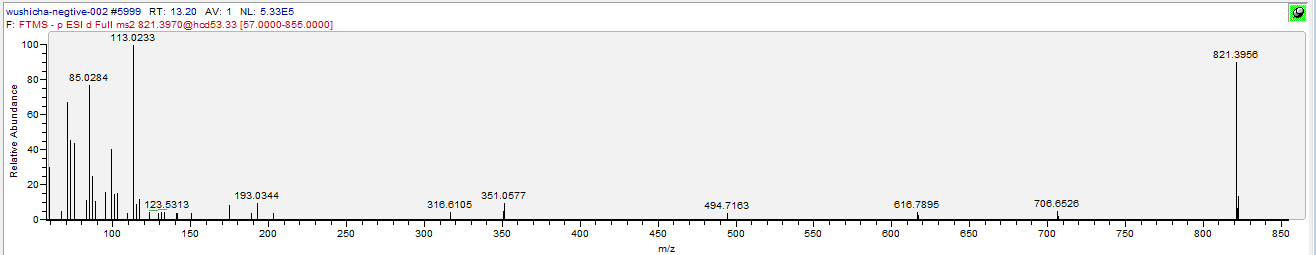

Supplement: Supplementary file 41 — Additional file 41. Identification of Licoricesaponin H2 (Cas 118441-85-3, C42H62O16, M.W. 822.93). [file 13020_2023_829_MOESM41_ESM.docx]

Additional file 42. Identification of 5-Hydroxyflavone (Cas 491-78-1, C15H10O3, M.W. 238.24)

Standard


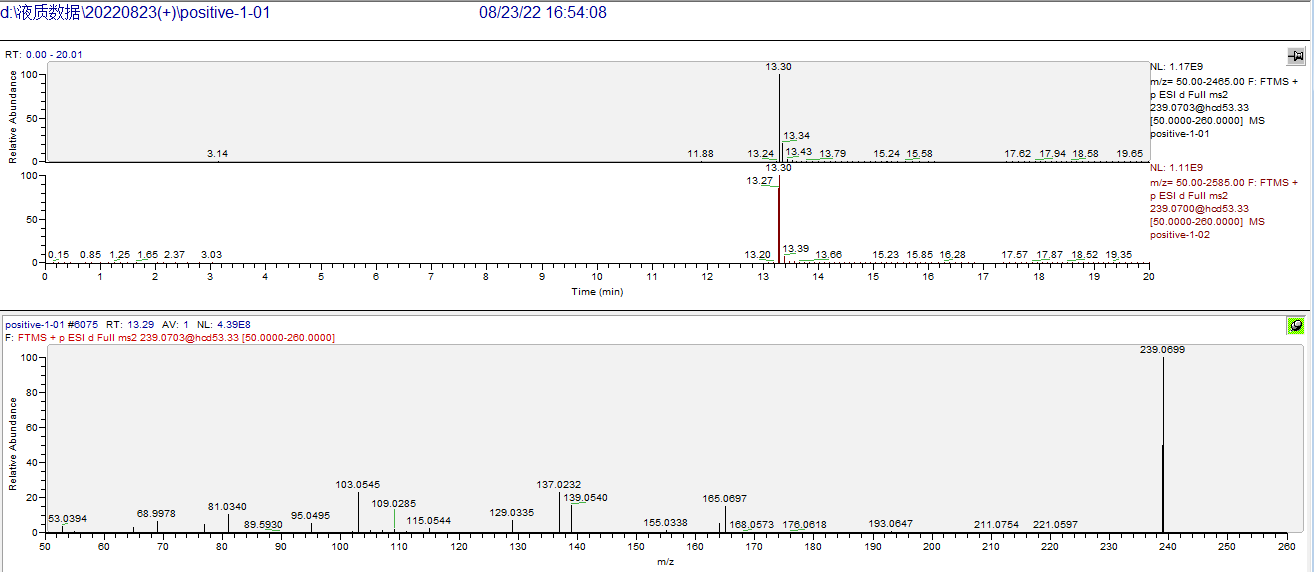


Sample


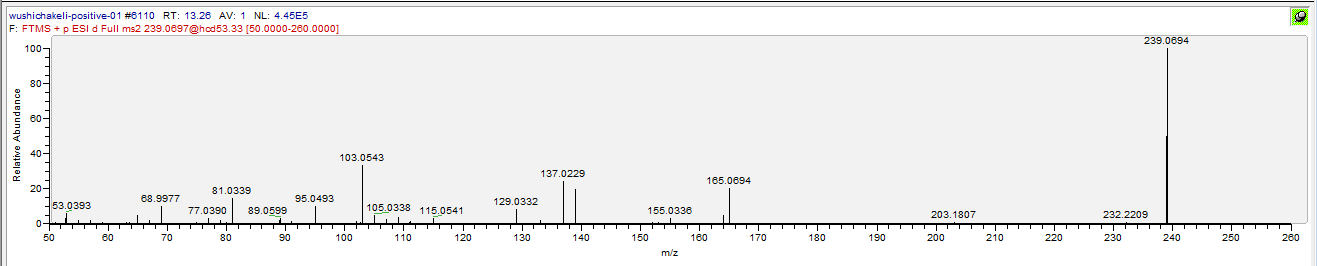

Supplement: Supplementary file 42 — Additional file 42. Identification of 5-Hydroxyflavone (Cas 491-78-1, C15H10O3, M.W. 238.24). [file 13020_2023_829_MOESM42_ESM.docx]

Additional file 43. Identification of Magnolol (Cas 528-43-8, C18H18O2, M.W. 266.32)

Standard


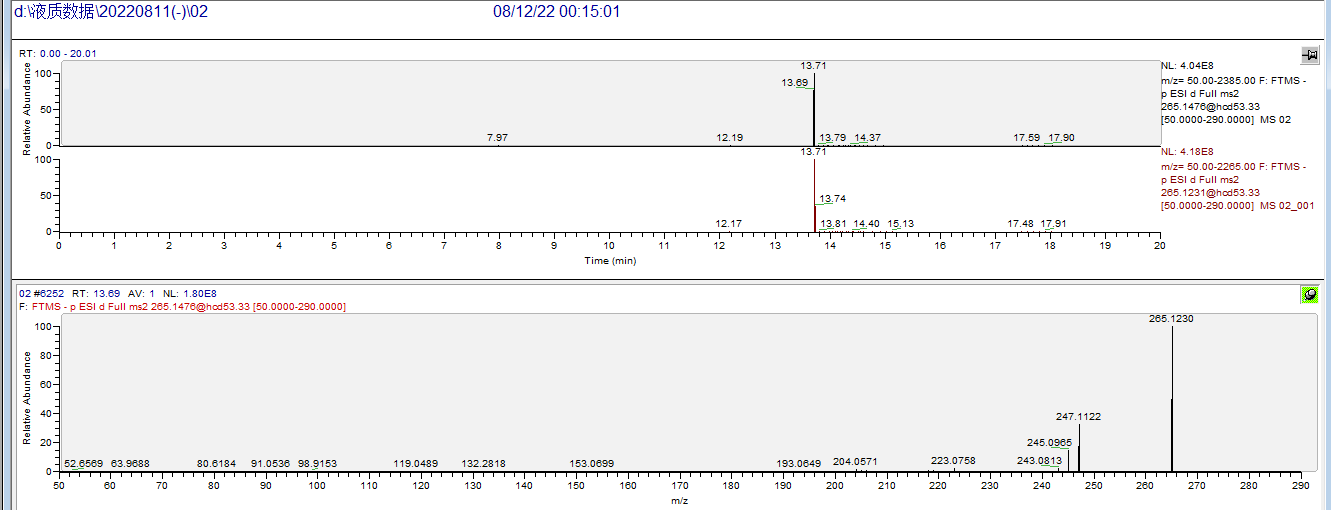


Sample


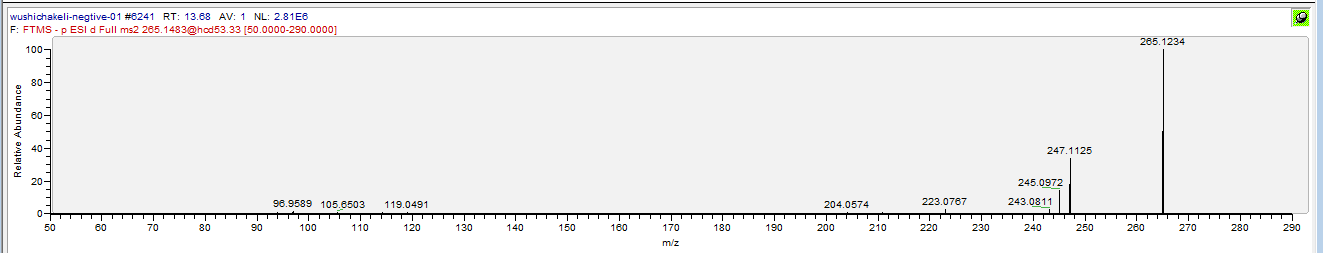

Supplement: Supplementary file 43 — Additional file 43. Identification of Magnolol (Cas 528-43-8, C18H18O2, M.W. 266.32). [file 13020_2023_829_MOESM43_ESM.docx]

Additional file 45. Identification of linoleic acid (Cas 60-33-3, C18H32O2, M.W. 280.4)

Standard


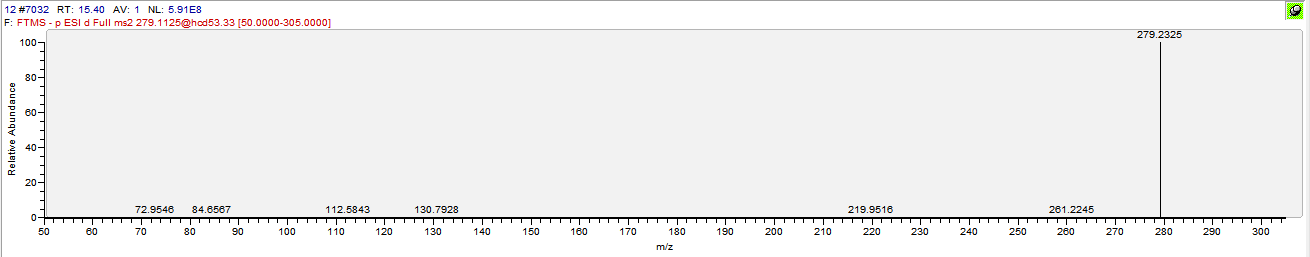


Sample


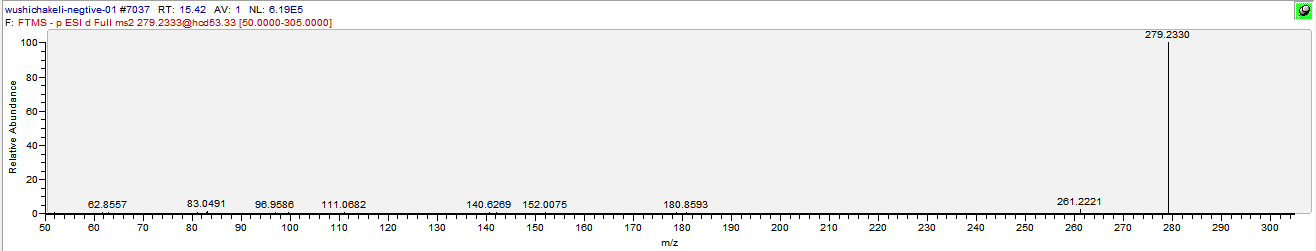

Supplement: Supplementary file 45 — Additional file 45. Identification of linoleic acid (Cas 60-33-3, C18H32O2, M.W. 280.4). [file 13020_2023_829_MOESM45_ESM.docx]

Additional file 46. Identification of palmitic Acid (Cas 57-10-3, C16H32O2, M.W. 256.4)

Standard


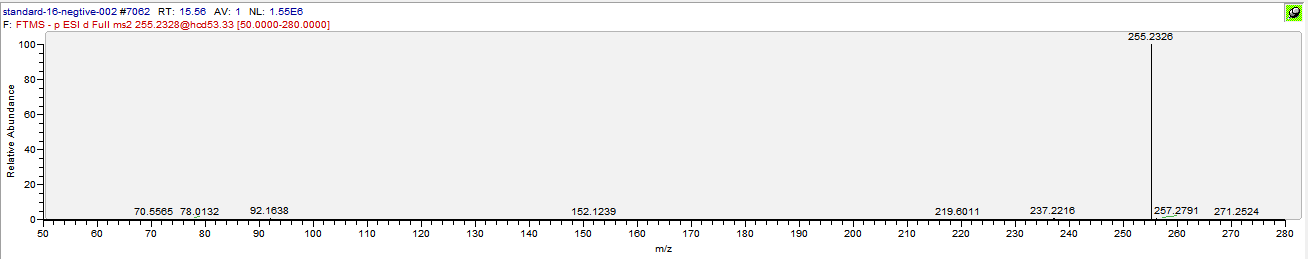


Sample


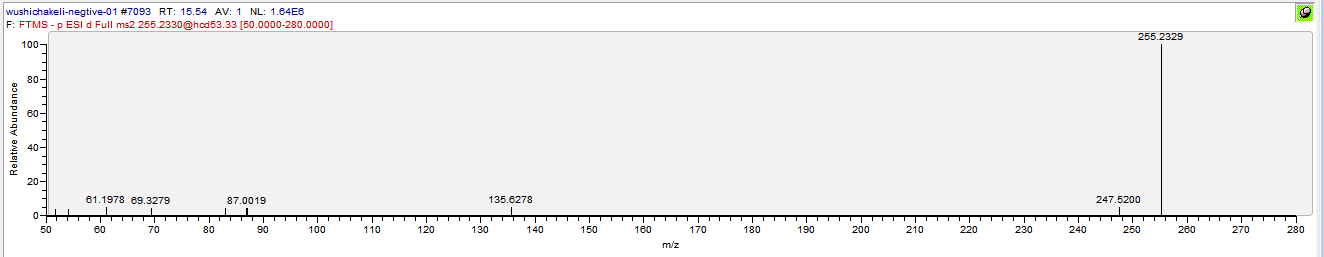

Supplement: Supplementary file 46 — Additional file 46. Identification of palmitic Acid (Cas 57-10-3, C16H32O2, M.W. 256.4). [file 13020_2023_829_MOESM46_ESM.docx]

Additional file 47. Identification of Oleic Acid (Cas 112-80-1, C18H34O2, M.W. 282.468)

Standard


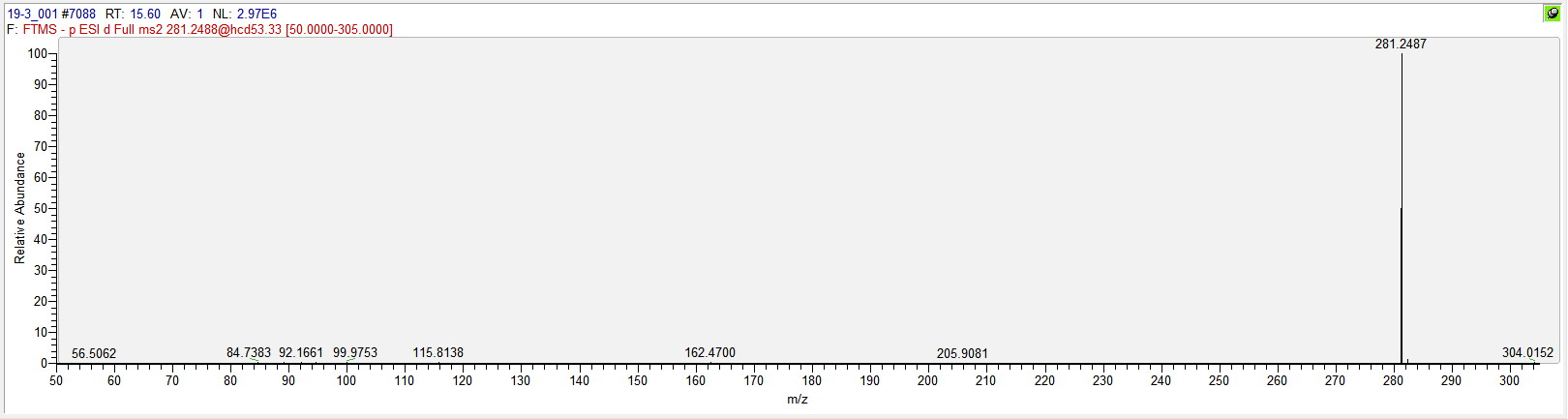


Sample


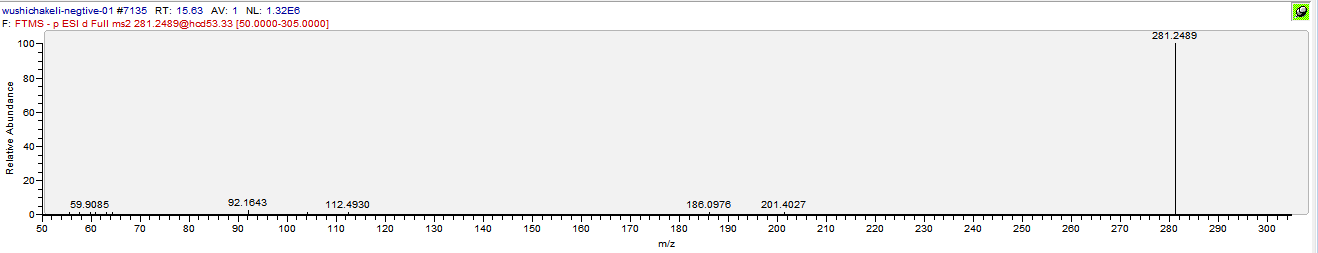

Supplement: Supplementary file 47 — Additional file 47. Identification of Oleic Acid (Cas 112-80-1, C18H34O2, M.W. 282.468). [file 13020_2023_829_MOESM47_ESM.docx]

Additional file 48. Identification of palmitic acid ethyl ester (Cas 628-97-7, C18H36O2, M.W. 284.484)

Standard


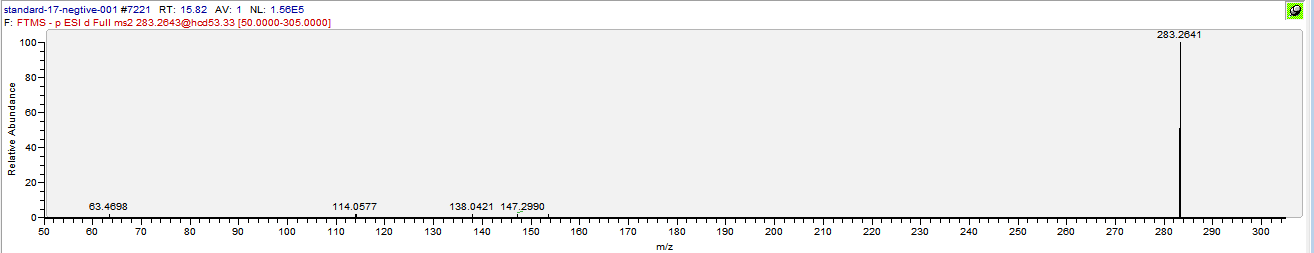


Sample


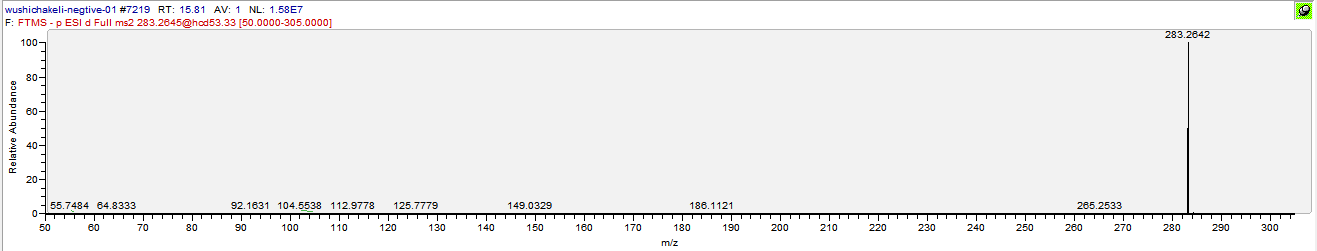

Supplement: Supplementary file 48 — Additional file 48. Identification of palmitic acid ethyl ester (Cas 628-97-7, C18H36O2, M.W. 284.484). [file 13020_2023_829_MOESM48_ESM.docx]

Additional file 49. Identification of Ethyl Stearate (Cas 111-61-5, C20H40O2, M.W. 312.53)

Standard


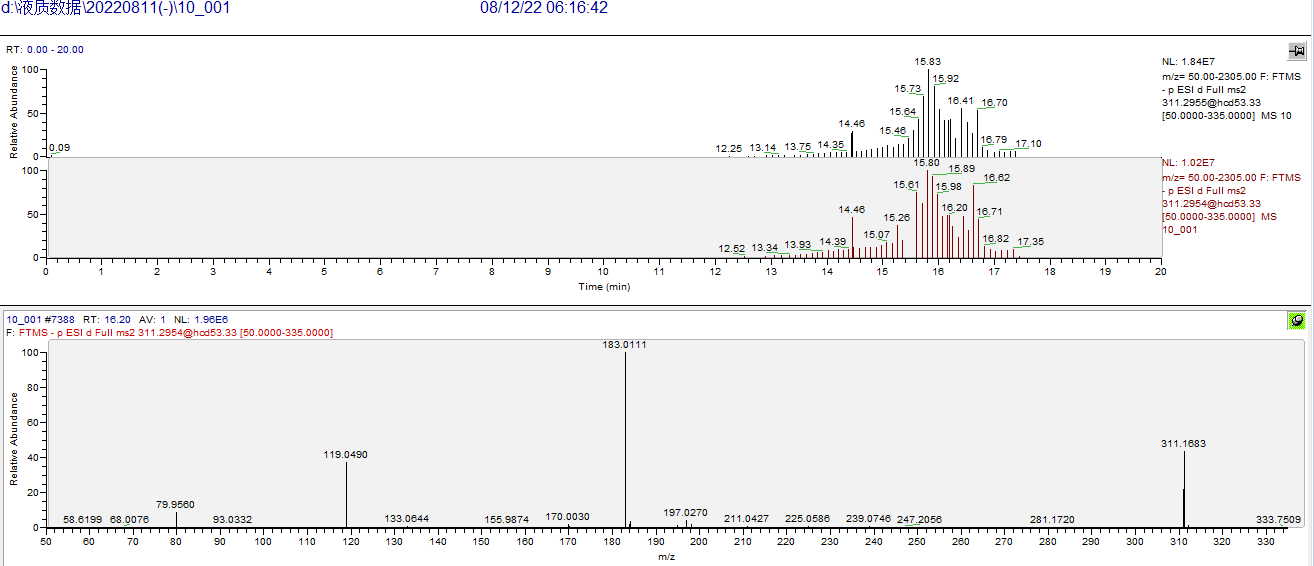


Sample


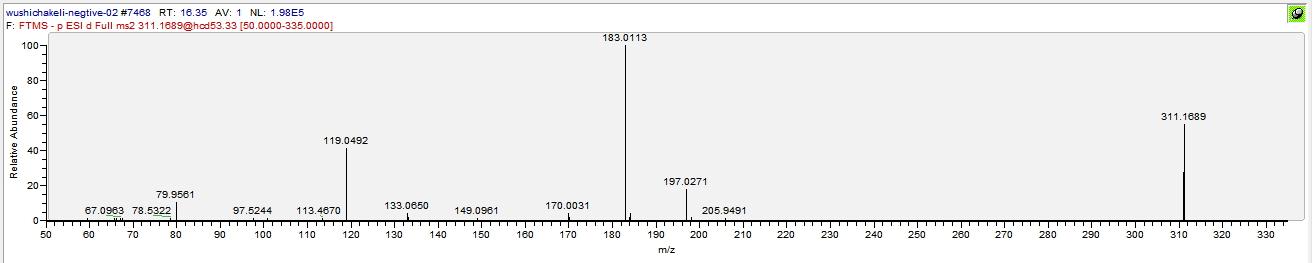

Supplement: Supplementary file 49 — Additional file 49. Identification of Ethyl Stearate (Cas 111-61-5, C20H40O2, M.W. 312.53). [file 13020_2023_829_MOESM49_ESM.docx]

Additional file 50. Identification of Hypericin (Cas 548-04-9, C30H16O8, M.W. 504.45)

Standard


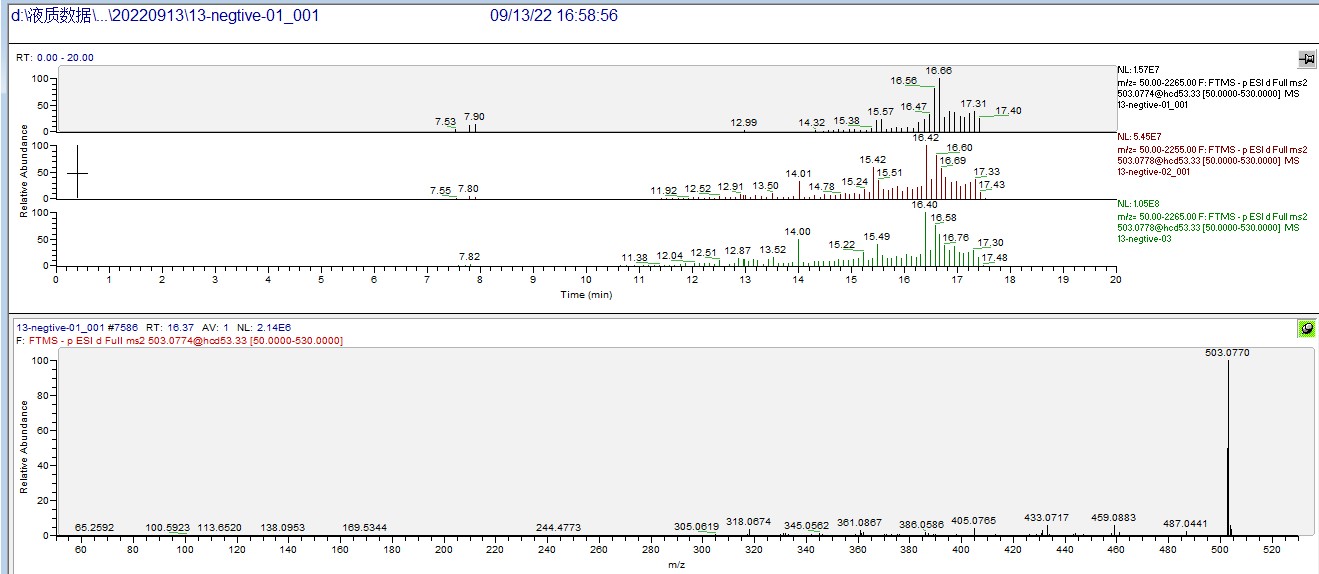


Sample


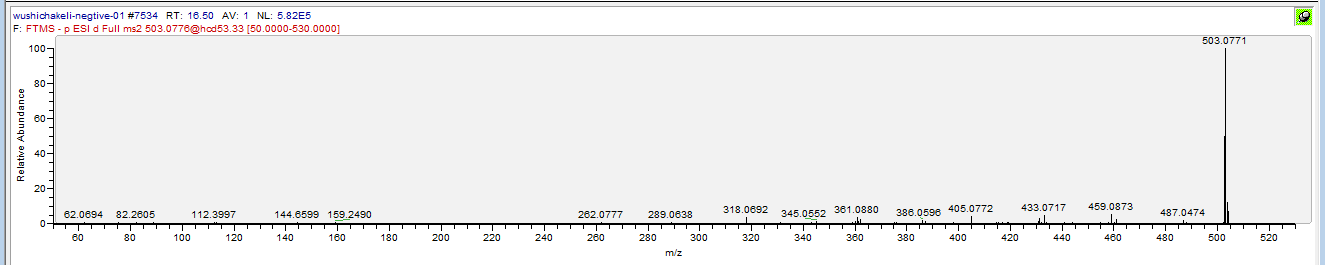

Supplement: Supplementary file 50 — Additional file 50. Identification of Hypericin (Cas 548-04-9, C30H16O8, M.W. 504.45). [file 13020_2023_829_MOESM50_ESM.docx]

Additional file 51. Identification of (+)-4-Cholesten-3-one (Cas 601-57-0, C27H44O, M.W. 384.65)

Standard
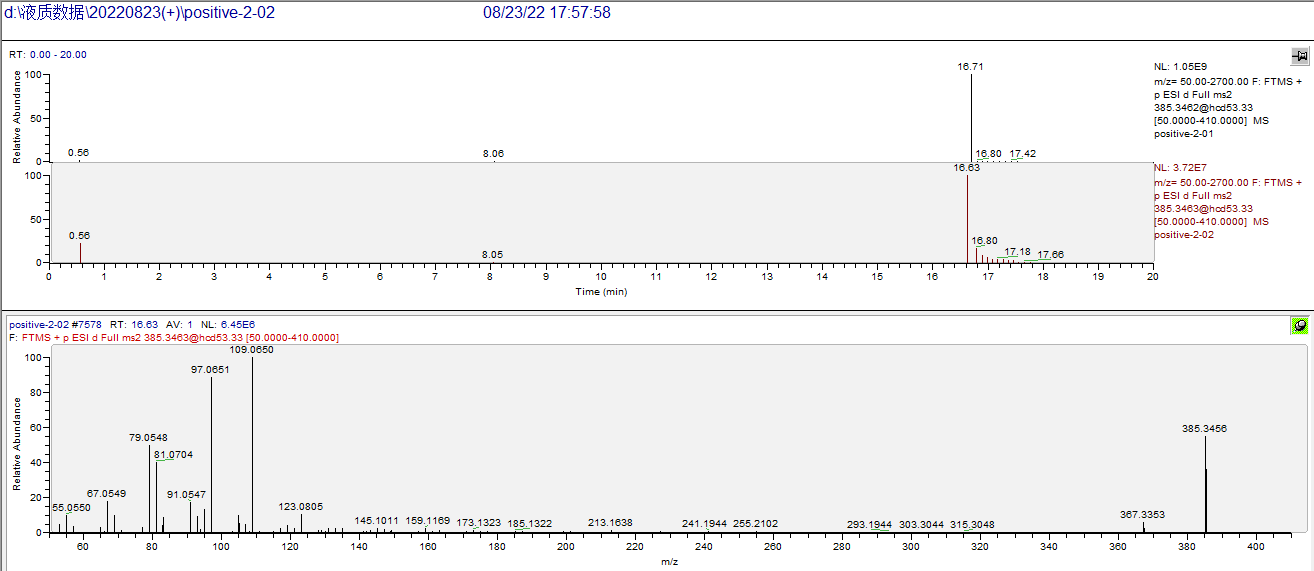


Sample
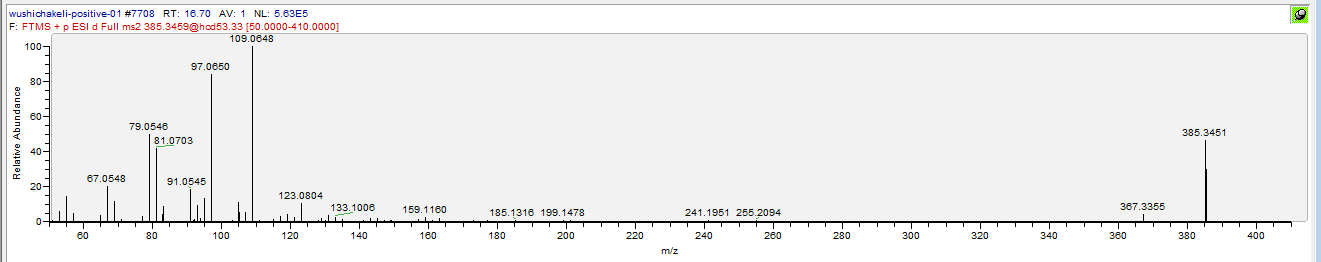

Supplement: Supplementary file 51 — Additional file 51. Identification of (+)-4-Cholesten-3-one (Cas 601-57-0, C27H44O, M.W. 384.65). [file 13020_2023_829_MOESM51_ESM.docx]
