# Supplementary material for: Database-aided UHPLC-Q-orbitrap MS/MS strategy putatively identifies 52 compounds from Wushicha Granule to propose anti-counterfeiting quality-markers for pharmacopoeia
Source: Chin Med. 2023 Sep 9;18:116. doi: 10.1186/s13020-023-00829-2 (PMC10492348; doi:10.1186/s13020-023-00829-2)

Additional file 44. Identification of 18β-glycyrrhetinic acid and exclusion of 18α- glycyrrhetinic acid

Standard 18α- glycyrrhetinic acid


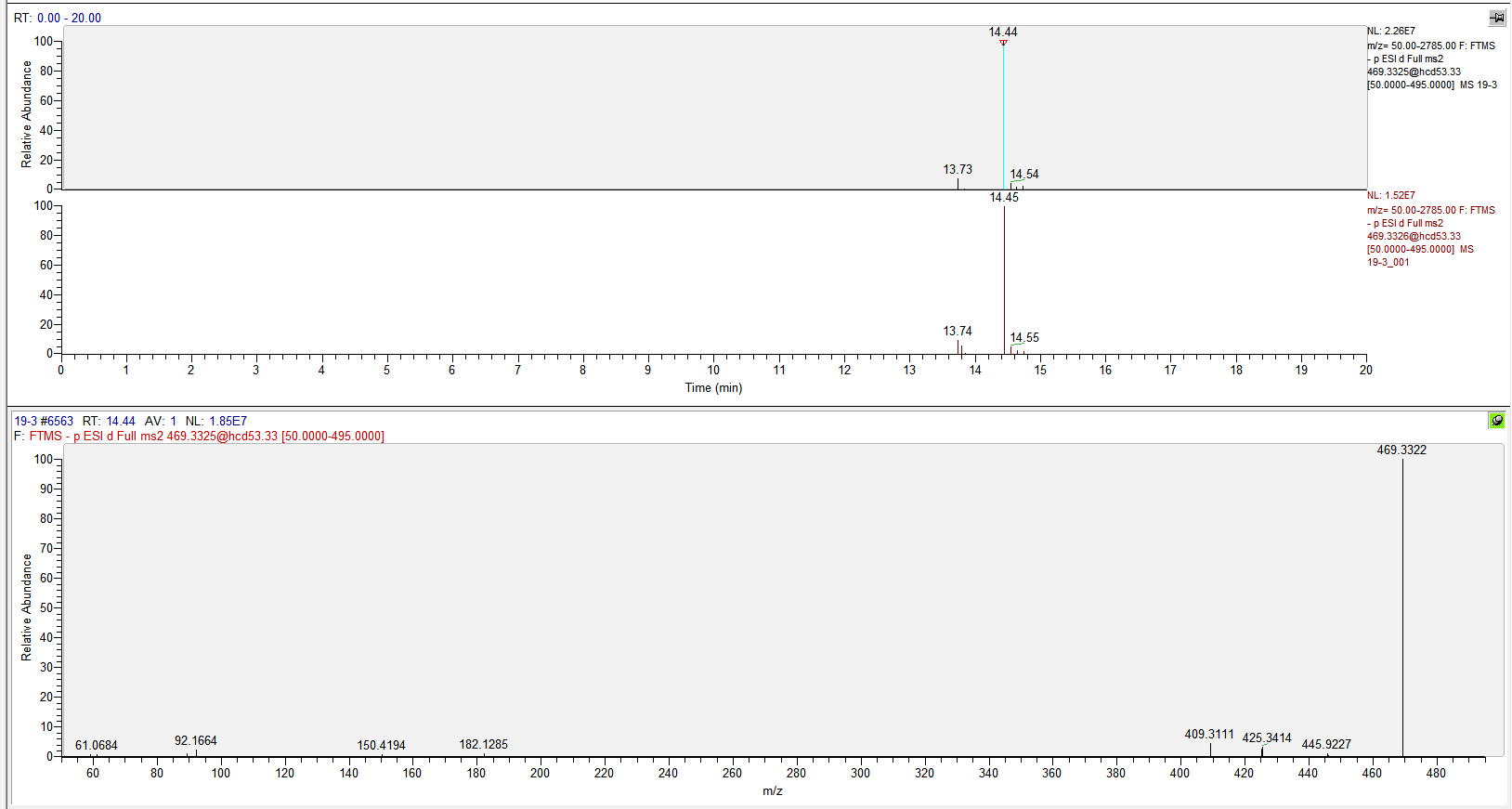


Fig. S1 18α-Glycyrrhetinic acid Standard UHPLC-Q-Orbitrap (R.T. = 14.44 min; *m/z*: 469, 425, 409)

Standard 18β- glycyrrhetinic acid


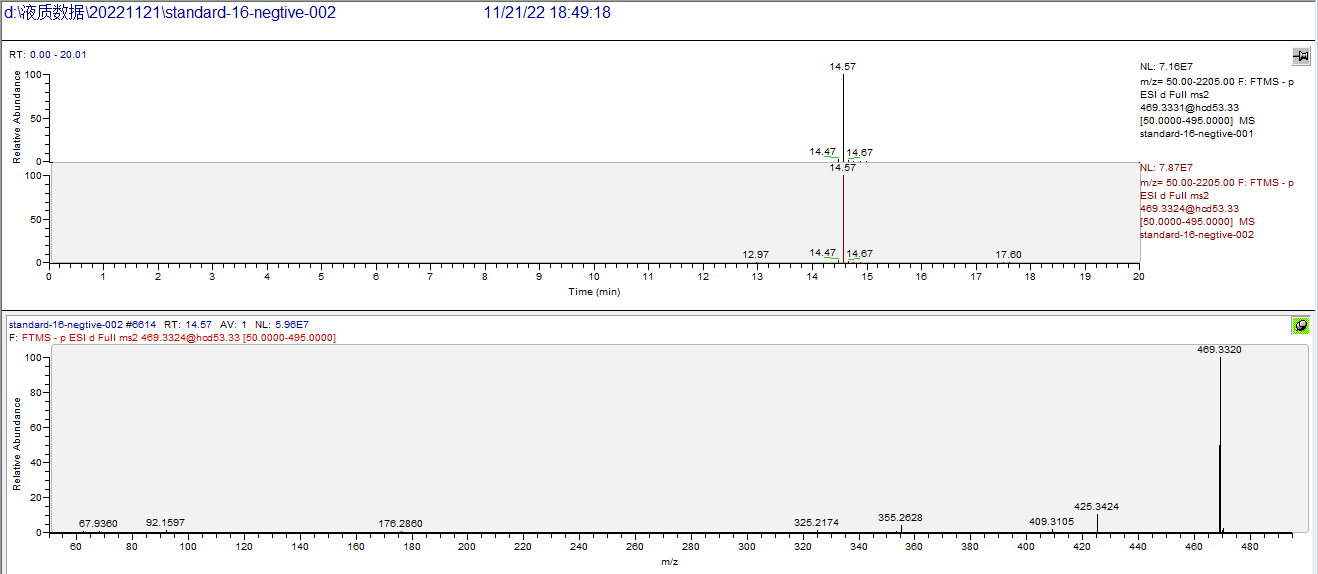


Fig. S2 18β-Glycyrrhetinic acid standard的UHPLC-Q-Orbitrap (R.T. = 14.57 min; *m/z*: 469, 425, 409, 355)

Sample


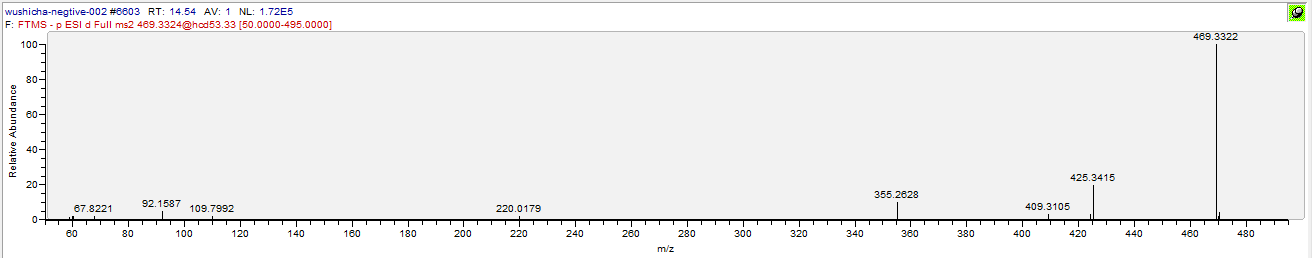


Fig. S3 UHPLC-Q-Orbitrap of sample

The chromatographic conditions are manily based on the literature ^[1]^.

**Reference**

[1] Tsai, T.H.; Chen, C.F. High-performance liquid chromatographic determination of 18 alpha-glycyrrhetinic acid and 18 beta-glycyrrhetinic acid in rat plasma: application to pharmacokinetic study. *J Chromatogr*. **1991**, 567, 405-414.


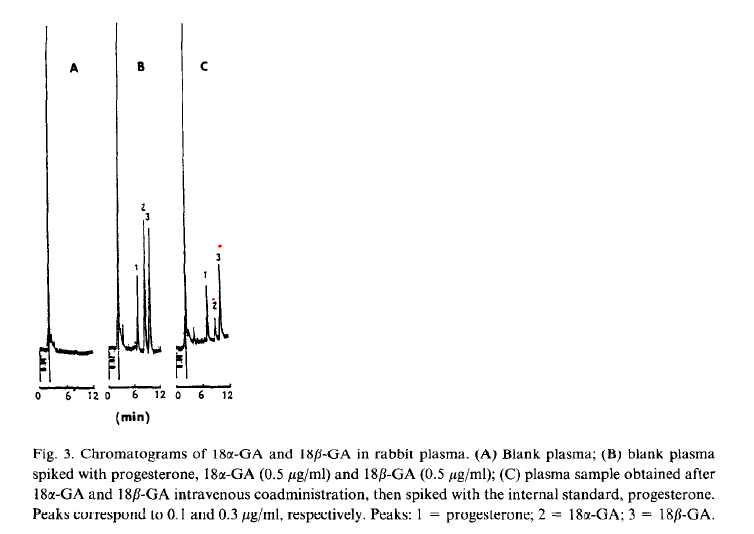

Supplement: Supplementary file 44 — Additional file 44. Identification of 18β-glycyrrhetinic acid and exclusion of 18α- glycyrrhetinic acid. [file 13020_2023_829_MOESM44_ESM.docx]
